# Global, regional, and national burden and trends of migraine among youths and young adults aged 15–39 years from 1990 to 2021: findings from the global burden of disease study 2021

**DOI:** 10.1186/s10194-024-01832-0

**Published:** 2024-08-12

**Authors:** Zhi-feng Chen, Xiang-meng Kong, Cheng-hao Yang, Xin-yu Li, Hong Guo, Zhao-wei Wang

**Affiliations:** 1 Department of Anesthesiology, School of Medicine, Shanghai Putuo People’s Hospital, Tongji University, Shanghai, China; 2 Department of Cardiology, Shanghai Ninth People’s Hospital, Shanghai Jiao Tong University School of Medicine, Huangpu District, No.639 Zhizaoju Road, Shanghai, 200011 China; 3 Department of Vascular Surgery, School of Medicine, Shanghai Putuo People’s Hospital, Tongji University, Shanghai, China; 4 Department of Plastic and Reconstructive Surgery, Shanghai Ninth People’s Hospital, Shanghai Jiao Tong University, Shanghai, PR China; 5 Department of Gynecology and Obstetrics, Tongji Hospital, Tongji University School of Medicine, Shanghai, China; 6 Department of Neurology, Qianjiang Central Hospital of Hubei Province, Hubei, China; 7 Department of Neurosurgery, Shanghai Ninth People’s Hospital, Shanghai JiaoTong University School of Medicine, No.639 Zhizaoju Road, Huangpu District, Shanghai, 200011 China

**Keywords:** Migraine, Global burden of disease, Disability-adjusted life years, Socio-demographic index

## Abstract

**Background:**

Migraine, a widespread neurological condition, substantially affects the quality of life, particularly for adolescents and young adults. While its impact is significant, there remains a paucity of comprehensive global research on the burden of migraine in younger demographics. Our study sought to elucidate the global prevalence, incidence, and disability-adjusted life-years (DALYs) associated with migraine in the 15–39 age group from 1990 to 2021, utilizing data from the Global Burden of Disease (GBD) 2021 study.

**Methods:**

Our comprehensive study analyzed migraine data from the GBD 2021 report, examining the prevalence, incidence, and DALYs across 204 countries and territories over a 32-year span. We stratified the information by age, sex, year, geographical region, and Socio-demographic Index (SDI). To evaluate temporal trends in these metrics, we employed the estimated annual percentage change (EAPC) calculation.

**Results:**

Between 1990 and 2021, the worldwide prevalence of migraine among 15–39 year-olds increased substantially. By 2021, an estimated 593.8 million cases were reported, representing a 39.52% rise from 425.6 million cases in 1990. Global trends showed increases in age-standardized prevalence rate, incidence rate, and DALY rate for migraine during this period. The EAPC were positive for all three metrics: 0.09 for ASPR, 0.03 for ASIR, and 0.09 for DALY rate. Regions with medium SDI reported the highest absolute numbers of prevalent cases, incident cases, and DALYs in 2021. However, high SDI regions demonstrated the most elevated rates overall. Across the globe, migraine prevalence peaked in the 35–39 age group. Notably, female rates consistently exceeded male rates across all age categories.

**Conclusion:**

The global impact of migraine on youths and young adults has grown considerably from 1990 to 2021, revealing notable variations across SDI regions, countries, age groups, and sexes. This escalating burden necessitates targeted interventions and public health initiatives, especially in areas and populations disproportionately affected by migraine.

**Supplementary Information:**

The online version contains supplementary material available at 10.1186/s10194-024-01832-0.

## Introduction

Migraine, a pervasive neurological disorder, exacts a considerable toll on both individuals and society [1]. Although it affects people across all age groups, its prevalence notably surges among adolescents and young adults, significantly compromising their quality of life [2, 3]. The 15-39 age group is particularly vulnerable to the onset and impact of migraine, as this is a critical period for educational attainment, career development, and social relationships [2, 3]. The long-term consequences of migraine, such as increased risk of comorbidities and reduced quality of life, further highlight the importance of focusing on this demographic. By understanding the epidemiology of migraine in youths and young adults, we can implement early detection, prevention, and management strategies to mitigate the long-term burden of the condition and improve outcomes for this vulnerable demographic.

In 2019, migraine emerged as a primary contributor to global disability-adjusted life years (DALYs), with a particularly pronounced impact on females aged 15-39 years [4]. This debilitating condition is characterized by recurrent episodes of moderate to severe headaches, frequently accompanied by a range of neurological and systemic symptoms. These may include heightened sensitivity to light and sound, as well as gastrointestinal disturbances [5, 6]. Various factors can precipitate migraine attacks, with stress, exhaustion, and specific dietary elements being common triggers [7]. Despite its widespread occurrence, migraine often goes undiagnosed and inadequately treated. This issue is especially prevalent in low- and middle-income nations, where there is a scarcity of research, education, and clinical resources dedicated to this neurological disorder [8].

While existing research has explored migraine's global impact, there's a notable gap in studies focusing on the 15-39 age group [2, 7]. Two recent studies have addressed this age group using data from the GBD 2019 study [2, 3]. However, our study aims to provide the most up-to-date and comprehensive analysis of migraine epidemiology in youths and young adults by utilizing the latest GBD 2021 dataset and employing advanced statistical methods, such as EAPC, decomposition analysis, and Bayesian age-period-cohort (BAPC) projections. These methods allow us to evaluate temporal trends, disentangle the relative contributions of population factors, and offer a forward-looking perspective on migraine burden in this age group. Moreover, our study has the unique advantage of capturing the potential impact of the COVID-19 pandemic on migraine burden among youths and young adults, which was not possible in previous studies using pre-2020 data. By incorporating the most recent data from 2021, we can provide valuable insights into how the global health crisis may have influenced migraine prevalence, incidence, and DALYs in this age group.

## Method

### Data acquisition and download

The migraine data for youths and young adults used in this study were obtained from the GBD 2021, which provides the most up-to-date estimates for 369 diseases, injuries, and impairments, as well as 88 risk factors, across 204 countries and territories grouped into 21 region [9]. For the purpose of this study, we utilized the GBD 2021 classification, which divides the world into 21 geographic regions based on epidemiological similarities and geographic proximity [4, 10]. This categorization allows for a more nuanced understanding of the variations in disease burden across different parts of the world, enabling the development of targeted public health policies and interventions. The 21 regions are as follows: Andean Latin America, Australasia, Caribbean, Central Asia, Central Europe, Central Latin America, Central Sub-Saharan Africa, East Asia, Eastern Europe, Eastern Sub-Saharan Africa, High-income Asia Pacific, High-income North America, North Africa and Middle East, Oceania, South Asia, Southeast Asia, Southern Latin America, Southern Sub-Saharan Africa, Tropical Latin America, Western Europe, and Western Sub-Saharan Africa. This regional classification system has been consistently used in previous iterations of the GBD study and has proven to be an effective way of analyzing and comparing health metrics across geographically and epidemiologically distinct areas [4, 10]. For this investigation, we extracted data specific to migraine in youths and young adults, including prevalence, incidence, and DALYs. These metrics were accompanied by their respective 95% uncertainty intervals (UI), providing a measure of statistical confidence. The calculation of DALYs incorporated both years lived with disability and years of life lost, offering a holistic view of the disease burden (refer to Supplementary Methods for detailed methodology).

To contextualize our findings, we utilized the socio-demographic index (SDI), a composite measure that evaluates regional development based on income, education, and fertility indicators [11]. The GBD 2021 categorized countries and territories into five SDI strata, ranging from low to high development levels. All data used in this study is publicly accessible through the Global Health Data Exchange platform (https://ghdx.healthdata.org/gbd-2021/sources). The University of Washington's Institutional Review Board granted a waiver of informed consent for GBD studies. Our research adheres to the STROCSS criteria for reporting [12].

### Disease definition

In the GBD 2021 classification system, headache disorders are categorized at the third level, falling under the broader categories of neurological disorders (level 2) and non-communicable diseases (level 1). Migraine, specifically, is identified as a distinct entity at level 4 within the headache disorders group. Migraine is classified as a primary headache disorder, typically manifesting as recurring, moderate to severe headaches. These headaches are often characterized by their unilateral nature and pulsating quality. For diagnostic and coding purposes, the International Classification of Diseases (ICD) provides specific identifiers for migraine. In the ICD-9, migraine is coded under the range 346–346.93, while in the ICD-10, it falls under G43–G43.919 [13].

### Statistical analysis

To analyze trends in age-standardized rates (ASR) of migraine incidence, mortality, DALYs, and prevalence, we employed the EAPC method. This approach involves a regression model expressed as [14]:

$$In(ASR)=\alpha+\beta\mathrm X+\mathrm e$$

The natural logarithm of the age-standardized rate is represented by ln(ASR) in this equation. X stands for the calendar year under consideration. The y-intercept is denoted by α, while β indicates the slope, which represents the trend over time. Any error in the model is accounted for by e. The EAPC is expressed as 100 × [exp(β)–1], indicating yearly percentage change. We analyzed trends using the EAPC and its 95% CI. An increasing trend was identified when both EAPC and lower CI limit were positive. A decreasing trend was noted when both EAPC and upper CI limit were negative. Trends were considered stable if neither condition was met. Gaussian process regression and Pearson correlation coefficient were used to examine relationships between EAPCs, age-standardized rates, and SDI. We applied decomposition methodology to break down migraine incidence, prevalence, and DALYs by population age structure, growth, and epidemiologic changes [15–18].

We employed the BAPC method to estimate marginal posterior distributions. This approach utilizes integrated nested Laplace approximations (INLA). BAPC sidesteps common challenges associated with Markov chain Monte Carlo methods in Bayesian analysis. The BAPC method has gained widespread use in analyzing trends of chronic diseases and forecasting future disease burden [19–22]. We conducted our analysis using the 'INLA' and 'BAPC' R packages, following established protocols [19–22]. We conducted our analyses and created visualizations using two primary tools. The World Health Organization's Health Equity Assessment Toolkit was employed for equity-focused analyses. Additionally, we utilized R statistical software, specifically version 4.2.1, for data processing and statistical computations.

## Results

### Global level

A significant rise in migraine prevalence, incidence, and DALYs was observed among youths and young adults globally (Table 1, Tables S1-S2). The global burden of migraine among individuals aged 15-39 witnessed a significant increase from 1990 to 2021. By 2021, the estimated prevalence reached 593,843,983.4 cases (95% UI: 491,852,422.4-710,571,345.4), representing a 39.52% rise from 425,603,893.1 cases (95% UI: 351,207,584.1-509,636,137.2) in 1990. The global ASPR of migraine climbed from 19,418 per 100,000 population in 1990 to 19,962.3 per 100,000 population in 2021. Concurrently, incident cases surged by 36.82%, escalating from 31,939,565.5 in 1990 to 43,699,829.6 in 2021. DALYs exhibited a parallel trend, increasing by 39.46% from 15,755,634.7 in 1990 to 21,973,190.4 in 2021.

**Table 1:** Prevalence of migraine between 1990 and 2021 in 15 to 39 years at the Global and Regional Level

Location	1990		2021		EAPC_95%CI
	Number(95%UI)	ASR(95%UI)	Number(95%UI)	ASR(95%UI)	
Global	425,603,893.1 (351,207,584.1–509,636,137.2)	19,418 (16,023.7–23,252)	593,843,983.4 (491,852,422.4–710,571,345.4)	19,962.3 (16,533.8–23,886.1)	0.09 (0.07–0.1)
High SDI	77,421,395 (64,527,377.2–92,178,060.3)	22,313.9 (18,597.7–26,567)	79,711,684.7 (66,387,520.3–95,452,433.9)	22,565.8 (18,793.8–27,021.9)	0.02 (-0.03–0.06)
High-middle SDI	81,784,166.3 (67,356,251.9–97,913,634.4)	18,072.4 (14,884.1–21,636.6)	83,196,755 (69,027,834.8–99,398,101.3)	18,896.9 (15,678.6–22,576.8)	0.16 (0.14–0.17)
Middle SDI	140,698,028.1 (116,186,174.4–167,753,825.2)	18,694.1 (15,437.3–22,289)	187,106,845.4 (154,961,311.5–223,692,294)	20,173.5 (16,707.6–24,118)	0.24 (0.23–0.25)
Low-middle SDI	93,003,156.7 (76,502,935.2–111,979,925.4)	20,512.4 (16,873.2–24,697.8)	164,907,677.5 (135,796,096.5–197,476,732)	20,549.4 (16,921.8–24,607.9)	-0.01 (-0.02–0)
Low SDI	32,302,327.8 (26,391,086.4–38,997,797.2)	17,526.2 (14,319–21,159)	78,462,379.5 (64,148,339.5–94,490,940.9)	17,472.5 (14,285–21,041.9)	-0.01 (-0.02–0)
Andean Latin America	2,104,424.9 (1,763,951–2,527,918.8)	13,608.8 (11,407.1–16,347.5)	3,930,498.2 (3,207,613.2–4,787,942.3)	14,514.6 (11,845.2–17,681)	0.25 (0.19–0.3)
Australasia	1,586,829.6 (1,279,886.5–1,924,703.5)	19,461 (15,696.7–23,604.8)	2,052,856 (1,668,935.3–2,496,704.3)	19,605.3 (15,938.8–23,844.2)	0.01 (0–0.02)
Caribbean	2,970,983.3 (2,379,307.8–3,636,616.3)	19,986.9 (16,006.5–24,464.9)	3,614,002.3 (2,917,967.8–4,404,488.6)	19,854.2 (16,030.4–24,196.9)	-0.02 (-0.02–0.02)
Central Asia	5,303,538.8 (4,255,977.7–6,546,812)	18,639.2 (14,957.5–23,008.6)	7,030,964.2 (5,687,549.3–8,667,574.8)	18,806 (15,212.7–23,183.5)	0.01 (0–0.03)
Central Europe	8,852,207.9 (7,278,289.4–10,686,840.8)	18,895.4 (15,535.8–22,811.5)	6,686,605 (5,520,562.3–8,066,130.4)	19,093.8 (15,764.1–23,033.1)	0.06 (0.05–0.08)
Central Latin America	13,244,198.8 (11,017,799.7–15,863,381.4)	19,400.5 (16,139.2–23,237.2)	19,892,752 (16,358,682.1–24,052,302.4)	19,664.1 (16,170.7–23,775.9)	0.05 (0.04–0.06)
Central Sub-Saharan Africa	3,530,714.8 (2,829,201.3–4,376,135.9)	17,005.5 (13,626.7–21,077.4)	9,197,259.2 (7,376,247.2–11,385,771.2)	17,001.7 (13,635.4–21,047.3)	0 (0–0)
East Asia	84,582,556 (69,133,231.5–100,747,871.8)	14,951.7 (12,220.7–17,809.2)	78,973,477.8 (65,089,594.1–93,838,388.6)	16,485.5 (13,587.3–19,588.5)	0.31 (0.27–0.34)
Eastern Europe	16,422,489.4 (13,688,703.2–19,617,944.6)	19,147.5 (15,960.1–22,873.1)	12,836,712.4 (10,765,891.7–15,284,030.8)	19,398.6 (16,269.2–23,097)	0.08 (0.06–0.1)
Eastern Sub-Saharan Africa	8,610,438.3 (7,020,409.3–10,336,669.7)	12,146.2 (9903.3–14,581.3)	21,417,814.6 (17,440,224.4–25,763,396)	12,225.8 (9955.3–14,706.4)	0.05 (0.04–0.07)
High-income Asia Pacific	10,927,854.5 (8,953,649.2–13,087,458.2)	16,190.6 (13,265.6–19,390.2)	8,245,025.7 (6,838,946.8–9,847,197.4)	16,314.1 (13,531.9–19,484.2)	-0.02 (-0.03–0)
High-income North America	28,590,762.4 (23,804,003.9–33,634,974.1)	25,231.1 (21,006.8–29,682.6)	30,518,152.5 (25,384,649.3–36,080,785.4)	24,774.4 (20,607.1–29,290.1)	-0.02 (-0.11–0.07)
North Africa and Middle East	28,375,170.1 (23,201,519.4–34,514,542.5)	21,202.5 (17,336.6–25,789.9)	54,840,333.5 (45,580,153.1–66,520,799.5)	21,568.1 (17,926.2–26,161.9)	0.07 (0.05–0.08)
Oceania	514,985.3 (415,338.5–640,330.9)	19,386.7 (15,635.5–24,105.4)	1,101,346.4 (888,266.6–1,364,189.4)	19,546.8 (15,765–24,211.7)	0.02 (0.02–0.02)
South Asia	88,417,118.3 (72,862,856.6–106,014,603.6)	20,485.1 (16,881.4–24,562.2)	163,235,078.8 (134,735,029.6–194,046,192.3)	20,638.4 (17,035–24534)	-0.01 (-0.04–0.02)
Southeast Asia	45,321,111.2 (37,227,303.3–54,903,115.9)	23,005.1 (18,896.7–27,868.9)	63,182,389 (52,093,831–76595588.9)	22,782.7 (18,784.3–27,619.3)	-0.04 (-0.05–0.03)
Southern Latin America	3,102,912.5 (2,532,496.5–3,812,065.7)	16,263.3 (13,273.6–19,980.2)	4,353,041.3 (3,557,241.7–5,302,131.4)	16,875.1 (13,790.1–20,554.3)	0.17 (0.14–0.19)
Southern Sub-Saharan Africa	3,811,721.3 (3,125,730.9–4,588,833.5)	17,634.4 (14,460.7–21,229.6)	6,067,359.5 (4,973,956.3–7,242,314.9)	17,826.3 (14,613.8–21,278.4)	0.03 (0.02–0.04)
Tropical Latin America	16,537,426.4 (13,770,071.7–19,705,613)	25,713.9 (21,411–30,640.1)	22,819,085.3 (19,005,801.4–27,206,963.2)	25,839.9 (21,521.8–30,808.6)	0.07 (0.02–0.12)
Western Europe	38,052,601 (31,439,307.5–45,938,651.1)	26,403.4 (21,814.7–31,875.3)	34,604,089.3 (28,717,872–41,667,102.1)	26,665.2 (22,129.4–32,107.8)	0.03 (0–0.07)
Western Sub-Saharan Africa	14,743,848.2 (12,005,790.9–18,085,222.7)	20,599.4 (16,773.9–25,267.9)	39,245,140.3 (31,779,582.5–47,599,003.9)	20,524.8 (16,620.4–24,893.8)	-0.01 (-0.02–0.01)

**Fig. 1:**
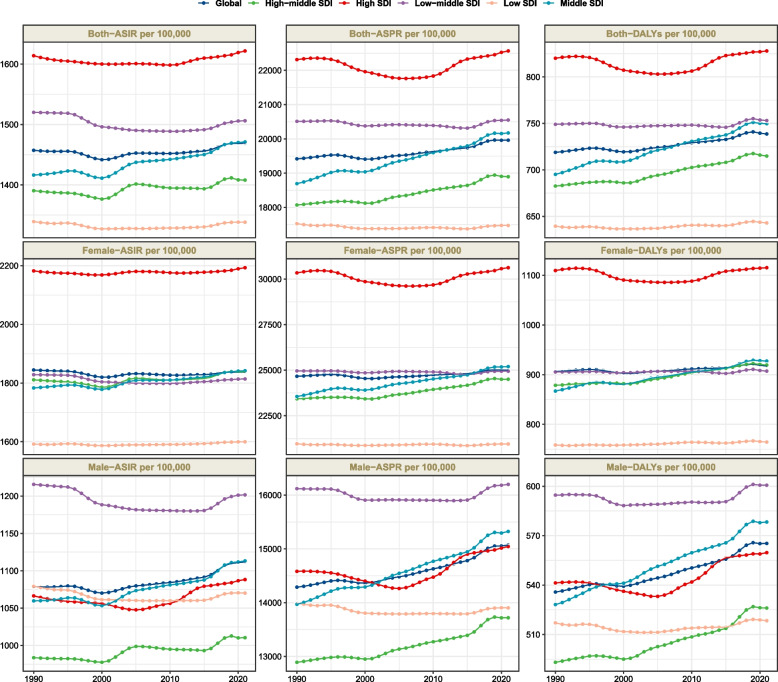
Trends in migraine prevalence, incidence and disability-adjusted life-years from 1990 to 2021

**Fig. 2:**
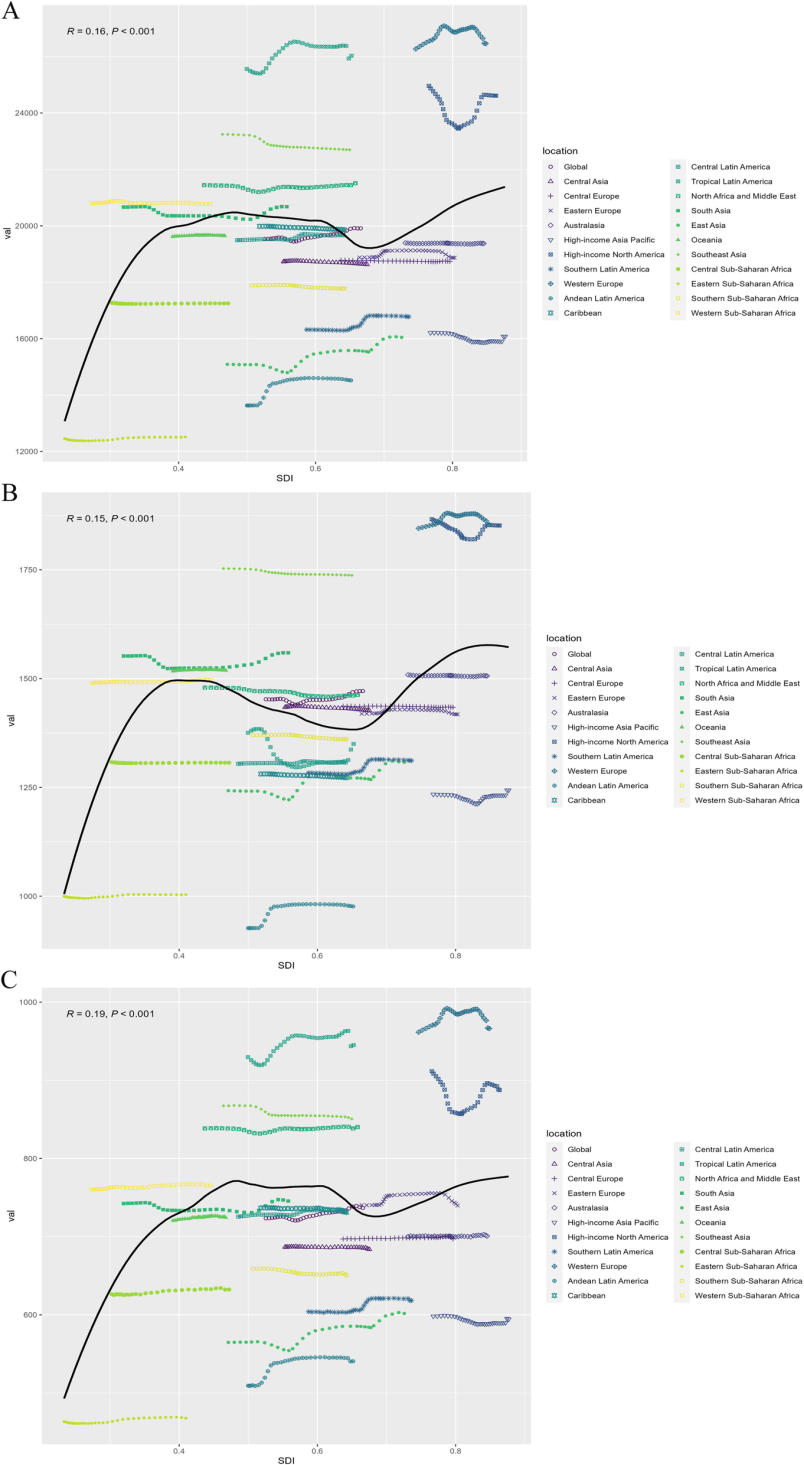
The associations between the sociodemographic index and migraine across 21 GBD regions. **A** Association between age-standardized migraine prevalence rate and sociodemographic index. **B** Association between age-standardized migraine incidence rate and sociodemographic index. **C** Association between age-standardized migraine DALYs rate and sociodemographic index

**Fig. 3:**
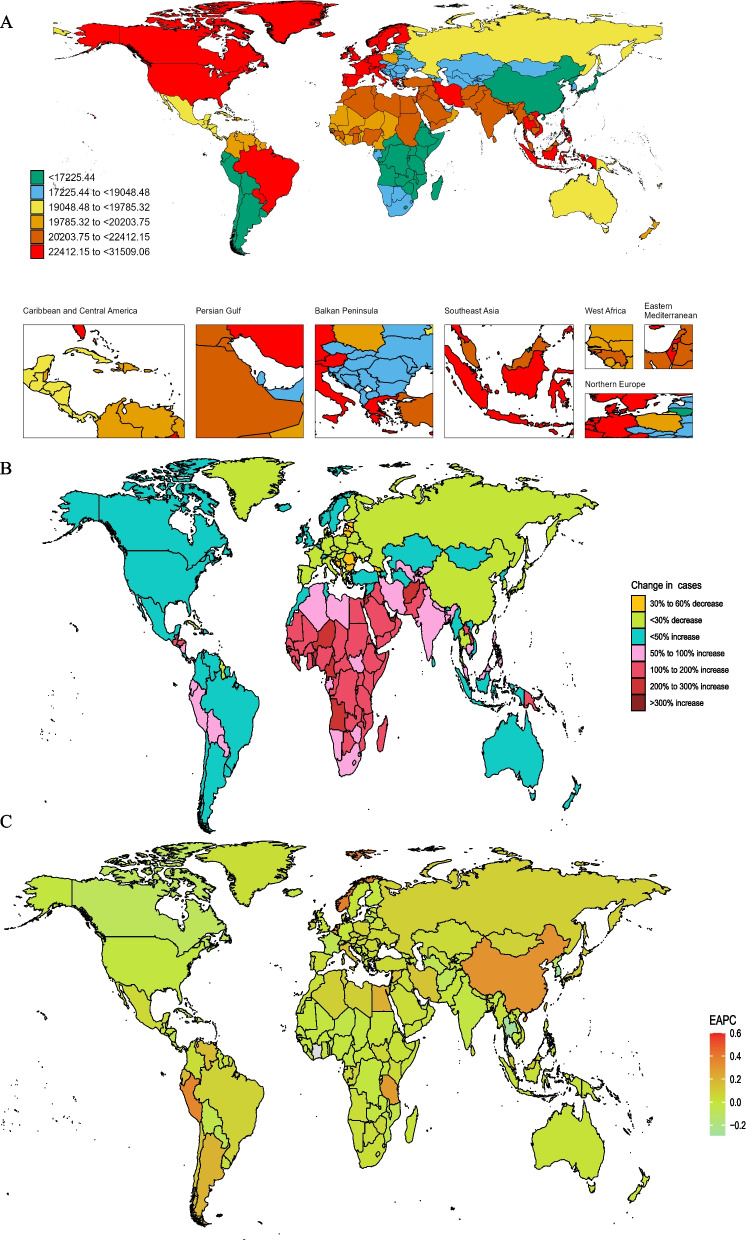
**A** The global disease burden of migraine prevalence rate for both sexes in 204 countries and territories. **B **Change prevalence cases of migraine for both sexes in 204 countries and territories. **C** EAPC for migraine prevalence

The period from 1990 to 2021 saw consistent upward trajectories in global ASPR, ASIR, and age-standardized DALY rate of migraine among youths and young adults (Fig. 1). The EAPCs were calculated at 0.09 (95% CI: 0.07-0.1) for ASPR, 0.03 (95% CI: 0.01-0.04) for ASIR, and 0.09 (95% CI: 0.08-0.11) for age-standardized DALY rate (Fig. 1, Table 1, Tables S1-S2). Although females consistently demonstrated higher ASPR, ASIR, and age-standardized DALY rates, males experienced notable increases in these metrics from 1990 to 2021 (FigS1, FigS2, FigS3). This comprehensive analysis underscores the escalating impact of migraine on younger populations, with far-reaching implications for both sex across diverse global regions.

### SDI regional level

The Middle SDI region emerged as the epicenter of migraine burden among youths and young adults in 2021, recording the highest absolute cases. Prevalence reached 187,106,845.4 (95%UI:154,961,311.5-223,692,294), incidence stood at 13,643,395.8 (95%UI: 11,251,240.6-16,821,849.5), and DALYs totaled 6,953,719.5 (95% UI: 728,469.3-15,548,979.6). Despite this, high SDI regions exhibited the most elevated rates of prevalence, incidence, and DALYs in the same year (Fig. 1). From 1990 to 2021, the middle SDI region experienced a rapid surge in ASPR, ASIR, and age-standardized DALY rate. The corresponding EAPCs were 0.24 (95% CI: 0.23-0.25), 0.13 (95% CI: 0.11-0.15), and 0.25 (95% CI: 0.24-0.26), respectively.

Across all SDI regions, females consistently outpaced males in ASPR, ASIR, and DALY rates. In non-High SDI regions, the male-to-female ASPR ratio remained below 1, decreasing progressively with age. Contrastingly, the High SDI region witnessed an initial decrease followed by a gradual increase after the 20-24 age group (Fig. S1). Similar trends were observed in male-to-female ratios of ASIR and age-standardized DALY rates (Fig. S2-S3). These patterns suggest that sex disparities in migraine burden narrow with age in High SDI regions, while widening elsewhere. Across all SDI areas, ASPR and DALY rates climbed steadily with age, peaking at 35-39 years, whereas ASIR reached its apex at 15-19 years (Fig. S4-S6).

### GBD regional level

South Asia emerged as the epicenter of migraine prevalence, recording an estimated 5,886,568.9 cases (95% UI, 549,405.8 to 13,362,984.9), while Oceania reported the lowest at 40,614.6 cases (95% UI, 3,858.2 to 93,888.1). Among the 21 regions analyzed, 16 exhibited increases in absolute numbers of prevalence, incidence, and DALYs due to migraine in youths and young adults over time. However, certain high or middle SDI regions, including High-income Asia Pacific, Western Europe, and Eastern Europe, showed decreases (Table 1, Tables S1-S2).

Over the past three decades, East Asia experienced the most dramatic surge in migraine prevalence and DALYs, with EAPCs of 0.31 (95% CI, 0.27 to 0.34) and 0.32 (95% CI, 0.28 to 0.35), respectively. In contrast, Southeast Asia witnessed the steepest decline in prevalence (EAPC: -0.04; 95% CI, -0.05 to -0.03). Andean Latin America saw the largest increase in migraine incidence (EAPC: 0.23; 95% CI, 0.17 to 0.28), while Tropical Latin America experienced the most significant decrease (EAPC: -0.14; 95% CI, -0.22 to -0.07). Western Europe reported the highest ASPR at 26,665.2 per 100,000 population (95% UI, 22,129.4 to 32,107.8) and the highest age-standardized DALYs rate at 975.4 per 100,000 population (95% UI, 101.5 to 2,203.5). Eastern Sub-Saharan Africa had the lowest rates for both metrics. High-income North America exhibited the highest ASIR at 1,827.8 per 100,000 population (95% UI, 1,527.8 to 2,211.1), while Andean Latin America reported the lowest (Table 1, Tables S1-S2).

Figure 2 demonstrates positive correlations between ASPR, ASIR, and DALYs rate of migraine and the SDI. Seven regions, including Western Europe, Tropical Latin America, and High-income North America, surpassed the global mean prevalence and incidence, while eight regions exceeded the global mean DALYs rate.

### Countries level

The global landscape of migraine prevalence among 15 to 39-year-olds from 1990 to 2021 revealed striking disparities across nations. In 2021, among 204 countries analyzed, Belgium emerged as the epicenter with the highest ASPR of migraine, recording 31,509.1 cases per 100,000 population (95% UI, 26,104.1 to 38,527.4). In stark contrast, Ethiopia reported the lowest at 11,255.9 cases per 100,000 population (95% UI, 9,210.7 to 13,432.6) (Fig. 3A). In terms of absolute case numbers, Qatar witnessed an extraordinary surge of 595%, followed by Equatorial Guinea at 343%. Conversely, Georgia experienced a notable decline of 46% (Fig. 3B, Table S3). Singapore exhibited the most pronounced increase in migraine prevalence rate (EAPC, 0.6; 95% CI, 0.44 to 0.76), while the Republic of Korea showed the steepest decline (EAPC, -0.29; 95% CI, -0.35 to -0.23) between 1990 and 2021 (Fig. 3C, Table S4).

Italy topped the charts for migraine incidence in 2021, with 1,942.6 cases per 100,000 population (Table S5, FigS7). Peru demonstrated the most significant increase in migraine incidence rate (EAPC, 0.35; 95% CI, 0.26 to 0.45), whereas the Maldives experienced the most substantial decrease (EAPC, -0.35; 95% CI, -0.42 to -0.29) (Table S5, Fig. S8, Fig. 3B). Regarding DALYs, Belgium reported the highest age-standardized rate at 1,147.4 per 100,000 population in 2021 (Table S6, Fig. S10). Singapore stood out with the most marked increase in age-standardized DALY rates (EAPC, 0.55; 95% CI, 0.41 to 0.7) (Table S6, Fig. S11).

### Age and sex patterns

**Fig. 4:**
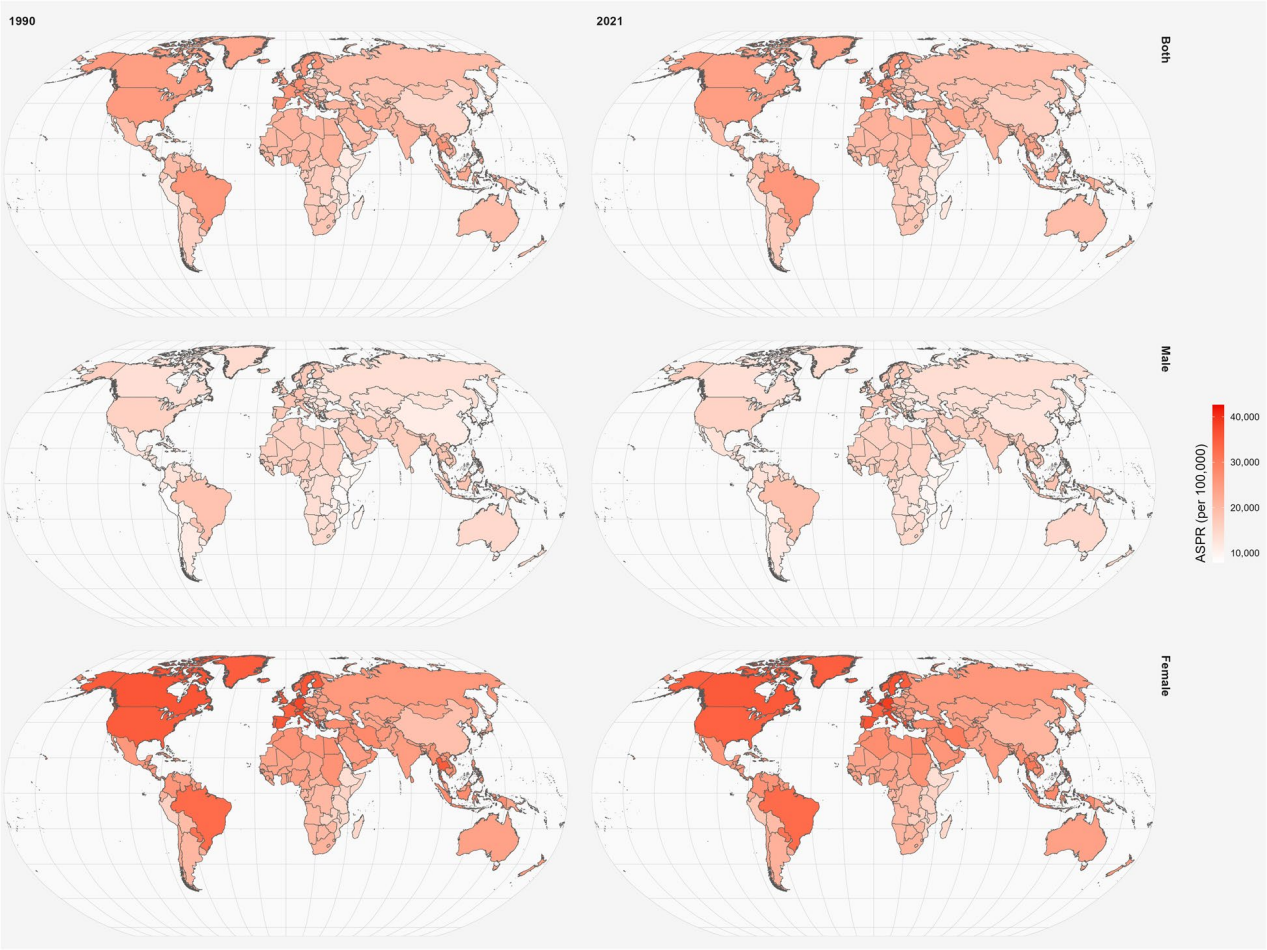
Comparison of the global disease burden of migraine prevalence in males and females across 204 countries and territories between 1990 and 2021

The global landscape of migraine in 2021 revealed distinct age and sex-related patterns. Prevalence rates reached their zenith in the 35-39 age bracket, showing an overall upward trajectory with age (Fig. S13). Across the 15-39 age spectrum, females consistently exhibited higher prevalence rates than their male counterparts (Fig. 4). Notably, migraine prevalence generally increased with age, peaking in the 30-34 age group (Fig. S13). In terms of incidence, while the ASIR remained relatively stable across age groups, the absolute number of cases was highest among 15-19 year-olds, subsequently declining with age (Fig. S14). The sex disparity persisted in ASIR from 1990 to 2021, with women consistently reporting higher rates than men (Fig. S15). The distribution of DALYs paralleled the prevalence pattern. Women shouldered a markedly heavier burden compared to men, with this disparity intensifying with age (Fig. S16-S17).

### Decomposition analysis of migraine from 1990 to 2021 according to SDI and 21 GBD regions

**Fig. 5:**
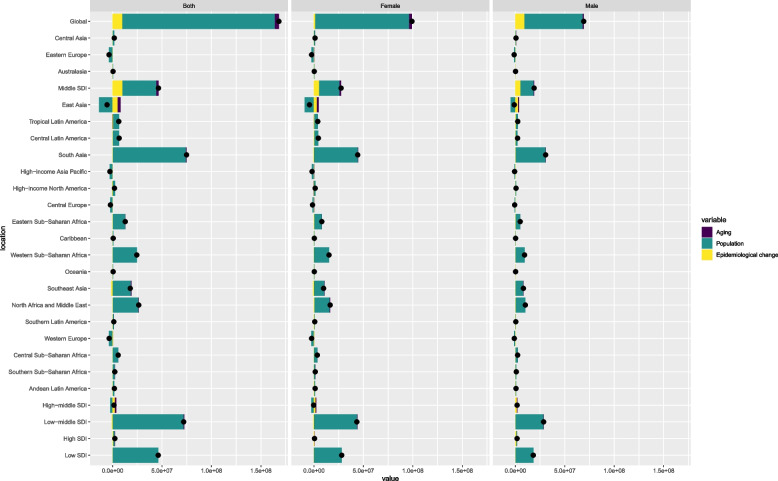
Decomposition analysis of migraine change in prevalence by SDI and 21 GBD region, 1990 to 2021

Our comprehensive decomposition analysis illuminated the relative contributions of aging, population growth, and demographically adjusted epidemiological changes to the evolving landscape of migraine incidence, prevalence, and DALYs across five SDI regions and 21 GBD regions. Notably, five regions—Central Europe, East Asia, Eastern Europe, High-income Asia Pacific, and Western Europe—exhibited a decline in overall migraine burden between 1990 and 2021. This reduction was primarily attributed to decreasing demographically adjusted epidemiological changes, offering valuable insights into the dynamics of migraine burden in these areas (Fig. 5, Fig.S18-S19). Population growth emerged as the main driver of increasing disease burden in regions experiencing growth, while epidemiological changes played a crucial role in the Middle SDI area. Despite declining population change in High-middle SDI regions and reduced aging in Low SDI regions, all five SDI regions saw an increase in total migraine prevalence from 1990 to 2021, largely attributed to population growth. The aforementioned five regions also witnessed a reduction in total migraine incidence and DALYs during this period, primarily driven by population changes (Fig S18, FigS19). This observed decrease may be partially explained by demographic shifts, particularly population aging. As these regions experience an aging population, the proportion of individuals within our study's age range (15-39) has likely diminished, potentially contributing to the apparent reduction in migraine incidence and DALYs within our target age group.

### Future burden of migraine

**Fig. 6:**
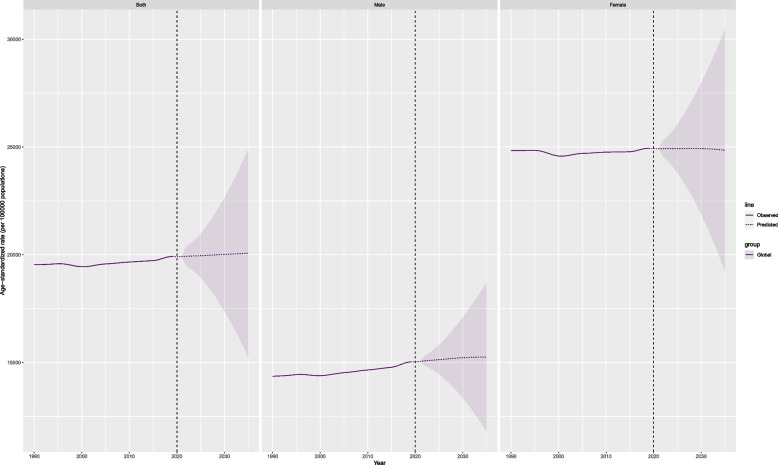
Future forecasts of GBD in migraine prevalence

Figure 6 and FigS20 illustrates the projected trends in global migraine prevalence. The graph indicates a steady rise in the number of individuals affected by migraine worldwide (FigS20). ASPR for migraine expected to top 20,000 per 100,000 population globally by 2035 (20072 per 100,000; 95%UI:15252-24891) (Fig. 6), with the ASPR for women still higher than for men, but showing a slight downward trend, with a projected global ASPR of 24847 per 100,000 (95%UI:19241-30453) for women and 15250 per 100,000 (95%UI:11788-18712) for men by 2035.Similar projections are found in the incidence of migraine, which is projected to reach 1501 per 100,000 (95%UI:1131-1871) globally by 2035, 1143 (95%UI:877-1408) in men, and 1856 (95%UI:1425-2286) in women (FigS21). Migraine DALYs are projected to reach731 per 100,000 (95%UI:554-908) globally by 2035, 565 per 100,000 (95%UI:434-695) in men, and 896 (95%UI:693-1099) in women (FigS22).

## Discussion

Our study builds upon the existing literature by utilizing the most recent GBD 2021 data, providing the latest epidemiological insights into migraine burden among youths and young adults [2, 3]. We employed advanced statistical methods, such as EAPC and decomposition analysis, to evaluate temporal trends and the relative contributions of population aging, growth, and epidemiological changes. Furthermore, our study incorporates future projections using the BAPC method, offering a forward-looking perspective to inform public health planning and resource allocation. Focusing on the 15-39 age group in our study has revealed critical insights into the burden of migraine among youths and young adults globally. This demographic represents a key transitional period in life, marked by significant milestones in education, career, and personal relationships. The high prevalence, incidence, and DALYs of migraine in this age group underscore the substantial impact of this condition on individuals during their most productive years. By shedding light on the unique challenges faced by 15-39 year-olds with migraine, our findings emphasize the urgent need for age-specific interventions and support systems to mitigate the long-term consequences of this debilitating condition and improve the quality of life for this vulnerable population. Our findings highlight the growing burden of migraine in this age group globally, emphasizing the need for targeted interventions and public health strategies to mitigate the long-term complications of this debilitating condition. The variations in migraine prevalence, incidence, and DALYs across different SDI regions and GBD regions suggest that tailored approaches are needed to address the unique challenges and needs of each population subgroup. Moreover, our study provides a foundation for future research and policy-making efforts to better understand and manage migraine in this vulnerable demographic, taking into account the potential impact of global health crises such as the COVID-19 pandemic.

From 1990 to 2021, the global prevalence of migraine experienced a remarkable surge, with an estimated 593,843,983.4 cases (95% UI: 491,852,422.4-710,571,345.4) in 2021, marking a substantial 39.52% increase from the 425,603,893.1 cases (95% UI: 351,207,584.1-509,636,137.2) recorded in 1990. This alarming trend was further reflected in the incidence of migraine among the young adult population aged 15 to 39 years, which witnessed a staggering 36.82% rise, reaching 43,699,829.6 cases (95% UI: 35,822,349.9-54,100,215.9) in 2021 compared to the figures reported in 1990. Notably, certain regions bore the brunt of this escalating burden, with Andean Latin America and East Asia experiencing the most rapid increases in migraine prevalence and incidence during this period. And we also found that there are huge differences between countries. Singapore, Italy and other high-income countries showed a sharp upward trend.

In regions such as Andean Latin America and East Asia, the sharp rise in migraine burden could be linked to the rapid pace of urbanization and economic development. As these regions undergo significant changes in living conditions, work environments, and social structures, individuals may experience increased stress levels and altered lifestyles, which are known risk factors for migraine. Urbanization has been associated with higher levels of air pollution, noise pollution, and light pollution, all of which have been linked to an increased risk of migraine [23]. In high-income countries like Singapore and Italy, the higher prevalence and incidence of migraine can be attributed to several factors. First, these countries have better healthcare systems and greater awareness of migraine, leading to improved diagnosis and reporting of the condition [24]. Second, the high-stress lifestyles and work environments in these countries, coupled with the increasing use of digital devices and screens, may contribute to the rising burden of migraine [25, 26]. Additionally, dietary changes, such as increased consumption of processed foods and caffeine, have been associated with a higher risk of migraine [27].

The relationship between migraine burden and SDI is a complex one, as evidenced by the findings from this analysis of GBD data. The heterogeneity in migraine burden across SDI regions and countries underscores the importance of tailored interventions and public health strategies. The data suggests that there is a positive correlation between SDI and migraine burden, with higher SDI regions and countries generally experiencing higher prevalence, incidence, and DALYs associated with migraine. This association can be attributed to several factors related to socioeconomic development. As countries progress along the SDI spectrum, they undergo rapid urbanization, industrialization, and lifestyle changes. These changes can lead to increased stress levels, sedentary behavior, and exposure to environmental pollutants, all of which have been identified as risk factors for migraine. Additionally, higher SDI countries often have better healthcare systems and greater awareness of migraine, leading to improved diagnosis and reporting of the condition.

Our study, utilizing the most recent GBD 2021 data, not only builds upon the findings of two previously published studies on migraine burden among youths and young adults but also offers unique insights when compared to studies focusing on other age groups [2, 3]. While our results largely align with earlier youth-focused studies, we observed several key differences and updates. Our study found a global prevalence of 593,843,983.4 cases of migraine among 15-39 year-olds in 2021, which is higher than the 566,510,278 cases reported by Li et al. for 2019, indicating a continuing upward trend in migraine prevalence [2]. We observed slightly different ASPR, with our global ASPR for 2021 at 19,962.3 per 100,000 population, compared to 19,838.6 per 100,000 reported by Yang et al. for 2019 [3]. Our more detailed analysis of migraine burden across SDI quintiles revealed that the Middle SDI region had the highest absolute number of prevalent cases in 2021, a finding not explicitly reported in previous youth-focused studies. When comparing our results to studies on other age groups, such as those focusing on older adults or children, we found notable differences in prevalence patterns and burden distribution [28]. While studies on older adults typically report a decline in migraine prevalence after middle age, our focus on the 15-39 age group captured the peak prevalence years, providing a more comprehensive picture of the disease burden during this critical life stage. Furthermore, We revealed a steeper increase in prevalence from adolescence to young adulthood compared to studies on childhood migraine, highlighting the unique challenges faced by this age group. Our country-specific analysis identified trends not previously emphasized, such as Qatar experiencing a 595% increase in prevalence from 1990 to 2021. Unlike some studies on older populations that show stabilizing or decreasing trends in certain regions, our research consistently demonstrated increasing burden across most areas for the youth and young adult demographic [28]. The incorporation of BAPC analysis and decomposition methodology provided novel insights into the temporal trends and contributing factors of migraine burden, approaches not commonly used in studies of other age groups. These methodological differences allowed us to disentangle the effects of population growth, aging, and epidemiological changes more effectively than previous research across various age categories.

This analysis of GBD data highlights the disproportionate impact of migraine on females compared to males across all age groups and regions. This finding is consistent with previous research, which suggests that hormonal factors, particularly fluctuations in estrogen levels, may play a significant role in the higher prevalence of migraine among women [29, 30]. Estrogen has been shown to influence the neurotransmitter systems involved in migraine pathophysiology, such as serotonin and glutamate. Additionally, sex disparities in healthcare access and treatment-seeking behavior may contribute to the observed differences in migraine burden between males and females. Women are more likely to seek medical attention for migraine compared to men, which may lead to higher rates of diagnosis and reporting.

Our decomposition analysis revealed an intriguing trend in several regions, including Central Europe, East Asia, Eastern Europe, and High-income Asia Pacific, where the overall migraine burden among the 15-39 age group decreased between 1990 and 2021. This reduction was primarily attributed to declining demographically adjusted epidemiological changes. This finding warrants further exploration, as it contrasts with the global trend of increasing migraine burden. Several factors may contribute to this regional decrease, including significant demographic shifts in these regions over the past three decades, such as declining birth rates and aging populations [31]. The decrease in the proportion of people aged 15-39 years could directly contribute to a reduced migraine burden in this age group. Additionally, many of these regions have undergone rapid economic growth and improvements in healthcare systems, potentially leading to better access to healthcare, increased awareness, and improved diagnostic capabilities, which may have resulted in more effective management of migraine [32]. Urbanization and changes in lifestyle factors such as diet, stress levels, and sleep patterns in these regions could also potentially influence migraine prevalence and severity [33]. Improved awareness and reporting of migraine in earlier years might have led to an initial increase in reported cases, followed by a stabilization or decrease as management strategies improved [34]. Furthermore, changes in environmental triggers specific to these regions, such as air quality improvements in some East Asian countries, could potentially impact migraine prevalence [35]. It's important to note that while these regions show a decreasing trend, they still maintain relatively high rates of migraine burden compared to global averages, suggesting that despite improvements, migraine remains a significant health concern in these areas. Further research is needed to fully understand the mechanisms behind these regional decreases and to determine if the strategies employed in these regions could be applied elsewhere to reduce the global burden of migraine among young adults. Additionally, continued monitoring of these trends is crucial to ensure that this decrease is sustained and to identify any potential reversals in the future [4].

To address the growing burden of migraine among youths and young adults, a multifaceted approach is necessary. Public health campaigns aimed at raising awareness about migraine, its triggers, and management strategies can help individuals better understand and cope with the condition [36]. These campaigns should target not only the general public but also healthcare providers, educators, and policymakers to ensure a comprehensive and coordinated response. Schools and universities can play a crucial role in promoting migraine awareness and supporting students who suffer from the condition [37]. Implementing stress management programs, encouraging regular exercise, and promoting healthy sleep habits can help reduce the risk of migraine and improve overall well-being [38]. Healthcare systems should prioritize the training of healthcare professionals to accurately diagnose and effectively treat migraine, particularly in regions where the condition is underdiagnosed [39]. Increasing access to specialized headache clinics and telemedicine services can help bridge the gap in migraine care, especially in underserved areas. Furthermore, research efforts should focus on identifying novel therapeutic targets and developing more effective and accessible treatments for migraine. Advances in personalized medicine and the use of biomarkers may help optimize treatment strategies and improve patient outcomes.

### Limitations

This study has several important limitations that warrant consideration. Firstly, our analysis is confined to migraine in youths and young adults as a level four disease, rather than encompassing the broader category of level three "headache disorders." This focused approach, while allowing for a detailed examination of migraine, may not capture the full spectrum of headache-related burden.

Secondly, the quality and availability of healthcare data vary significantly across regions. In less developed countries with limited healthcare infrastructure, issues of misdiagnosis and underdiagnosis are likely to occur, potentially leading to an underestimation of the true migraine burden.

Furthermore, this study, like all analyses based on GBD data, is subject to limitations in case ascertainment. The identification of migraine cases in the GBD study relies on various data sources and coding systems, which may not always align perfectly with clinical diagnostic criteria. This discrepancy could potentially lead to under- or over-estimation of migraine prevalence and burden in certain regions or populations.

## Conclusions

The global burden of migraine among individuals aged 15-39 has escalated significantly from 1990 to 2021, revealing stark disparities across SDI regions, countries, age groups, and sexes. The Middle SDI region reported the highest absolute numbers of prevalent, incident, and DALY cases in 2021, while the High SDI region exhibited the highest rates. Globally, migraine prevalence peaked in the 35-39 age group, consistently higher in females across all age brackets. Factors such as rapid urbanization, economic development, and high-stress lifestyles may contribute to the increasing migraine burden in certain regions. This necessitates targeted interventions and public health strategies, particularly in disproportionately affected areas. Future research should prioritize identifying risk factors, enhancing diagnostic and treatment options, and developing effective prevention strategies to mitigate the global migraine burden among youths and young adults.

### Supplementary Information

Supplementary Material 1: Fig. S1. Ratio of male to female prevalence of migraine in different age subgroups.

Supplementary Material 2: Fig. S2. Ratio of male to female incidence of migraine in different age subgroups.

Supplementary Material 3: Fig. S3 Ratio of male to female DALYs of migraine in different age subgroups.

Supplementary Material 4: Fig. S4 Prevalence rate of migraine in males and females across different age groups in five SDI regions from 1990 to 2021

Supplementary Material 5: Fig. S5 Incidence rate of migraine in males and females across different age groups in five SDI regions from 1990 to 2021

Supplementary Material 6: Fig. S6 DALYs rate of migraine in males and females across different age groups in five SDI regions from 1990 to 2021

Supplementary Material 7: Fig. S7：The global disease burden of migraine incidence rate for both sexes in 204 countries and territories.

Supplementary Material 8: Fig. S8：EAPC for migraine incidence

Supplementary Material 9: Fig. S9: Change incidence cases of migraine for both sexes in 204 countries and territories.

Supplementary Material 10: Fig. S10：The global disease burden of migraine DALYs rate for both sexes in 204 countries and territories.

Supplementary Material 11: Fig. S11：EAPC for migraine DALYs

Supplementary Material 12: Fig. S12: Change DALYs cases of migraine for both sexes in 204 countries and territories.

Supplementary Material 13: Fig. S13: Prevalence and prevalence rates by age for men and women worldwide and in 5 SDI regions, 2021

Supplementary Material 14: Fig. S14: Incidence and incidence rates by age for men and women worldwide and in 5 SDI regions, 2021

Supplementary Material 15: Fig. S15 Comparison of the global disease burden of migraine incidence in males and females across 204 countries and territories between 1990 and 2021

Supplementary Material 16: Fig. S16 Comparison of the global disease burden of migraine DALYs in males and females across 204 countries and territories between 1990 and 2021

Supplementary Material 17: Fig. S17: DALYs and DALYs rates by age for men and women worldwide and in 5 SDI regions, 2021

Supplementary Material 18: Fig. S18: Decomposition analysis of migraine change in incidence by SDI and 21 GBD region, 1990 to 2021.

Supplementary Material 19: Fig.S19: Decomposition analysis of migraine change in DALYs by SDI and 21 GBD region, 1990 to 2021.

Supplementary Material 20: Fig. S20: Future forecasts of GBD in migraine prevalence.

Supplementary Material 21: Fig. S21: Future forecasts of GBD in migraine incidence.

Supplementary Material 22: Fig. S22: Future forecasts of GBD in migraine DALYs.

Supplementary Material 23: Table S1 Incidence of Migraine Between 1990 and 2021 in 15 to 39 years at the Global and Regional Level

Supplementary Material 24: Table S2 Disability-adjusted life years of Migraine Between 1990 and 2021 in 15 to 39 years at the Global and Regional Level

Supplementary Material 25: Table S3 Percentage change in migraine prevalence in 204 countries

Supplementary Material 26: Table S4 Prevalence of Migraine Between 1990 and 2021 in 15 to 39 years at the 204 Countries Level

Supplementary Material 27: Table S5 Incidence of Migraine Between 1990 and 2021 in 15 to 39 years at the 204 Countries Level

Supplementary Material 28: Table S6: The global disease burden of migraine DALYs for both sexes in 204 countries and territories.

Supplementary Material 29.

## Data Availability

GBD study 2021 data resources were available online from the Global Health Data Exchange (GHDx) query tool ( http://ghdx.healthdata.org/gbd-results-tool).
