# Supplementary figures and images for: Global, regional, and national burden and trends of migraine among youths and young adults aged 15–39 years from 1990 to 2021: findings from the global burden of disease study 2021
Source: J Headache Pain. 2024 Aug 12;25(1):131. doi: 10.1186/s10194-024-01832-0 (PMC11318134; doi:10.1186/s10194-024-01832-0)

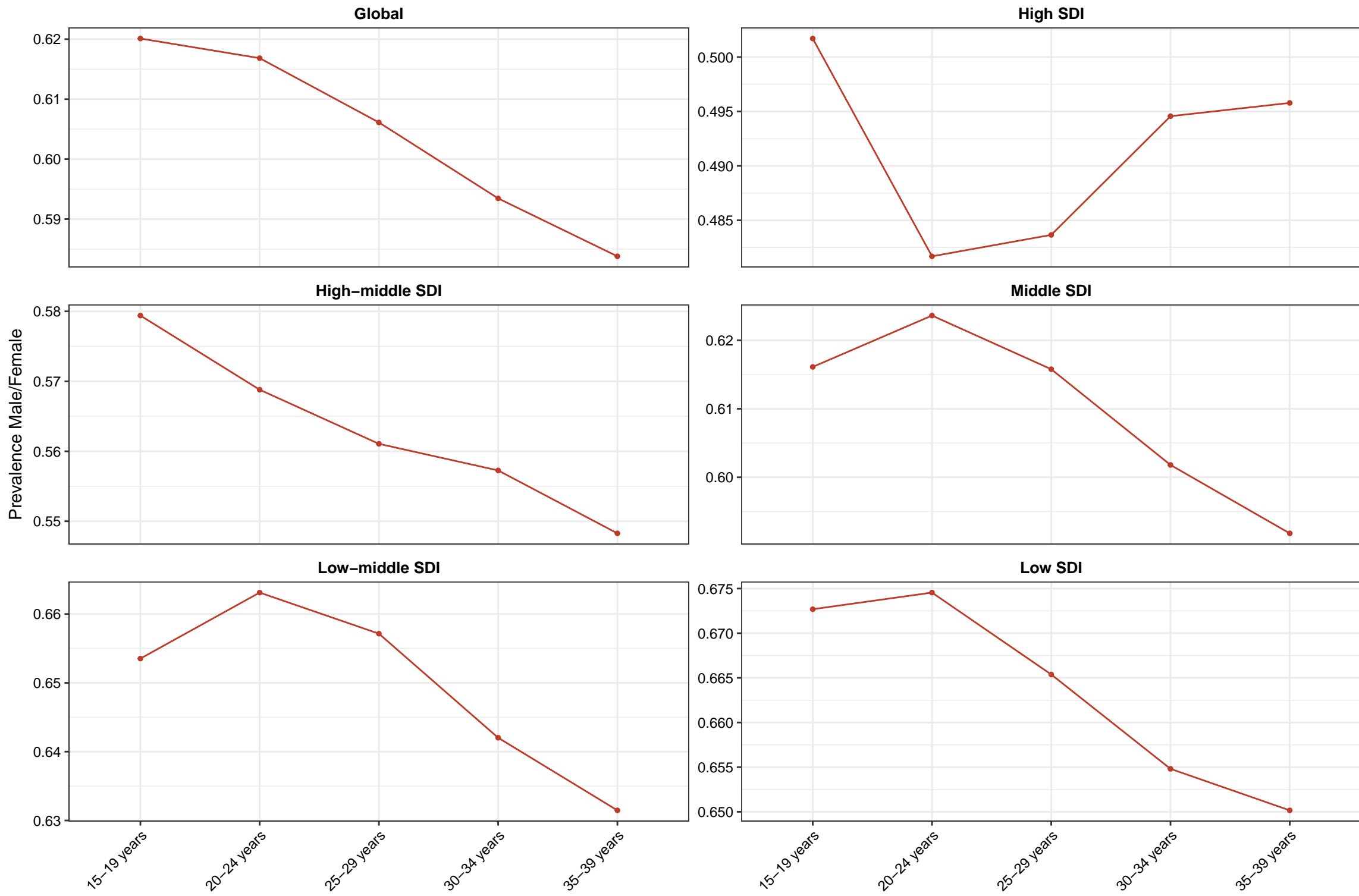

Supplement: Supplementary file 1 — Supplementary Material 1: Fig. S1. Ratio of male to female prevalence of migraine in different age subgroups. [file 10194_2024_1832_MOESM1_ESM.pdf]

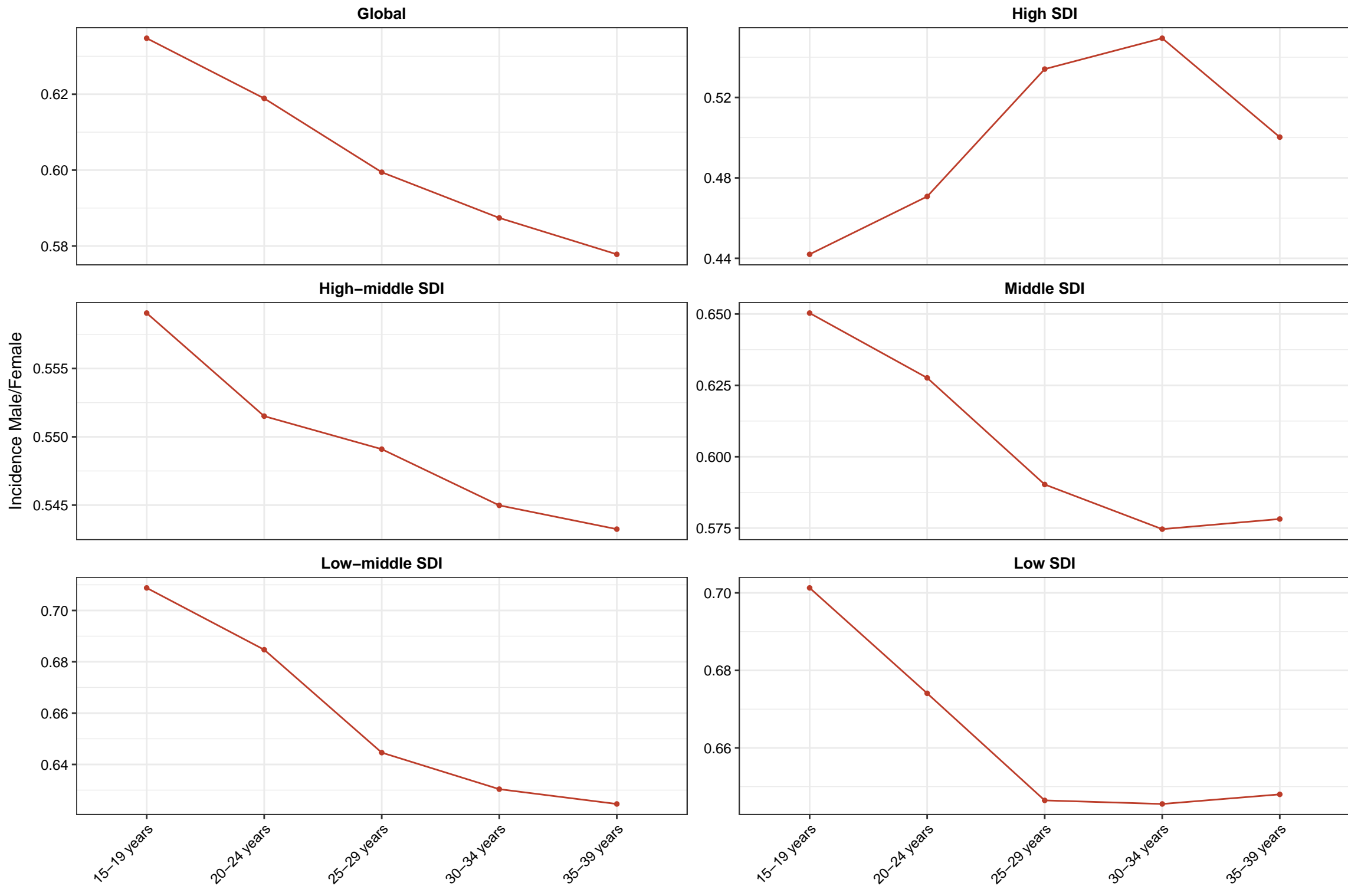

Supplement: Supplementary file 2 — Supplementary Material 2: Fig. S2. Ratio of male to female incidence of migraine in different age subgroups. [file 10194_2024_1832_MOESM2_ESM.pdf]

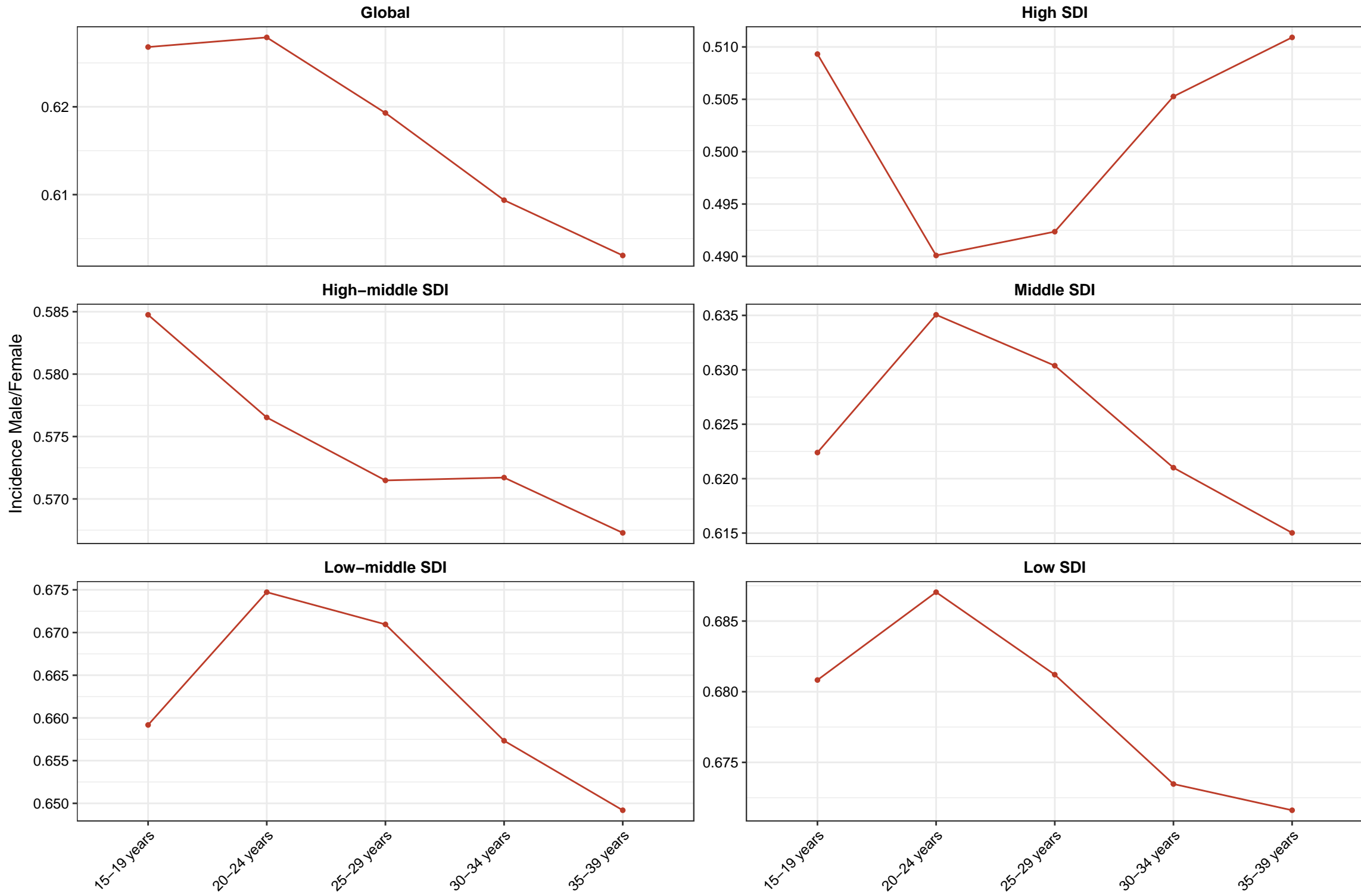

Supplement: Supplementary file 3 — Supplementary Material 3: Fig. S3 Ratio of male to female DALYs of migraine in different age subgroups. [file 10194_2024_1832_MOESM3_ESM.pdf]

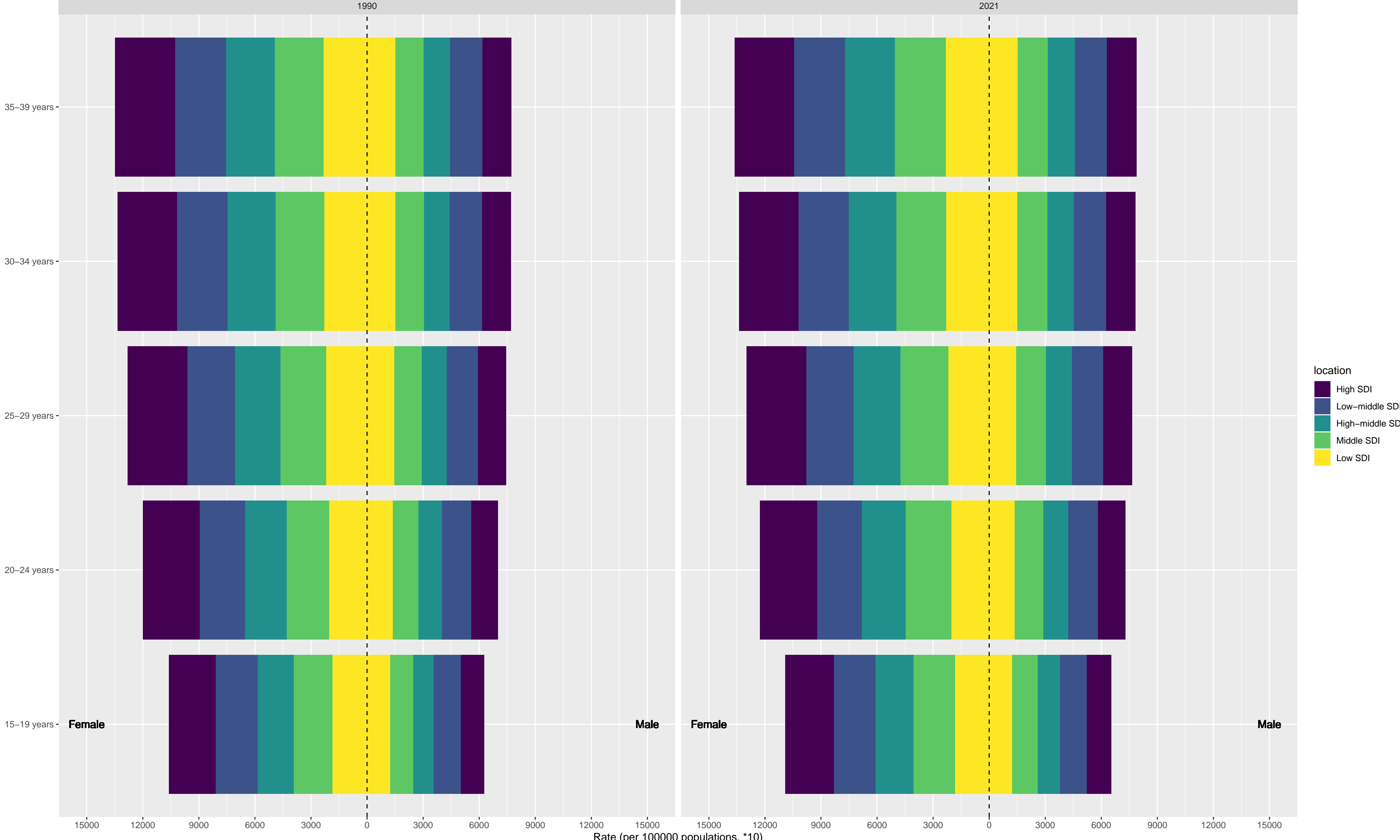

Supplement: Supplementary file 4 — Supplementary Material 4: Fig. S4 Prevalence rate of migraine in males and females across different age groups in five SDI regions from 1990 to 2021 [file 10194_2024_1832_MOESM4_ESM.pdf]

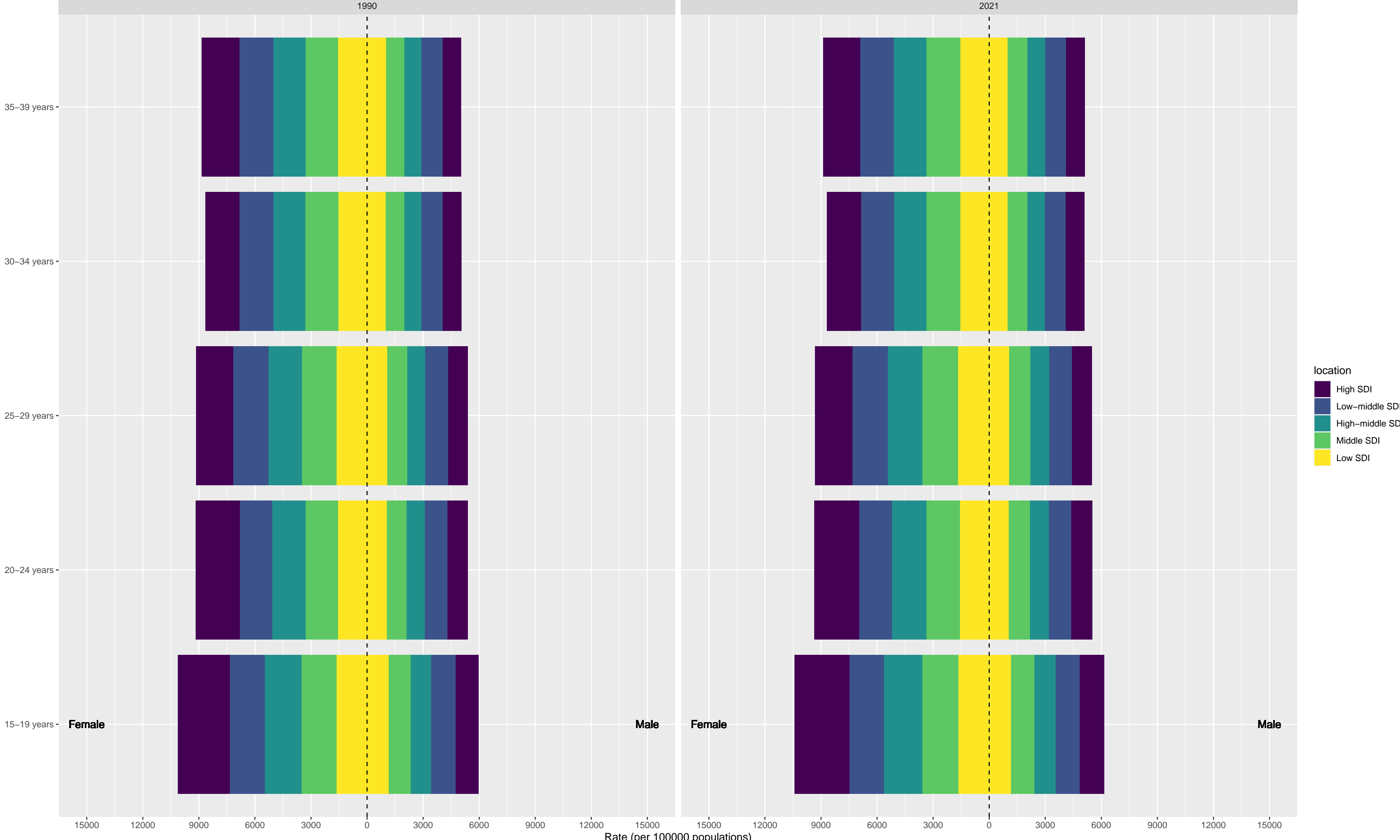

Supplement: Supplementary file 5 — Supplementary Material 5: Fig. S5 Incidence rate of migraine in males and females across different age groups in five SDI regions from 1990 to 2021 [file 10194_2024_1832_MOESM5_ESM.pdf]

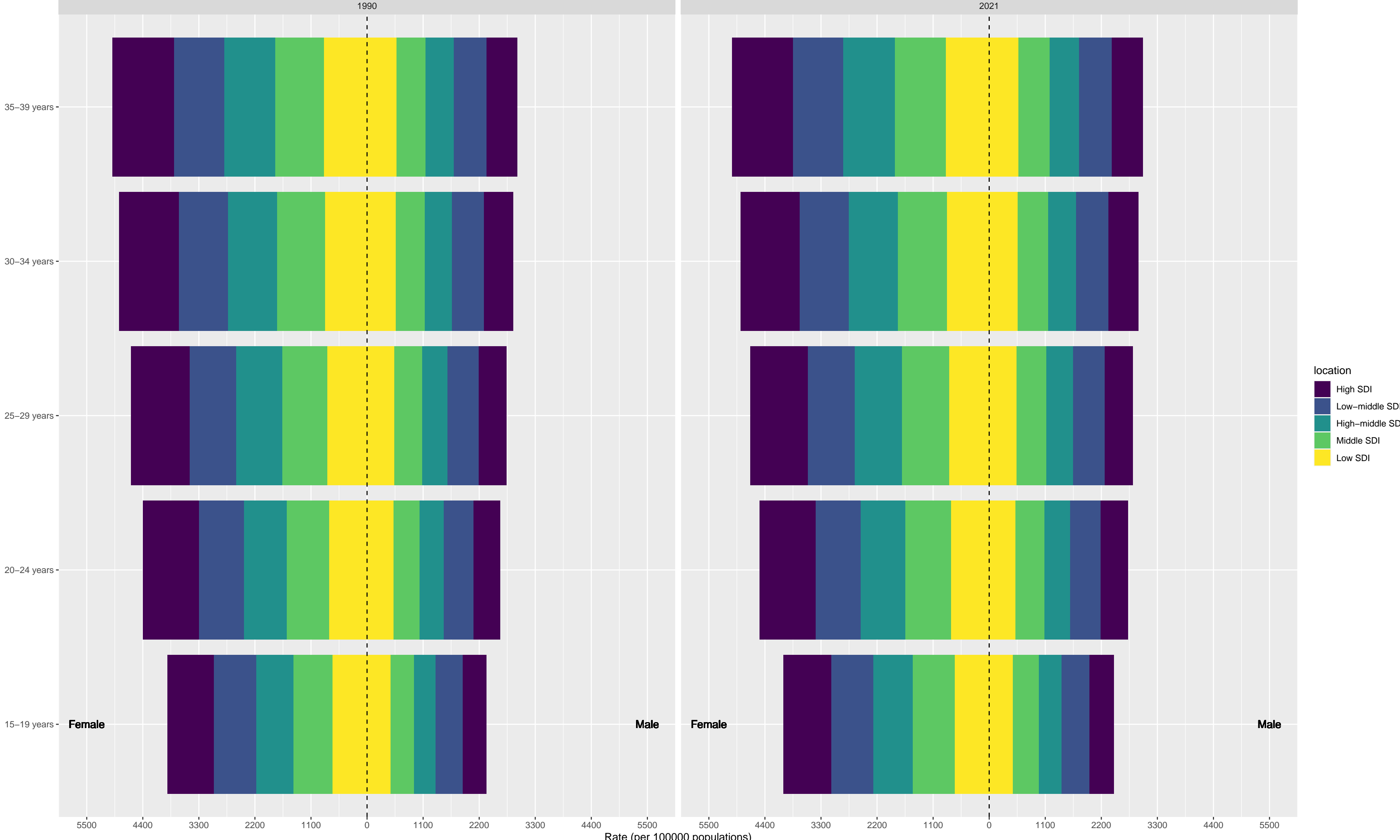

Supplement: Supplementary file 6 — Supplementary Material 6: Fig. S6 DALYs rate of migraine in males and females across different age groups in five SDI regions from 1990 to 2021 [file 10194_2024_1832_MOESM6_ESM.pdf]

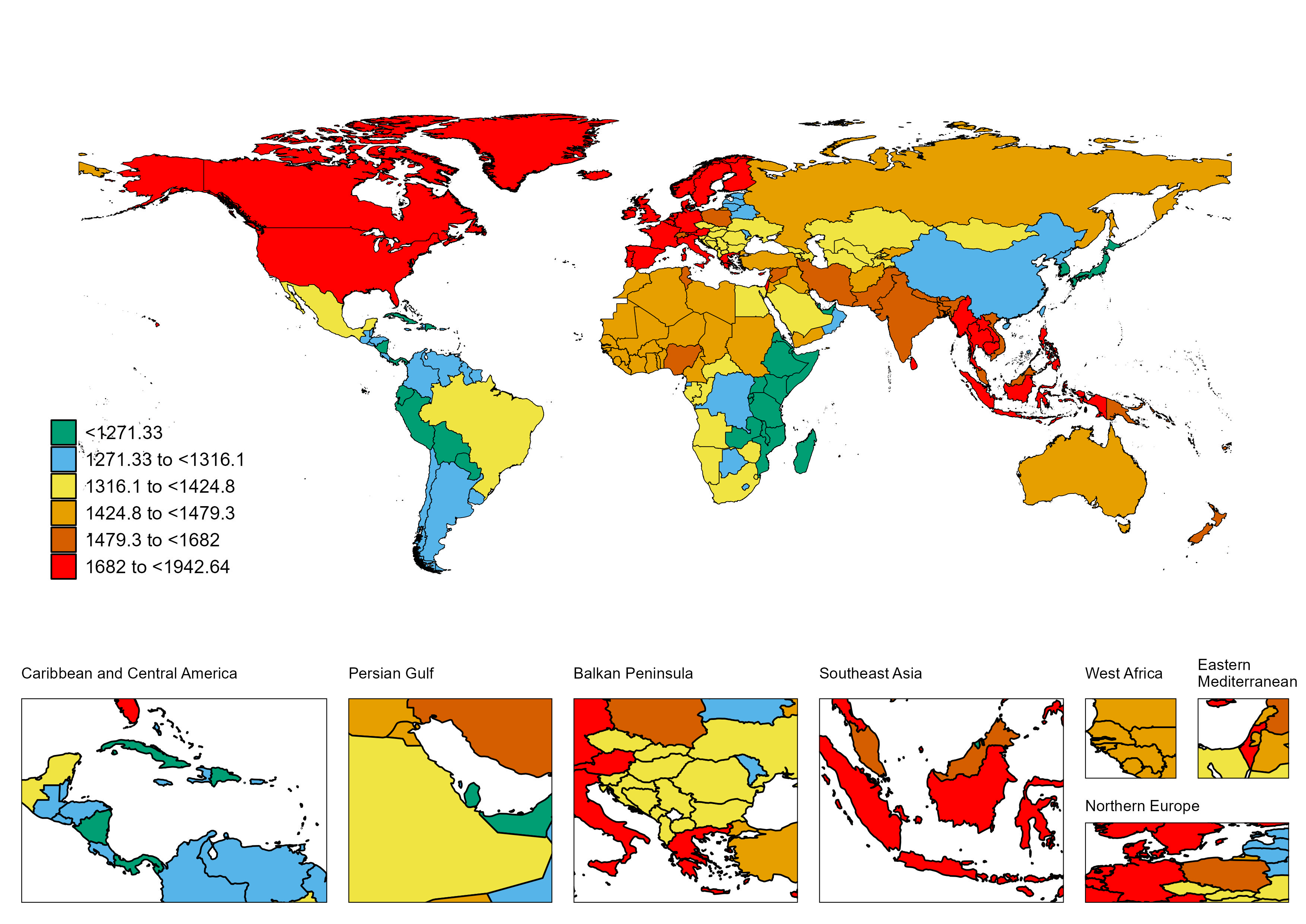

Supplement: Supplementary file 7 — Supplementary Material 7: Fig. S7：The global disease burden of migraine incidence rate for both sexes in 204 countries and territories. [file 10194_2024_1832_MOESM7_ESM.jpg]

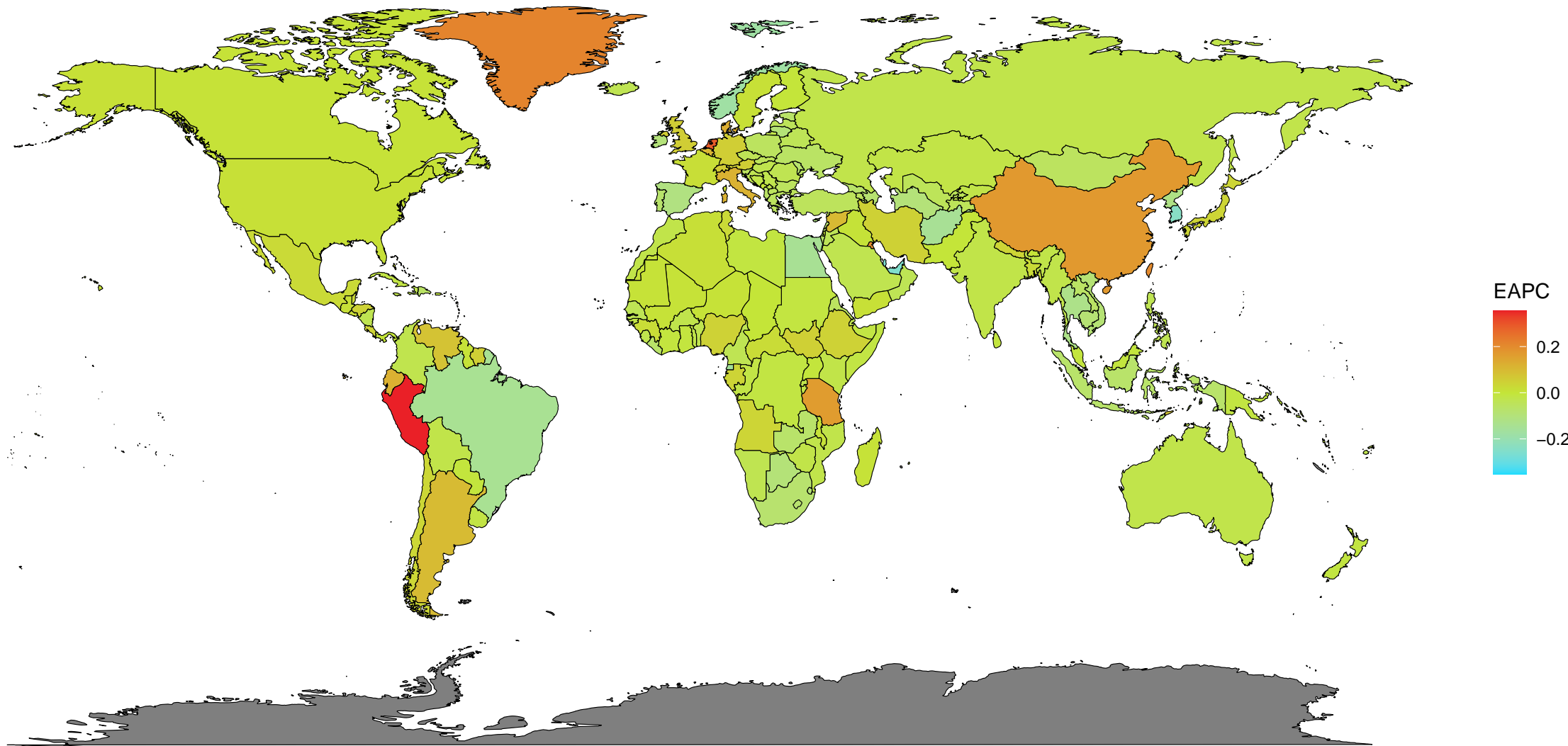

Supplement: Supplementary file 8 — Supplementary Material 8: Fig. S8：EAPC for migraine incidence [file 10194_2024_1832_MOESM8_ESM.pdf]

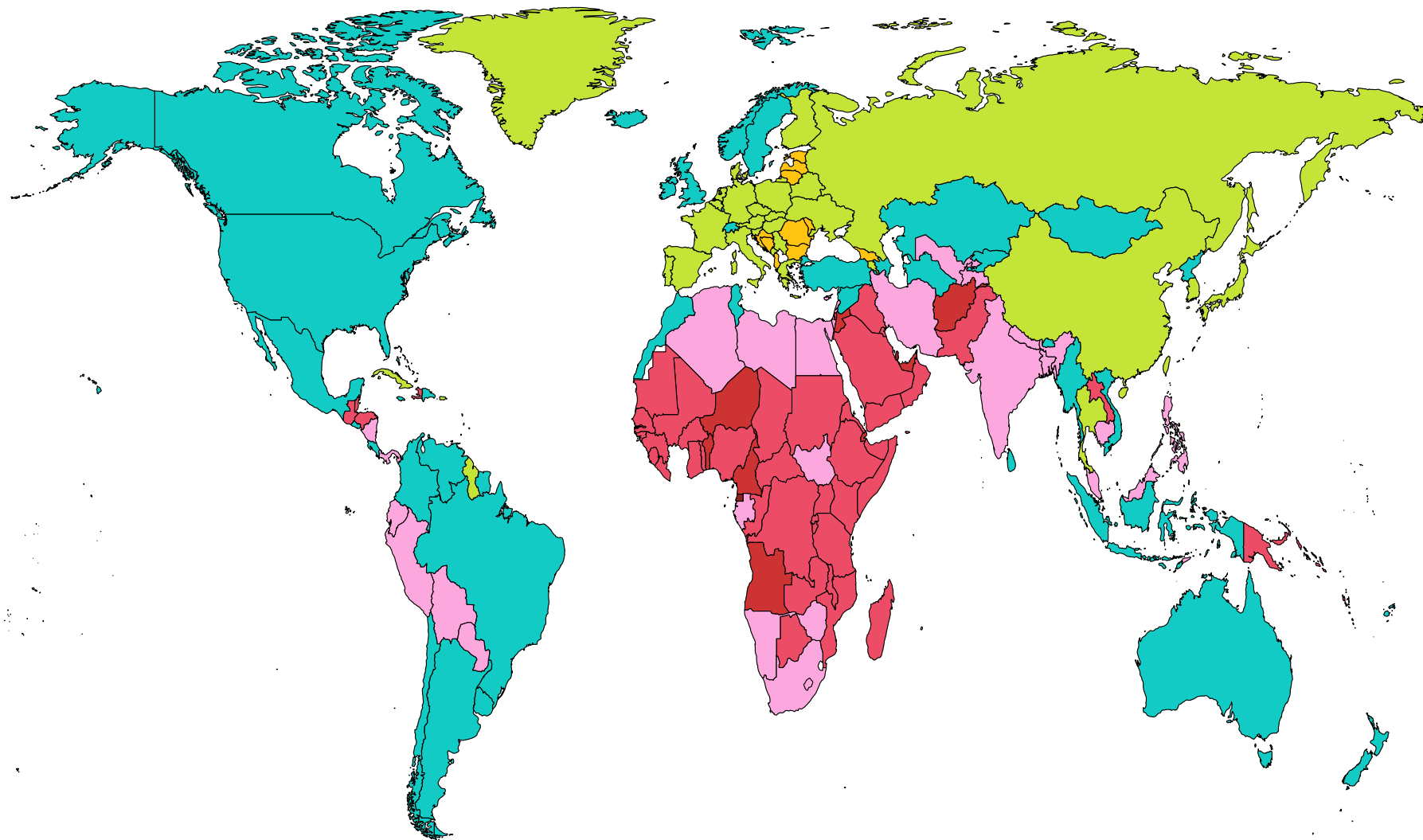

### Change in cases

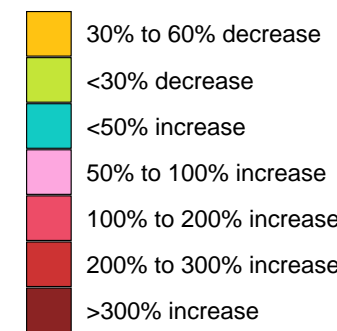

Supplement: Supplementary file 9 — Supplementary Material 9: Fig. S9: Change incidence cases of migraine for both sexes in 204 countries and territories. [file 10194_2024_1832_MOESM9_ESM.pdf]

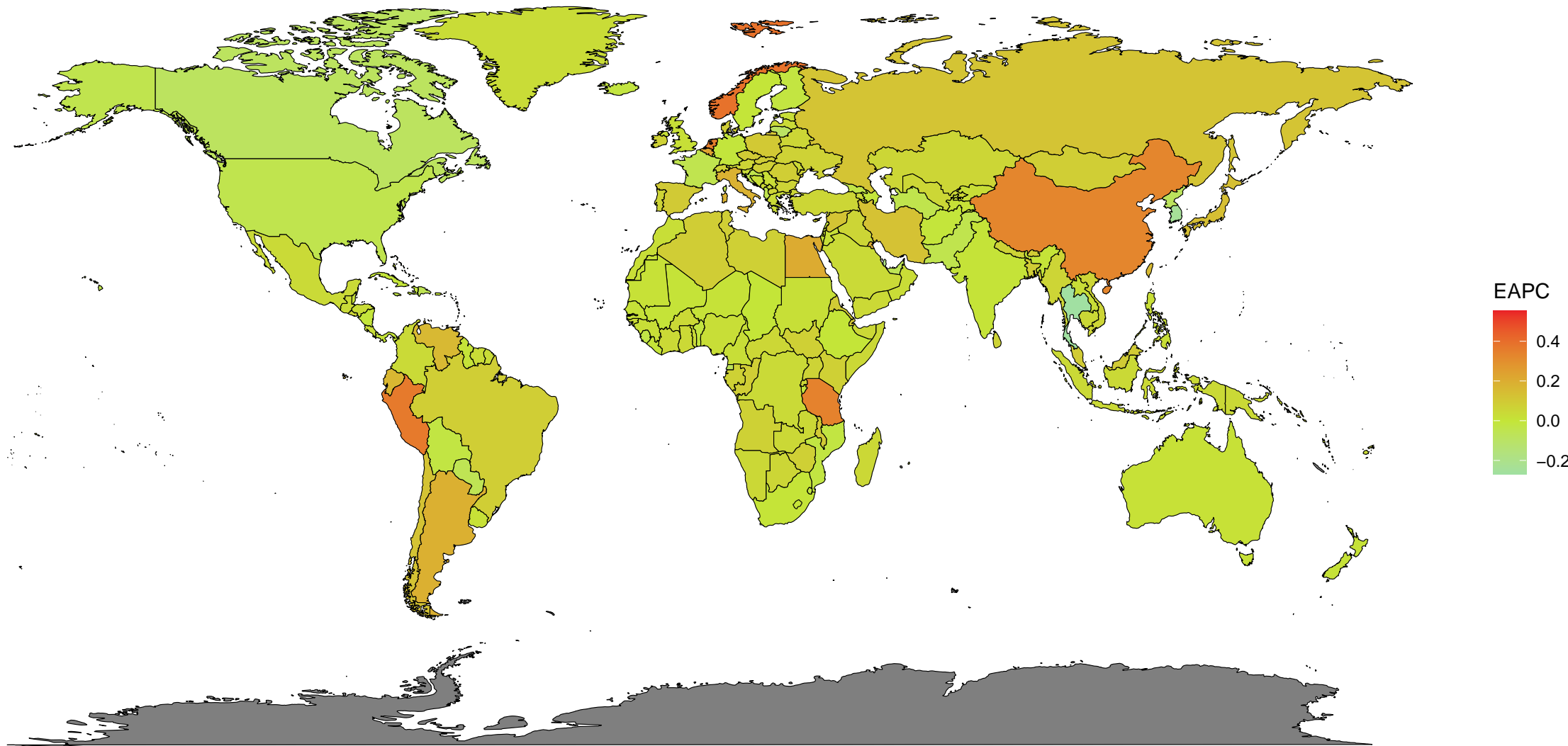

Supplement: Supplementary file 11 — Supplementary Material 11: Fig. S11：EAPC for migraine DALYs [file 10194_2024_1832_MOESM11_ESM.pdf]

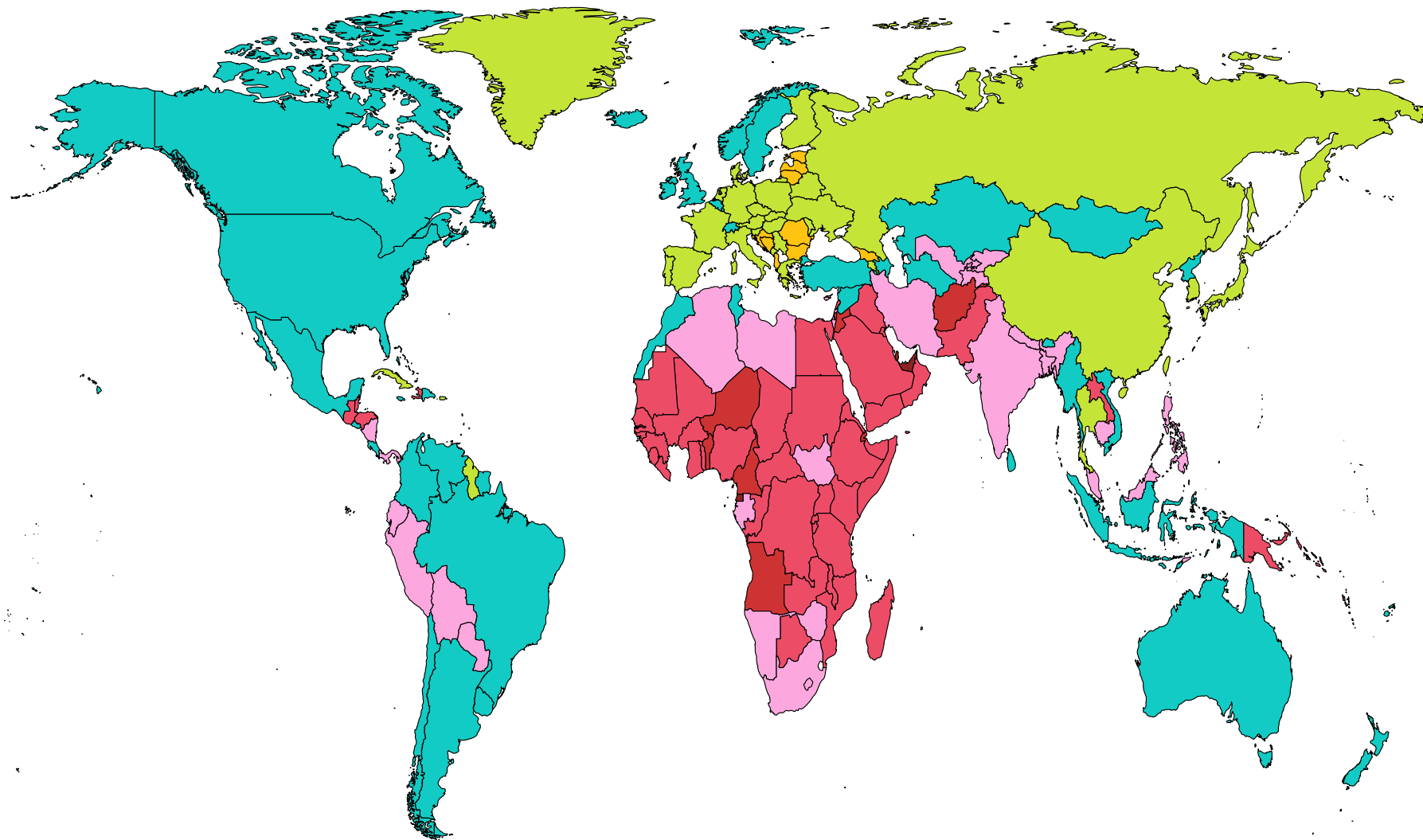

### Change in cases

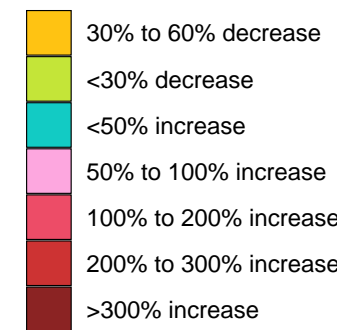

Supplement: Supplementary file 12 — Supplementary Material 12: Fig. S12: Change DALYs cases of migraine for both sexes in 204 countries and territories. [file 10194_2024_1832_MOESM12_ESM.pdf]

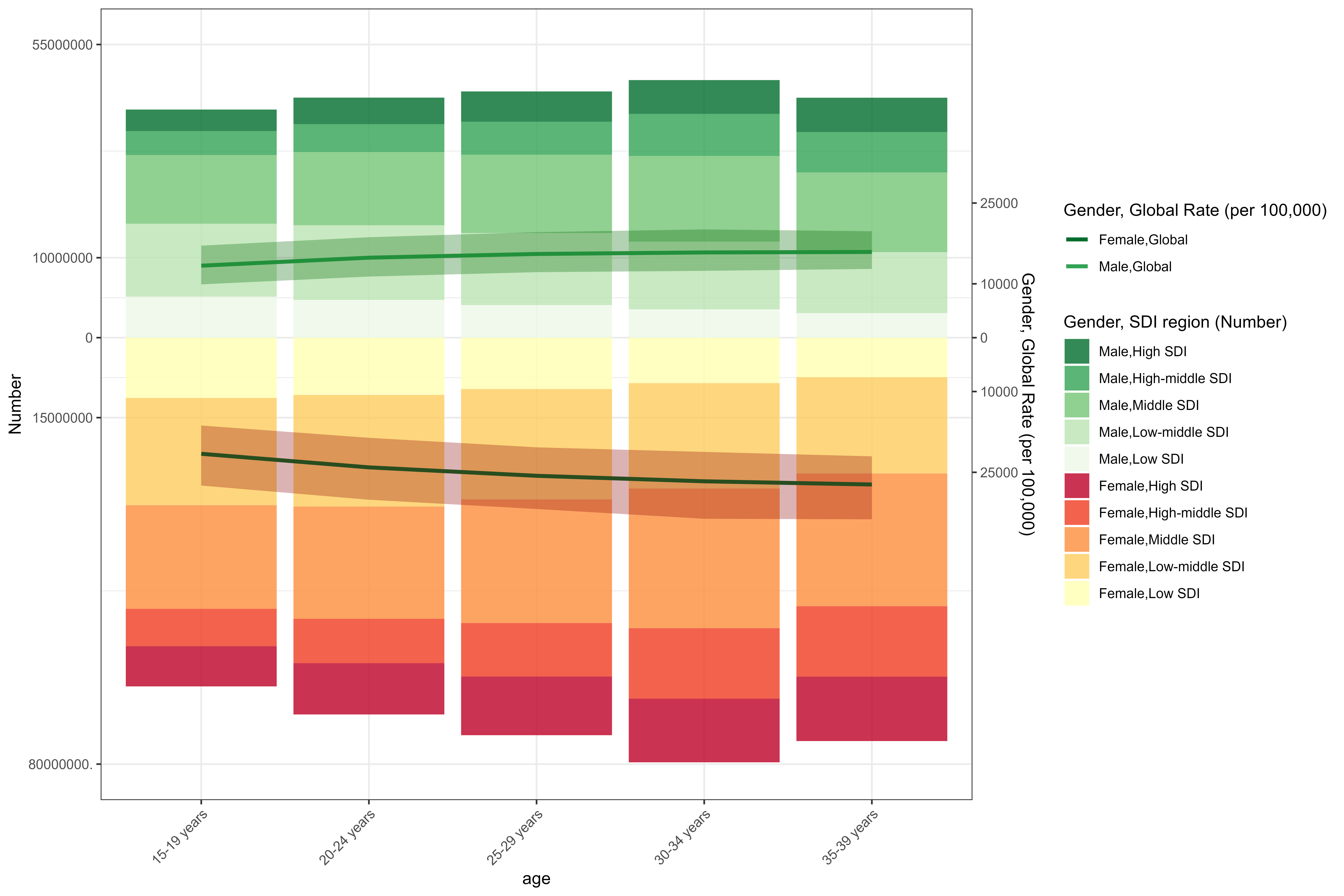

Supplement: Supplementary file 13 — Supplementary Material 13: Fig. S13: Prevalence and prevalence rates by age for men and women worldwide and in 5 SDI regions, 2021 [file 10194_2024_1832_MOESM13_ESM.jpg]

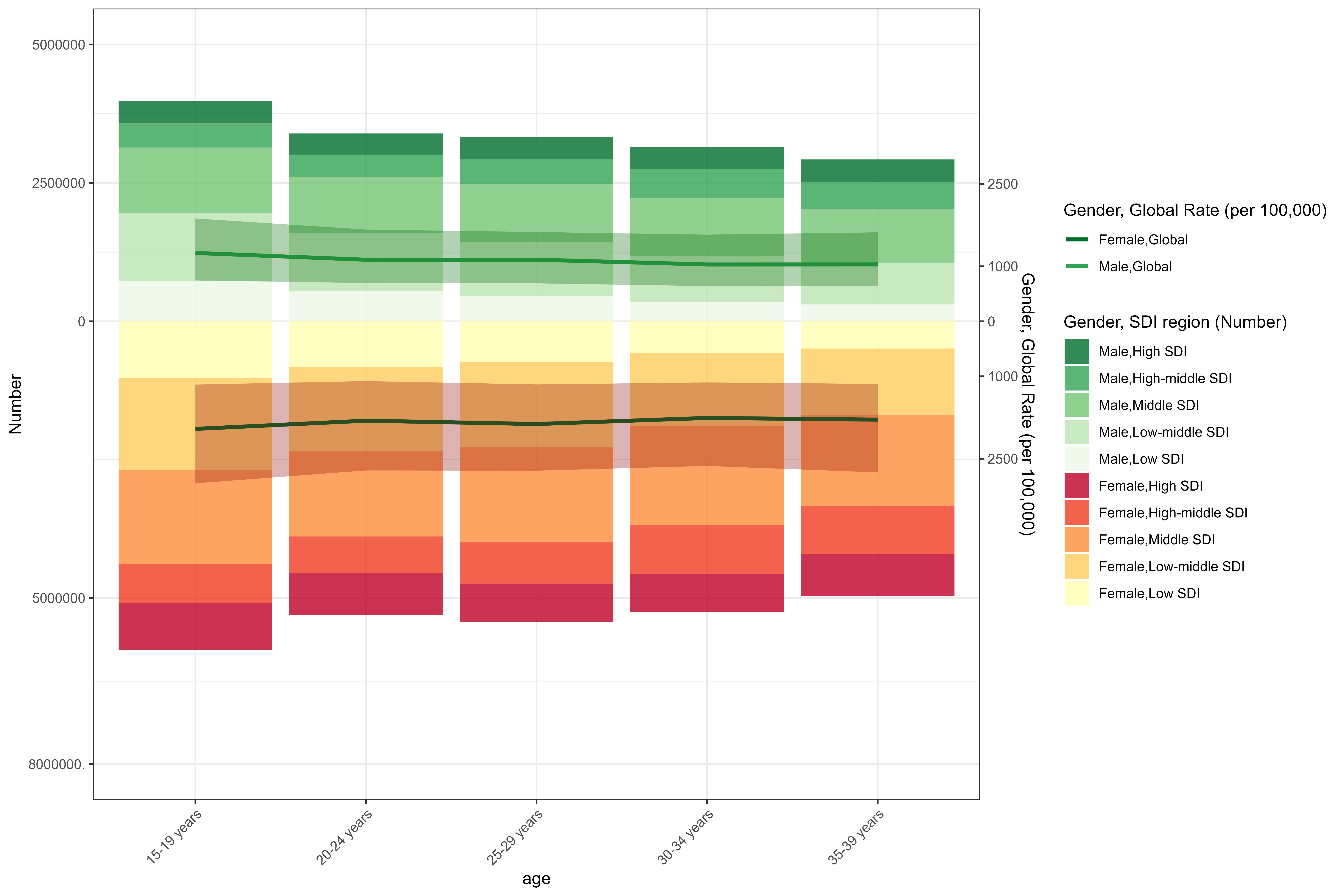

Supplement: Supplementary file 14 — Supplementary Material 14: Fig. S14: Incidence and incidence rates by age for men and women worldwide and in 5 SDI regions, 2021 [file 10194_2024_1832_MOESM14_ESM.jpg]

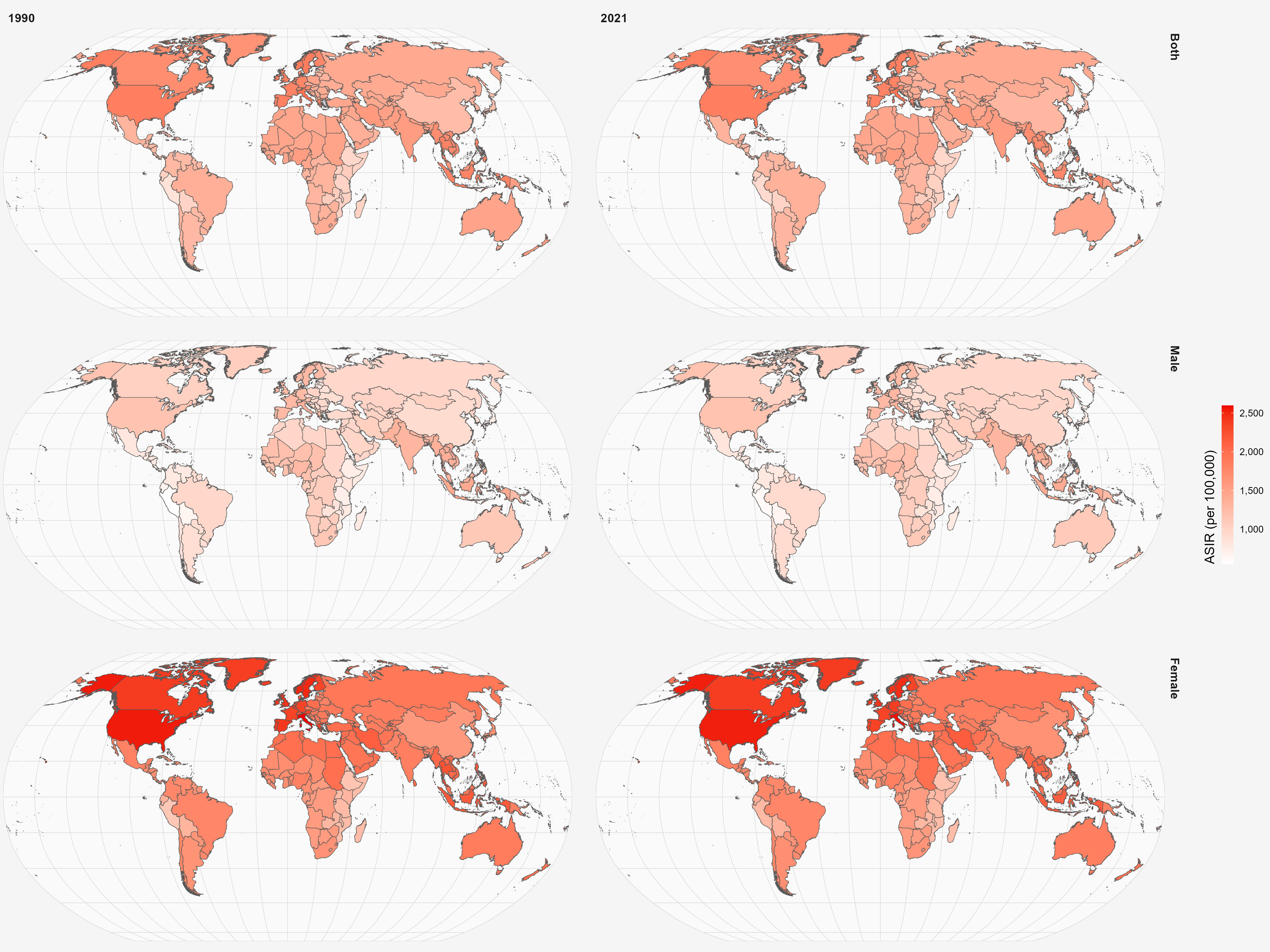

Supplement: Supplementary file 15 — Supplementary Material 15: Fig. S15 Comparison of the global disease burden of migraine incidence in males and females across 204 countries and territories between 1990 and 2021 [file 10194_2024_1832_MOESM15_ESM.jpg]

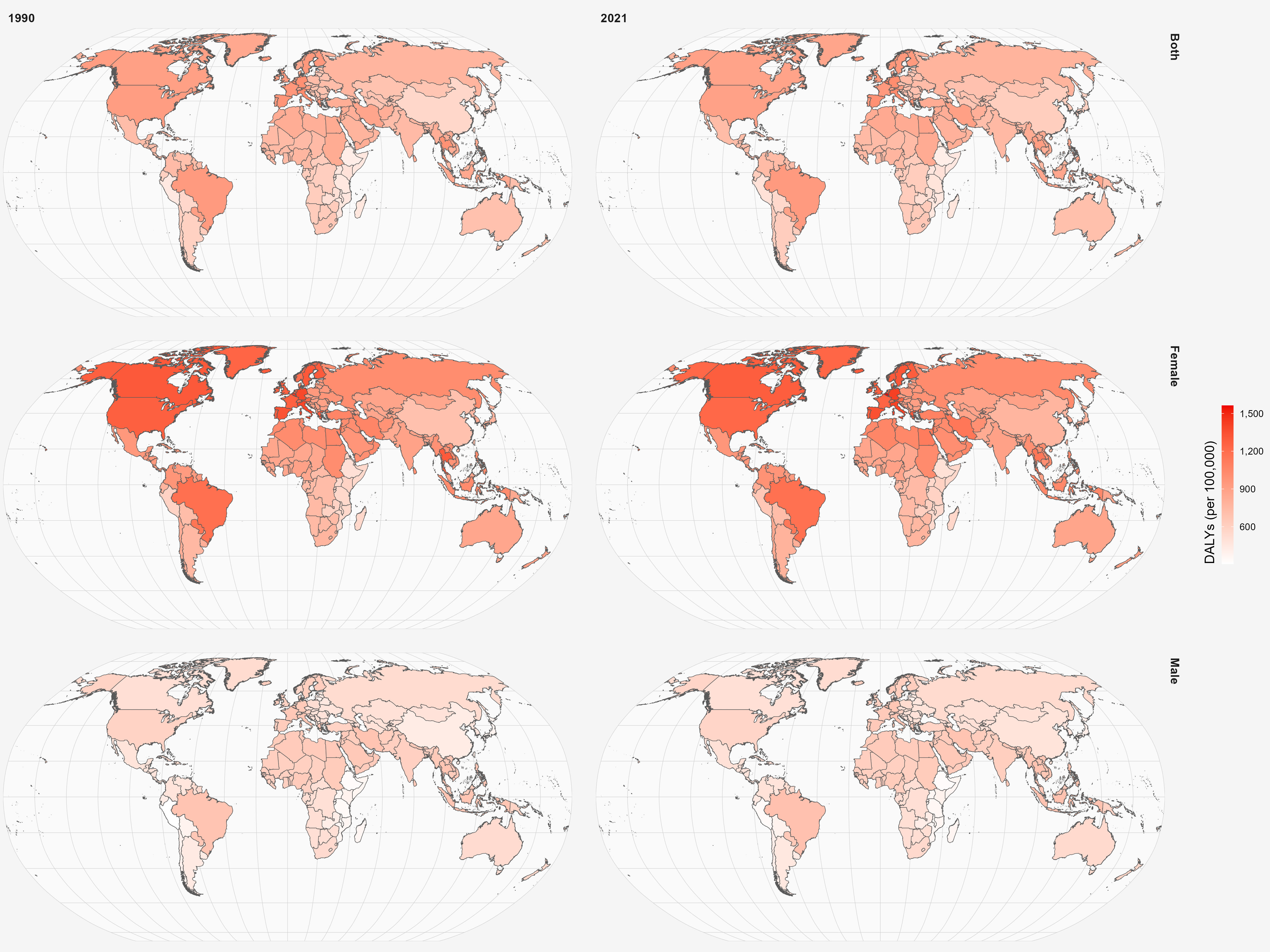

Supplement: Supplementary file 16 — Supplementary Material 16: Fig. S16 Comparison of the global disease burden of migraine DALYs in males and females across 204 countries and territories between 1990 and 2021 [file 10194_2024_1832_MOESM16_ESM.jpg]

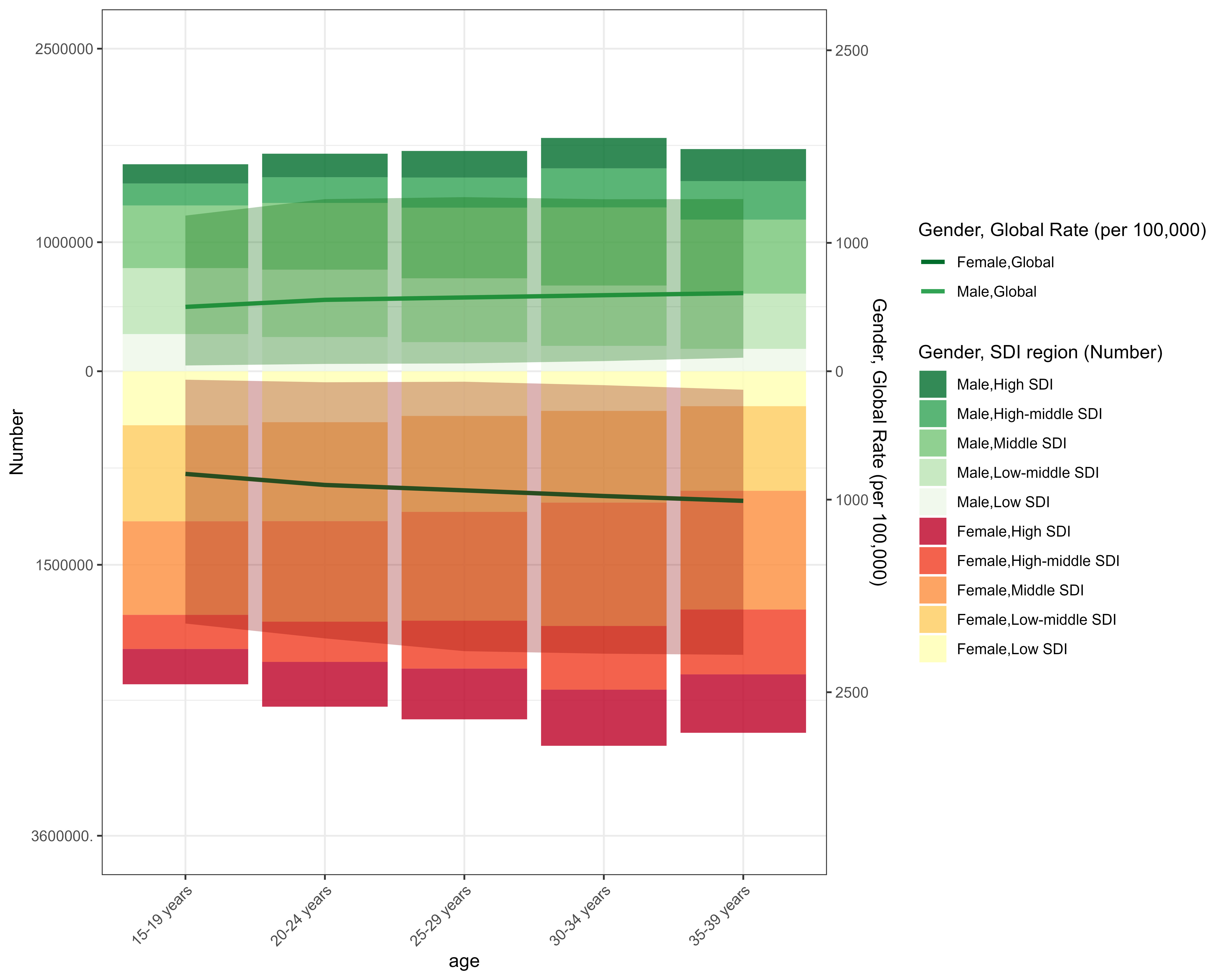

Supplement: Supplementary file 17 — Supplementary Material 17: Fig. S17: DALYs and DALYs rates by age for men and women worldwide and in 5 SDI regions, 2021 [file 10194_2024_1832_MOESM17_ESM.jpg]

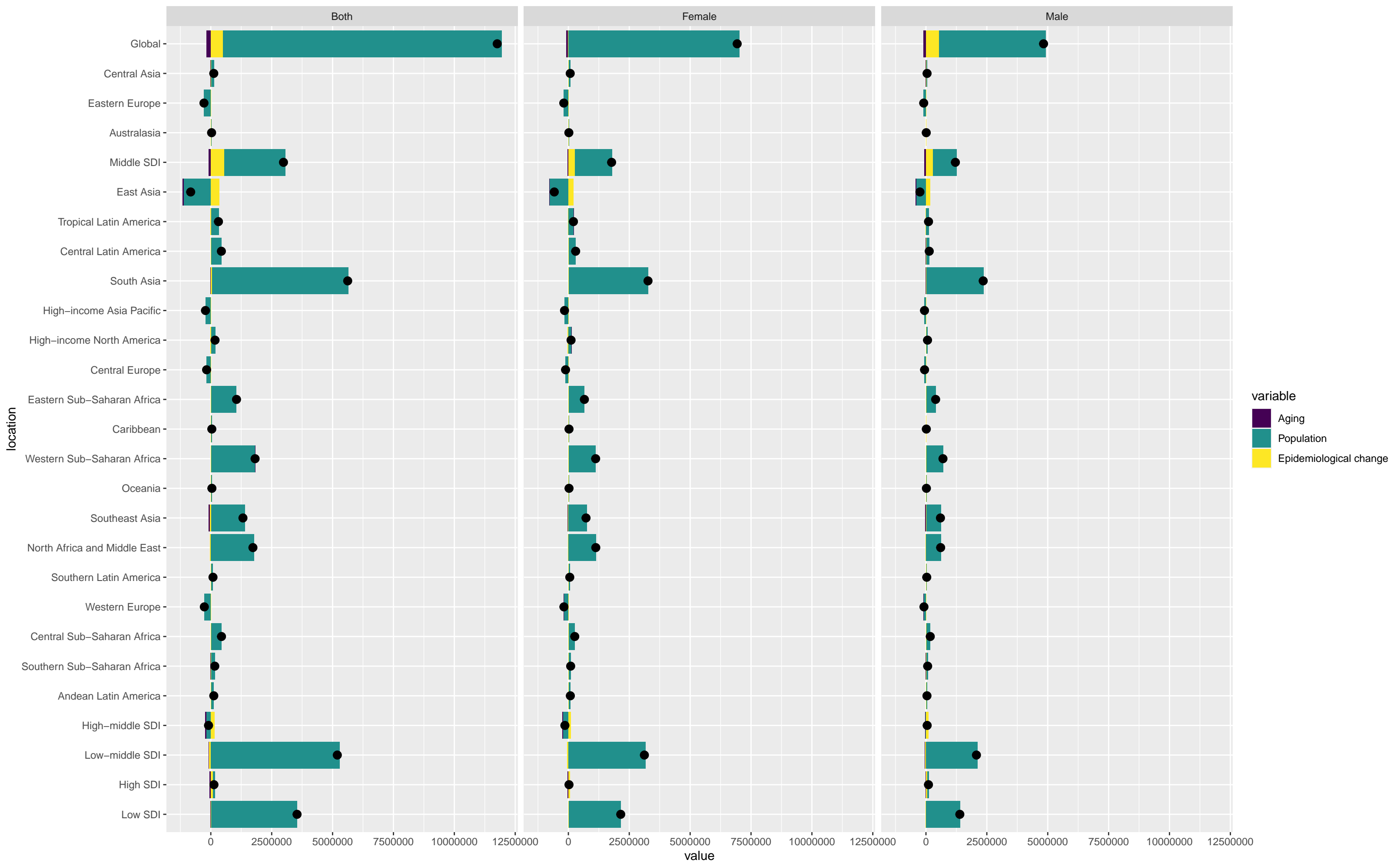

Supplement: Supplementary file 18 — Supplementary Material 18: Fig. S18: Decomposition analysis of migraine change in incidence by SDI and 21 GBD region, 1990 to 2021. [file 10194_2024_1832_MOESM18_ESM.pdf]

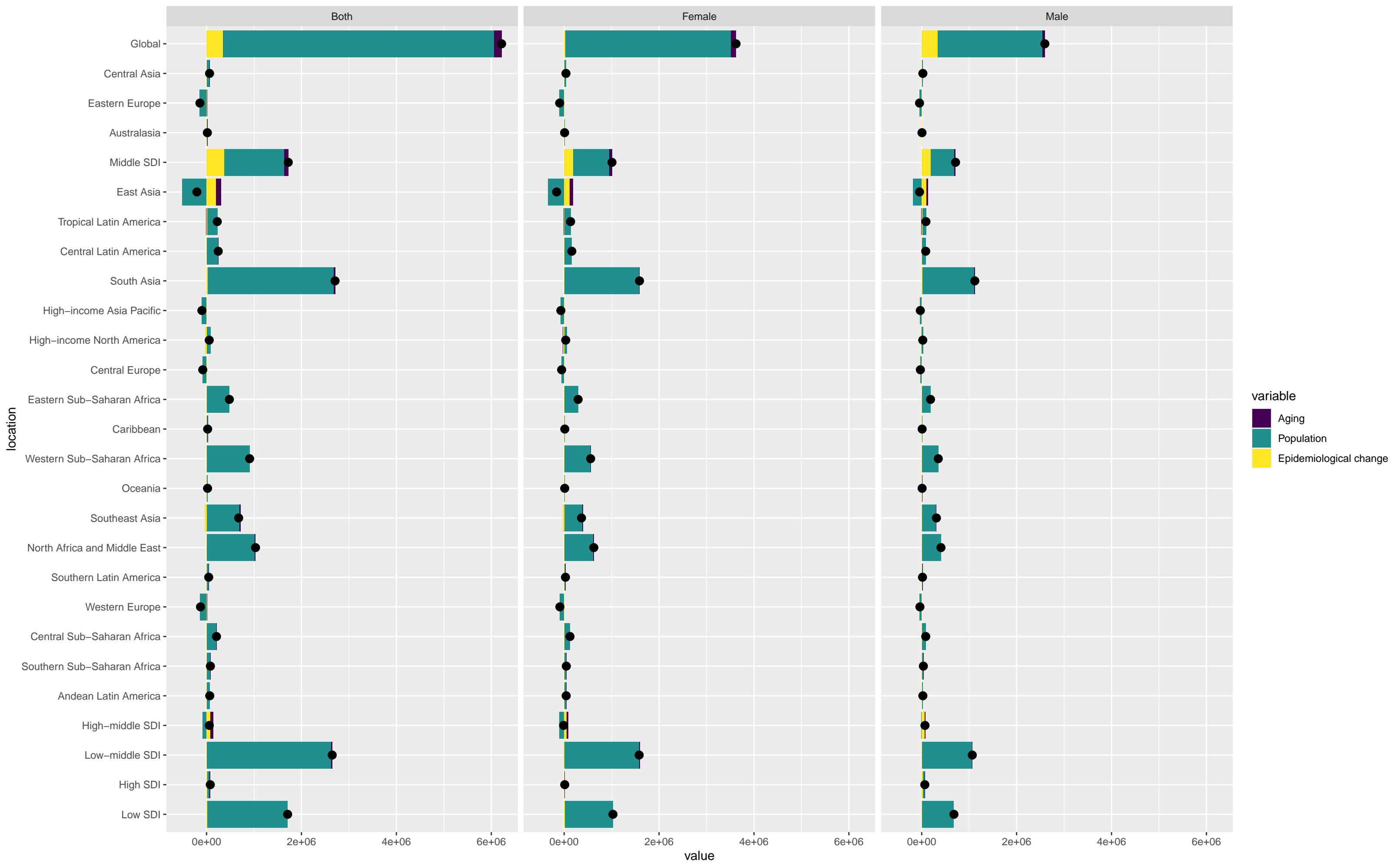

Supplement: Supplementary file 19 — Supplementary Material 19: Fig.S19: Decomposition analysis of migraine change in DALYs by SDI and 21 GBD region, 1990 to 2021. [file 10194_2024_1832_MOESM19_ESM.pdf]

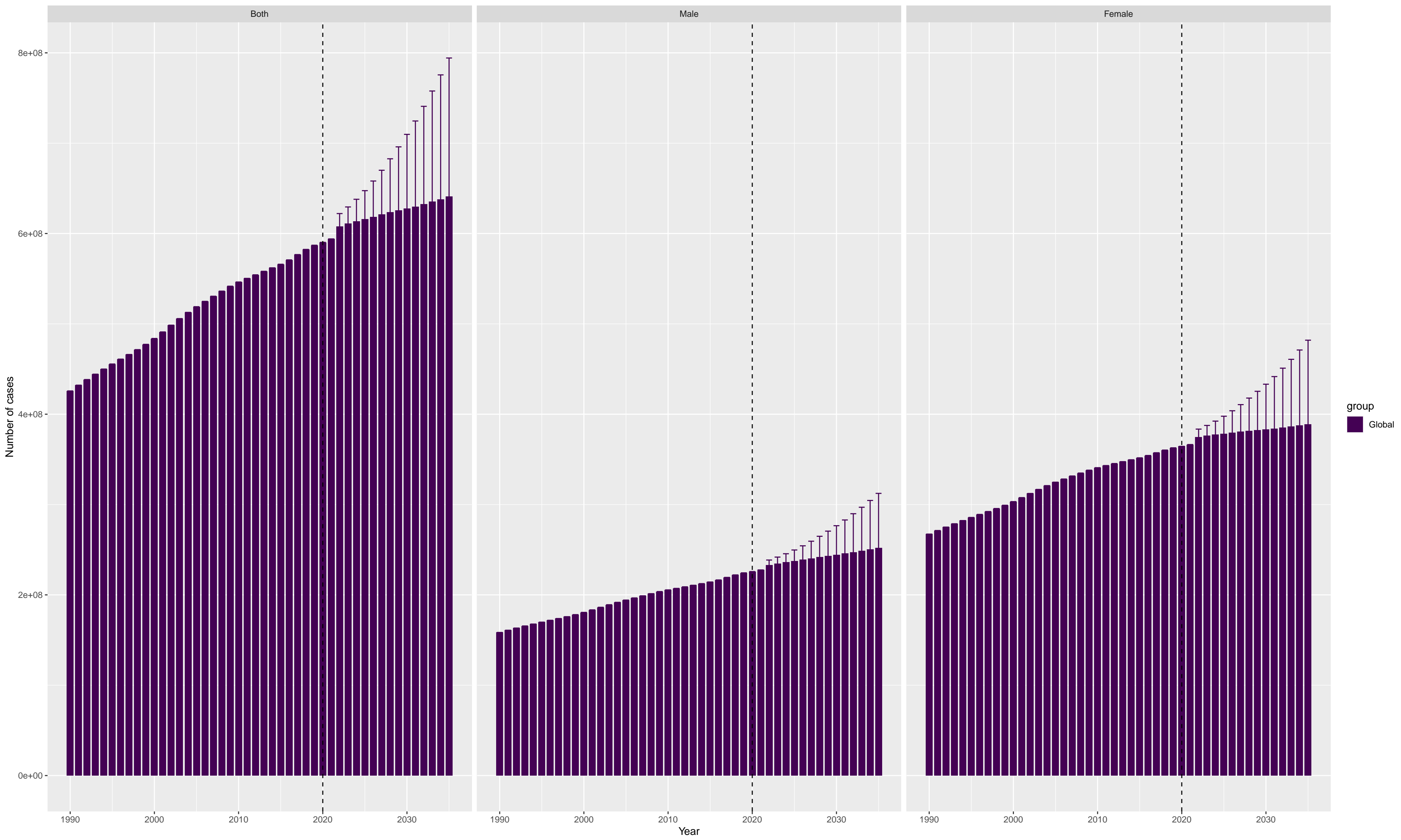

Supplement: Supplementary file 20 — Supplementary Material 20: Fig. S20: Future forecasts of GBD in migraine prevalence. [file 10194_2024_1832_MOESM20_ESM.pdf]

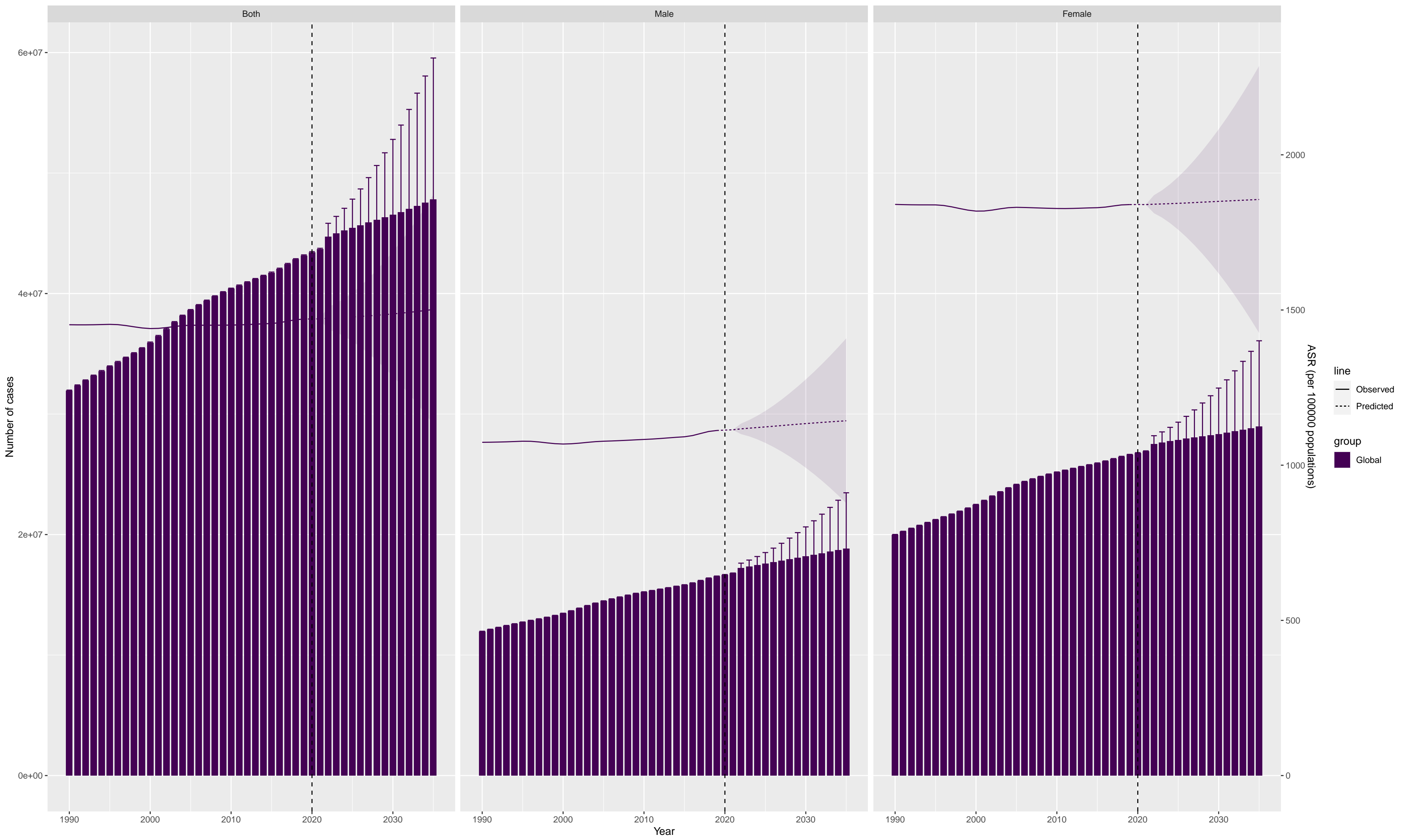

Supplement: Supplementary file 21 — Supplementary Material 21: Fig. S21: Future forecasts of GBD in migraine incidence. [file 10194_2024_1832_MOESM21_ESM.pdf]

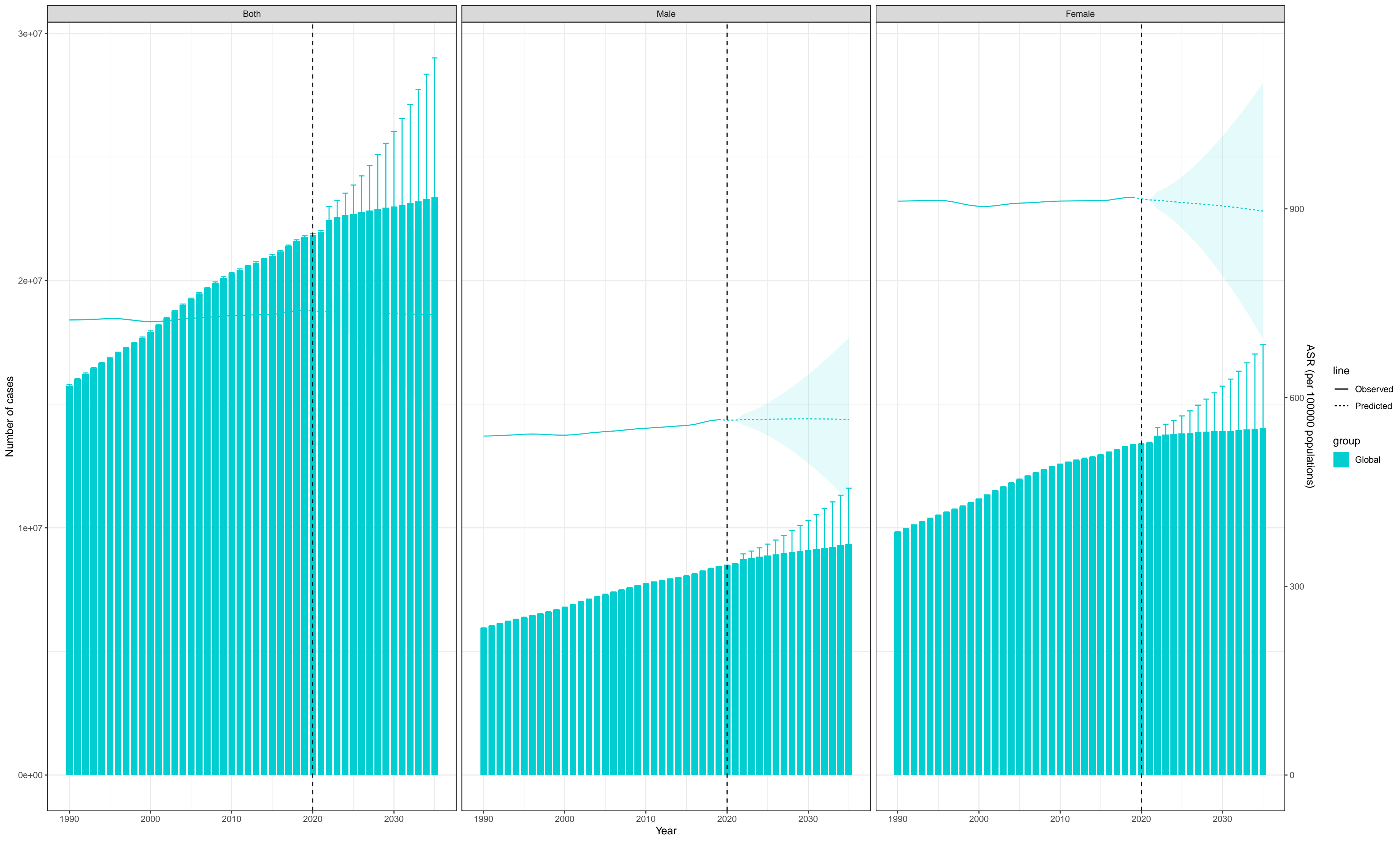

Supplement: Supplementary file 22 — Supplementary Material 22: Fig. S22: Future forecasts of GBD in migraine DALYs. [file 10194_2024_1832_MOESM22_ESM.pdf]
