# Supplementary material for: Global, regional, and national burden and trends of migraine among youths and young adults aged 15–39 years from 1990 to 2021: findings from the global burden of disease study 2021
Source: J Headache Pain. 2024 Aug 12;25(1):131. doi: 10.1186/s10194-024-01832-0 (PMC11318134; doi:10.1186/s10194-024-01832-0)
Supplement: Supplementary file 10 — Supplementary Material 10: Fig. S10：The global disease burden of migraine DALYs rate for both sexes in 204 countries and territories. [file 10194_2024_1832_MOESM10_ESM.pdf]

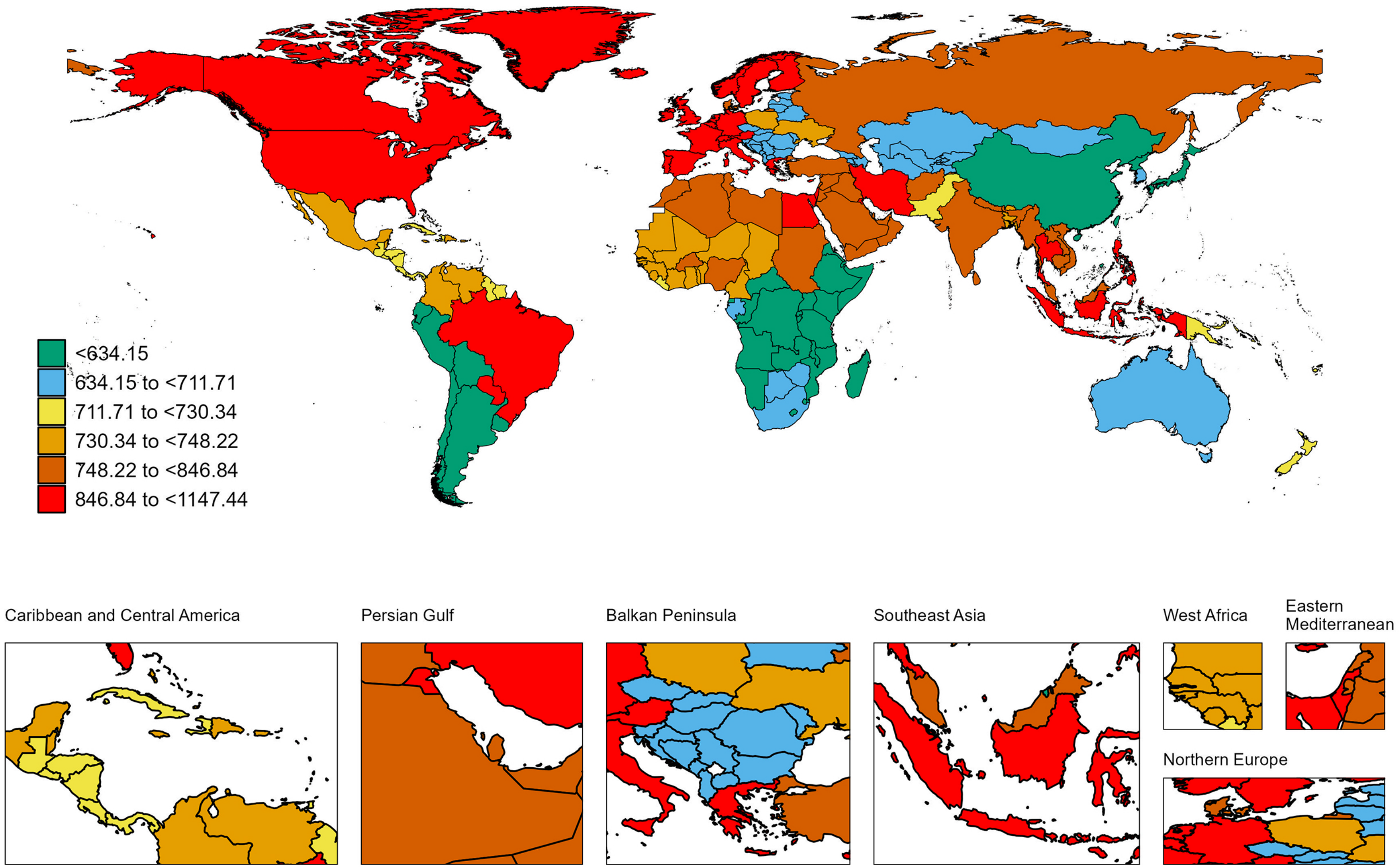

<634.15
634.15 to <711.71
711.71 to <730.34
730.34 to <748.22
748.22 to <846.84
846.84 to <1147.44
Caribbean and Central America
Persian Gulf
Balkan Peninsula
Southeast Asia
West Africa
Eastern Mediterranean
Northern Europe
