# Supplementary material for: Global, regional, and national burden and trends of migraine among youths and young adults aged 15–39 years from 1990 to 2021: findings from the global burden of disease study 2021
Source: J Headache Pain. 2024 Aug 12;25(1):131. doi: 10.1186/s10194-024-01832-0 (PMC11318134; doi:10.1186/s10194-024-01832-0)
Supplement: Supplementary file 23 — Supplementary Material 23: Table S1 Incidence of Migraine Between 1990 and 2021 in 15 to 39 years at the Global and Regional Level [file 10194_2024_1832_MOESM23_ESM.docx]

| **TableS1 Incidence of Migraine Between 1990 and 2021 in 15 to 39 years at the Global and Regional Level** | | | | | |
| --- | --- | --- | --- | --- | --- |
| **Location** | **1990** | | **2021** | | **EAPC_95%CI** |
|  | **Num**ber(95%UI) | **ASR**(95%UI) | **Num**ber(95%UI) | **ASR**(95%UI) |  |
| Global | 31939565.5 (26222391.9-39538607.4) | 1457.2 (1196.4-1803.9) | 43699829.6 (35822349.9-54100215.9) | 1469 (1204.2-1818.6) | 0.03 (0.01-0.04) |
| High SDI | 5599422.7 (4602974.7-6835354.5) | 1613.8 (1326.6-1970) | 5728498.6 (4740713.5-7094533.9) | 1621.7 (1342.1-2008.4) | 0.02 (0-0.03) |
| High-middle SDI | 6291918.1 (5180815.2-7750948.5) | 1390.4 (1144.8-1712.8) | 6198706.3 (5093839.3-7646037.5) | 1407.9 (1157-1736.7) | 0.05 (0.04-0.07) |
| Middle SDI | 10659194.5 (8754891.2-13144672.5) | 1416.3 (1163.2-1746.5) | 13643395.8 (11251240.6-16821849.5) | 1471 (1213.1-1813.7) | 0.13 (0.11-0.15) |
| Low-middle SDI | 6892018.8 (5619309.8-8549895.2) | 1520.1 (1239.4-1885.7) | 12086731.1 (9919347-15022217.5) | 1506.1 (1236.1-1871.9) | -0.05 (-0.07--0.03) |
| Low SDI | 2468330.5 (1997301-3109188.2) | 1339.2 (1083.7-1686.9) | 6009649.6 (4856729.2-7581612.2) | 1338.3 (1081.5-1688.3) | 0 (-0.02-0.01) |
| Andean Latin America | 141843.7 (112093.5-183432.9) | 917.3 (724.9-1186.2) | 264353.9 (205715.5-348971.6) | 976.2 (759.7-1288.7) | 0.23 (0.17-0.28) |
| Australasia | 122390.8 (99492-156162.2) | 1501 (1220.2-1915.2) | 155101 (125754.4-196444) | 1481.3 (1201-1876.1) | -0.02 (-0.03--0.01) |
| Caribbean | 189954.9 (147668.8-245281) | 1277.9 (993.4-1650.1) | 231466.2 (182015.5-302387.3) | 1271.6 (999.9-1661.2) | -0.02 (-0.02--0.02) |
| Central Asia | 408322.1 (322016-523294.5) | 1435 (1131.7-1839.1) | 530362.4 (422949.6-678643.6) | 1418.6 (1131.3-1815.2) | -0.04 (-0.06--0.03) |
| Central Europe | 673145.9 (548147.6-851918.2) | 1436.9 (1170-1818.5) | 500658.4 (403615.3-630211.4) | 1429.6 (1152.5-1799.6) | -0.03 (-0.04--0.03) |
| Central Latin America | 888362.1 (710884.8-1129508.1) | 1301.3 (1041.3-1654.5) | 1322475 (1073473.7-1676931.2) | 1307.3 (1061.1-1657.7) | 0.01 (0.01-0.02) |
| Central Sub-Saharan Africa | 273996 (213946.7-356999) | 1319.7 (1030.5-1719.5) | 713008.8 (558803.2-929272.9) | 1318 (1033-1717.8) | 0 (0-0) |
| East Asia | 7046265.8 (5771226.1-8634688.4) | 1245.6 (1020.2-1526.4) | 6219830.8 (5103640.7-7556310.6) | 1298.4 (1065.4-1577.4) | 0.17 (0.13-0.21) |
| Eastern Europe | 1211323.4 (998063.6-1483687.2) | 1412.3 (1163.7-1729.9) | 930232.5 (765496.3-1140118.1) | 1405.8 (1156.8-1722.9) | -0.03 (-0.05--0.01) |
| Eastern Sub-Saharan Africa | 714036.3 (574870.2-910070.1) | 1007.2 (810.9-1283.8) | 1770129.7 (1427381-2254848.1) | 1010.4 (814.8-1287.1) | 0.03 (0.02-0.03) |
| High-income Asia Pacific | 833909.9 (680679.5-1036977.6) | 1235.5 (1008.5-1536.4) | 615406.1 (505742.6-759216.4) | 1217.7 (1000.7-1502.2) | -0.05 (-0.1--0.01) |
| High-income North America | 2076977.9 (1718556.6-2481454.7) | 1832.9 (1516.6-2189.9) | 2251563.9 (1882060.1-2723683.8) | 1827.8 (1527.8-2211.1) | 0.01 (0-0.03) |
| North Africa and Middle East | 1982727 (1568044.2-2553648.8) | 1481.5 (1171.7-1908.1) | 3712444.2 (2955469.2-4742993.8) | 1460.1 (1162.4-1865.4) | -0.05 (-0.06--0.05) |
| Oceania | 40642.6 (31829.4-52568) | 1530 (1198.2-1978.9) | 85844.5 (67928.8-110454.3) | 1523.6 (1205.6-1960.3) | -0.01 (-0.01--0.01) |
| South Asia | 6717246.8 (5547679-8172146.9) | 1556.3 (1285.3-1893.4) | 12338418.6 (10233232-15064333.5) | 1560 (1293.8-1904.6) | -0.01 (-0.05-0.02) |
| Southeast Asia | 3483442.7 (2842675.4-4339023.9) | 1768.2 (1442.9-2202.5) | 4803616.7 (3958518.4-5915952.5) | 1732.1 (1427.4-2133.2) | -0.07 (-0.07--0.06) |
| Southern Latin America | 246040.7 (196503.8-315416.9) | 1289.6 (1029.9-1653.2) | 335763.5 (267617.3-427657.8) | 1301.6 (1037.4-1657.9) | 0.07 (0.05-0.09) |
| Southern Sub-Saharan Africa | 298911.1 (243597.2-367331.4) | 1382.9 (1127-1699.4) | 462392.3 (378264.2-572084.4) | 1358.5 (1111.4-1680.8) | -0.07 (-0.08--0.06) |
| Tropical Latin America | 881213.7 (711879-1093015.8) | 1370.2 (1106.9-1699.5) | 1195548.3 (975238.8-1474902.3) | 1353.8 (1104.3-1670.1) | -0.14 (-0.22--0.07) |
| Western Europe | 2631002.4 (2142163.5-3269714) | 1825.6 (1486.4-2268.7) | 2364936.3 (1936028.9-2947503) | 1822.4 (1491.9-2271.3) | 0.03 (0.01-0.05) |
| Western Sub-Saharan Africa | 1077809.7 (867668.4-1348678.5) | 1505.9 (1212.3-1884.3) | 2896276.5 (2331792.5-3640004.2) | 1514.7 (1219.5-1903.7) | 0.01 (0.01-0.02) |
