# Supplementary material for: Global, regional, and national burden and trends of migraine among youths and young adults aged 15–39 years from 1990 to 2021: findings from the global burden of disease study 2021
Source: J Headache Pain. 2024 Aug 12;25(1):131. doi: 10.1186/s10194-024-01832-0 (PMC11318134; doi:10.1186/s10194-024-01832-0)
Supplement: Supplementary file 24 — Supplementary Material 24: Table S2 Disability-adjusted life years of Migraine Between 1990 and 2021 in 15 to 39 years at the Global and Regional Level [file 10194_2024_1832_MOESM24_ESM.docx]

| **TableS2 Disability-adjusted life years of Migraine Between 1990 and 2021 in 15 to 39 years at the Global and Regional Level** | | | | | |
| --- | --- | --- | --- | --- | --- |
| **Location** | **1990** | | **2021** | | **EAPC_95%CI** |
|  | **Num**ber(95%UI) | **ASR**(95%UI) | **Num**ber(95%UI) | **ASR**(95%UI) |  |
| Global | 15755634.7 (1778782.6-35638665.1) | 718.8 (81.2-1626) | 21973190.4 (2434456.8-49068576.4) | 738.6 (81.8-1649.5) | 0.09 (0.08-0.11) |
| High SDI | 2845523.8 (329244.7-6409714) | 820.1 (94.9-1847.4) | 2924716.9 (343906.9-6550737.2) | 828 (97.4-1854.5) | 0.02 (-0.02-0.06) |
| High-middle SDI | 3089050.8 (424929.5-6900007.4) | 682.6 (93.9-1524.7) | 3147256.5 (428425.9-6958900.8) | 714.9 (97.3-1580.6) | 0.17 (0.16-0.19) |
| Middle SDI | 5231857.3 (563240.7-11825053.3) | 695.1 (74.8-1571.2) | 6953719.5 (728469.3-15548979.6) | 749.7 (78.5-1676.5) | 0.25 (0.24-0.26) |
| Low-middle SDI | 3396035.6 (342631.7-7664346.1) | 749 (75.6-1690.4) | 6043366.5 (616672.8-13632805) | 753.1 (76.8-1698.8) | 0.01 (0-0.02) |
| Low SDI | 1178519.3 (145139.8-2711124.5) | 639.4 (78.7-1471) | 2887022.2 (350809.3-6645470) | 642.9 (78.1-1479.9) | 0.03 (0.02-0.04) |
| Andean Latin America | 78505.1 (11572.9-185996.8) | 507.7 (74.8-1202.8) | 146281.8 (20397.1-341274.9) | 540.2 (75.3-1260.3) | 0.25 (0.19-0.31) |
| Australasia | 57302.3 (7186.7-134040.8) | 702.8 (88.1-1643.9) | 74352 (9444.1-168880.4) | 710.1 (90.2-1612.9) | 0.01 (0.01-0.02) |
| Caribbean | 109461.6 (11147-251160.4) | 736.4 (75-1689.6) | 133054.2 (13709.8-303098.9) | 731 (75.3-1665.1) | -0.02 (-0.02--0.01) |
| Central Asia | 194271.7 (25316.4-468500.9) | 682.8 (89-1646.5) | 258003.4 (34218.1-610117.7) | 690.1 (91.5-1631.9) | 0.03 (0.01-0.04) |
| Central Europe | 328884 (50953.6-773955.6) | 702 (108.8-1652) | 249195 (39602.6-575719.2) | 711.6 (113.1-1644) | 0.08 (0.07-0.1) |
| Central Latin America | 492904.4 (53549.7-1119592.5) | 722 (78.4-1640) | 739139 (79893.4-1678151.4) | 730.6 (79-1658.9) | 0.05 (0.04-0.06) |
| Central Sub-Saharan Africa | 128157.7 (16642.2-293156.7) | 617.3 (80.2-1412) | 337375.3 (42792.9-776525.8) | 623.7 (79.1-1435.5) | 0.05 (0.04-0.06) |
| East Asia | 3165351.1 (382451.8-7230696.3) | 559.5 (67.6-1278.2) | 2962532.5 (344137.5-6587149.8) | 618.4 (71.8-1375) | 0.32 (0.28-0.35) |
| Eastern Europe | 644666.5 (132843.2-1434201.6) | 751.6 (154.9-1672.2) | 505048.5 (107406.9-1108670.2) | 763.2 (162.3-1675.4) | 0.11 (0.08-0.13) |
| Eastern Sub-Saharan Africa | 320583.6 (56602.7-755824.8) | 452.2 (79.8-1066.2) | 801473.3 (137331.7-1899209.9) | 457.5 (78.4-1084.1) | 0.08 (0.07-0.1) |
| High-income Asia Pacific | 403454.4 (59878.9-950399.4) | 597.8 (88.7-1408.1) | 305945.5 (45688-715056.2) | 605.4 (90.4-1414.8) | 0.01 (0-0.02) |
| High-income North America | 1044592 (107101.7-2347913.8) | 921.8 (94.5-2072) | 1101313.9 (113820.2-2487057) | 894 (92.4-2019) | -0.05 (-0.13-0.04) |
| North Africa and Middle East | 1109859.5 (165798.8-2526668) | 829.3 (123.9-1888) | 2141908.2 (321655.1-4737343.3) | 842.4 (126.5-1863.1) | 0.07 (0.05-0.08) |
| Oceania | 18916.3 (1806-44437) | 712.1 (68-1672.8) | 40614.6 (3858.2-93888.1) | 720.8 (68.5-1666.3) | 0.03 (0.03-0.04) |
| South Asia | 3178645 (299387.7-7293265.5) | 736.5 (69.4-1689.8) | 5886568.9 (549405.8-13362984.9) | 744.3 (69.5-1689.5) | 0.01 (-0.02-0.04) |
| Southeast Asia | 1691586.2 (137684.7-3942825.6) | 858.7 (69.9-2001.4) | 2367711 (194550.7-5514268.6) | 853.8 (70.2-1988.4) | -0.02 (-0.03--0.01) |
| Southern Latin America | 114759 (16620.4-266173.4) | 601.5 (87.1-1395.1) | 160486.1 (22914.6-370000.7) | 622.1 (88.8-1434.4) | 0.16 (0.13-0.18) |
| Southern Sub-Saharan Africa | 140466.8 (18848.7-315341.3) | 649.8 (87.2-1458.9) | 221948.2 (30353.9-494188.7) | 652.1 (89.2-1452) | 0.02 (0-0.03) |
| Tropical Latin America | 601773.9 (46919.2-1378153.7) | 935.7 (73-2142.9) | 828118.6 (65238.7-1885210.8) | 937.7 (73.9-2134.8) | 0.08 (0.04-0.12) |
| Western Europe | 1392784.1 (140521.2-3141049.7) | 966.4 (97.5-2179.5) | 1265848.1 (131715.7-2859519.2) | 975.4 (101.5-2203.5) | 0.04 (0-0.08) |
| Western Sub-Saharan Africa | 538709.4 (59850.2-1244472.2) | 752.7 (83.6-1738.7) | 1446272.3 (160391-3332916.6) | 756.4 (83.9-1743.1) | 0.02 (0.02-0.03) |
