# Supplementary material for: Global, regional, and national burden and trends of migraine among youths and young adults aged 15–39 years from 1990 to 2021: findings from the global burden of disease study 2021
Source: J Headache Pain. 2024 Aug 12;25(1):131. doi: 10.1186/s10194-024-01832-0 (PMC11318134; doi:10.1186/s10194-024-01832-0)
Supplement: Supplementary file 25 — Supplementary Material 25: Table S3 Percentage change in migraine prevalence in 204 countries [file 10194_2024_1832_MOESM25_ESM.docx]

| **TableS3 Percentage change in migraine prevalence in 204 countries** | | | |
| --- | --- | --- | --- |
| location | case_1990 | case_2021 | percent change |
| Qatar | 44910.04602 | 312443.21 | 595.7089508 |
| Equatorial Guinea | 26060.09674 | 115468.7385 | 343.086377 |
| United Arab Emirates | 184591.0128 | 764046.7004 | 313.9132717 |
| Afghanistan | 672110.0417 | 2555091.722 | 280.1597303 |
| Jordan | 317224.1059 | 1115410.61 | 251.6159678 |
| Cameroon | 767979.6317 | 2591360.925 | 237.4257361 |
| Niger | 560862.0349 | 1776590.733 | 216.7607402 |
| Djibouti | 21144.92898 | 66900.69973 | 216.3912246 |
| Angola | 664777.475 | 2083961.049 | 213.4825002 |
| Maldives | 17682.52935 | 55418.19703 | 213.4065039 |
| Benin | 347299.1843 | 1054634.624 | 203.6674638 |
| Yemen | 980086.6644 | 2928351.669 | 198.7849723 |
| Chad | 424189.4135 | 1262271.372 | 197.5725777 |
| Mali | 604984.9713 | 1787086.376 | 195.3935156 |
| Palestine | 160948.2624 | 461682.594 | 186.8515554 |
| Saudi Arabia | 1317525.016 | 3742573.909 | 184.0609372 |
| Somalia | 353978.4704 | 1002586.801 | 183.2338361 |
| Oman | 162233.0078 | 458442.3963 | 182.5826892 |
| Burkina Faso | 646935.0396 | 1753264.43 | 171.0108933 |
| Zambia | 408005.6648 | 1103318.767 | 170.4175118 |
| Uganda | 787566.0394 | 2111702.564 | 168.1302212 |
| Bahrain | 51782.27743 | 138749.5695 | 167.947986 |
| Kuwait | 174602.7737 | 466567.1247 | 167.2163303 |
| Gambia | 76039.13183 | 201480.641 | 164.9696757 |
| Nigeria | 7184400.702 | 18869169.41 | 162.6408269 |
| Papua New Guinea | 320584.8602 | 836412.9919 | 160.9022121 |
| Belize | 14515.06697 | 37682.87967 | 159.6121655 |
| Madagascar | 556969.6522 | 1445654.198 | 159.5570857 |
| Honduras | 336105.3891 | 860604.8319 | 156.0520776 |
| Ethiopia | 2061874.22 | 5217630.63 | 153.0528089 |
| Burundi | 257841.6225 | 651217.0624 | 152.5647551 |
| Tanzania | 1177351.13 | 2959678.296 | 151.3845038 |
| Democratic Republic of the Congo | 2435496.137 | 6112960.451 | 150.9944631 |
| Ghana | 1157021.237 | 2897770.378 | 150.4509238 |
| Guinea | 419073.8699 | 1049537.92 | 150.4422241 |
| Kenya | 1126375.63 | 2818888.851 | 150.2618821 |
| Mozambique | 594775.9707 | 1488044.083 | 150.1856423 |
| Togo | 276421.458 | 679724.303 | 145.9014246 |
| Pakistan | 8176169.834 | 20048658.45 | 145.2084394 |
| Iraq | 1500730.064 | 3674182.185 | 144.8263196 |
| Sudan | 1626710.049 | 3938443.997 | 142.1110018 |
| Liberia | 186325.6731 | 450499.7694 | 141.7808355 |
| C么te d'Ivoire | 945985.921 | 2246940.639 | 137.5236871 |
| Republic of Congo | 160815.9491 | 381176.6412 | 137.0266403 |
| Senegal | 556175.1586 | 1290485.373 | 132.0285891 |
| Sierra Leone | 325127.1526 | 750903.9371 | 130.9570059 |
| Guatemala | 576915.4746 | 1329586.059 | 130.4646205 |
| Guinea-Bissau | 74913.78251 | 170591.6444 | 127.7173021 |
| Haiti | 491629.4385 | 1098757.105 | 123.4929439 |
| Mauritania | 154710.1487 | 343726.7622 | 122.1746699 |
| Malawi | 460068.7416 | 1006369.306 | 118.7432475 |
| Solomon Islands | 24661.03944 | 53339.10549 | 116.288959 |
| Eritrea | 159244.3928 | 343960.2151 | 115.9951814 |
| Vanuatu | 11438.19678 | 24406.23676 | 113.3748635 |
| Sao Tome and Principe | 8567.928421 | 18215.08582 | 112.5961484 |
| Central African Republic | 178227.1143 | 374049.0034 | 109.8721089 |
| Botswana | 88384.60973 | 184637.3335 | 108.9021314 |
| Laos | 341161.0684 | 710685.1674 | 108.3136774 |
| Rwanda | 338593.6495 | 703847.5478 | 107.8738183 |
| Egypt | 4684923.449 | 9460165.539 | 101.9278573 |
| Lebanon | 248482.0519 | 499061.1651 | 100.8439488 |
| Tajikistan | 390781.1729 | 777754.8886 | 99.02568048 |
| Gabon | 65338.05332 | 129643.3218 | 98.41932107 |
| Bolivia | 370274.7666 | 731637.7158 | 97.59318801 |
| Paraguay | 388606.0467 | 748093.3044 | 92.50686156 |
| Malaysia | 1465149.096 | 2819797.677 | 92.45807024 |
| Cabo Verde | 26349.13933 | 50428.07912 | 91.38416054 |
| Nicaragua | 288471.0189 | 549852.5062 | 90.60927097 |
| Namibia | 95263.23384 | 180057.7712 | 89.01076935 |
| Cambodia | 858987.1594 | 1610706.838 | 87.51233016 |
| Peru | 1062441.635 | 1966608.418 | 85.10272491 |
| Nepal | 1567266.552 | 2896832.215 | 84.83341026 |
| Libya | 346874.188 | 640653.5113 | 84.69333647 |
| Ecuador | 671708.4889 | 1232252.031 | 83.45041809 |
| Philippines | 5888440.098 | 10791921.37 | 83.27300931 |
| Comoros | 21173.78541 | 38480.2886 | 81.73551804 |
| India | 70187747.06 | 126198713.5 | 79.80163027 |
| Timor-Leste | 69788.10867 | 124466.6341 | 78.34934419 |
| Israel | 492153.5637 | 854595.0254 | 73.64397791 |
| Algeria | 2124923.897 | 3670213.099 | 72.72209623 |
| Eswatini | 51889.19488 | 87742.54012 | 69.09597521 |
| Iran | 4856906.508 | 8088631.074 | 66.53874355 |
| Bangladesh | 8433147.303 | 14021125.24 | 66.26206962 |
| Brunei Darussalam | 18643.85416 | 30974.99592 | 66.14051819 |
| Cyprus | 79033.0143 | 131135.5988 | 65.92508831 |
| Kiribati | 5999.96042 | 9816.531499 | 63.60993759 |
| Panama | 194658.2671 | 316934.4203 | 62.81580286 |
| Uzbekistan | 1591591.702 | 2585395.276 | 62.44086174 |
| Zimbabwe | 675142.9369 | 1090534.498 | 61.52646185 |
| South Sudan | 279320.8055 | 440889.2056 | 57.84331027 |
| South Africa | 2806947.544 | 4382065.07 | 56.11496121 |
| Kyrgyzstan | 335124.9161 | 510246.11 | 52.25549802 |
| Lesotho | 94093.75889 | 142322.2825 | 51.25581564 |
| Singapore | 183548.0067 | 276881.405 | 50.84958424 |
| Argentina | 1963206.154 | 2940947.723 | 49.80330603 |
| Costa Rica | 248609.7207 | 372023.8735 | 49.64172456 |
| Luxembourg | 37215.36549 | 55603.29708 | 49.409515 |
| Indonesia | 17852206.22 | 26234884.03 | 46.95597683 |
| Mongolia | 162767.7731 | 238314.6001 | 46.41387277 |
| Mexico | 6917584.063 | 10119349.03 | 46.28443895 |
| Dominican Republic | 617757.2077 | 899699.6571 | 45.63968592 |
| Colombia | 2766537.821 | 3977098.24 | 43.75723367 |
| Morocco | 2211513.111 | 3147777.233 | 42.33590645 |
| Marshall Islands | 3318.764165 | 4628.741474 | 39.47184083 |
| Brazil | 16148820.36 | 22070991.95 | 36.67247181 |
| Vietnam | 6318518.445 | 8605913.335 | 36.20144359 |
| Turkey | 4957077.198 | 6676476.103 | 34.68573992 |
| Azerbaijan | 595842.0329 | 799250.275 | 34.13794779 |
| Turkmenistan | 284830.8142 | 381576.253 | 33.96593132 |
| Tuvalu | 718.8787238 | 959.9774396 | 33.53816269 |
| Suriname | 32090.91233 | 42729.7924 | 33.15231415 |
| Antigua and Barbuda | 5166.211387 | 6843.313079 | 32.46289333 |
| Bhutan | 52787.59735 | 69749.41879 | 32.13220965 |
| Myanmar | 3788176.133 | 5000996.57 | 32.01594632 |
| Bahamas | 23545.32824 | 30912.54207 | 31.28949299 |
| Saint Kitts and Nevis | 3437.873512 | 4504.277852 | 31.01930121 |
| New Zealand | 278286.1868 | 363229.256 | 30.52363835 |
| Tunisia | 729043.1077 | 945318.8172 | 29.66569566 |
| Australia | 1308543.424 | 1689626.705 | 29.12270802 |
| Chile | 948569.9062 | 1209587.192 | 27.51692673 |
| El Salvador | 408702.1041 | 509256.0882 | 24.603246 |
| Venezuela | 1506614.987 | 1858046.944 | 23.32593002 |
| Seychelles | 6848.656739 | 8419.647498 | 22.93866986 |
| Samoa | 12740.5706 | 15596.53163 | 22.4162725 |
| Norway | 383559.95 | 467600.9713 | 21.91079159 |
| Jamaica | 196824.3358 | 237098.659 | 20.46206482 |
| Grenada | 6604.34155 | 7915.669338 | 19.85554167 |
| North Korea | 1370832.053 | 1634799.372 | 19.25599266 |
| Saint Lucia | 11261.36631 | 13045.54079 | 15.8433216 |
| Ireland | 352094.6471 | 406619.3783 | 15.48581657 |
| Iceland | 26759.34779 | 30776.72004 | 15.01296773 |
| Nauru | 790.6267235 | 908.4230428 | 14.89910672 |
| Fiji | 63010.12383 | 70045.62759 | 11.16567201 |
| Sweden | 767331.4272 | 850426.6884 | 10.82912263 |
| Sri Lanka | 1635575.044 | 1801420.985 | 10.13991631 |
| Syria | 1006450.777 | 1104901.469 | 9.781967961 |
| Tonga | 7098.118144 | 7604.403836 | 7.132674918 |
| Switzerland | 612843.696 | 656532.3129 | 7.128835153 |
| USA | 25841111.57 | 27637644.03 | 6.952225918 |
| Micronesia (Federated States of) | 7759.478814 | 8237.015152 | 6.154232124 |
| Uruguay | 190989.0611 | 202266.0893 | 5.904541391 |
| UK | 5339957.024 | 5630049.573 | 5.432488459 |
| Canada | 2742744.084 | 2875100.844 | 4.825705806 |
| Kazakhstan | 1269675.056 | 1319894.849 | 3.955326371 |
| Andorra | 6368.176451 | 6586.901912 | 3.43466396 |
| Belgium | 1090642.535 | 1103609.287 | 1.18890936 |
| Monaco | 2398.574639 | 2420.567837 | 0.916927836 |
| Trinidad and Tobago | 99371.35617 | 98405.61114 | -0.971854535 |
| Denmark | 422989.1687 | 417865.4163 | -1.211320005 |
| Macedonia | 146947.0237 | 143220.618 | -2.535883744 |
| Malta | 35613.58426 | 34669.37606 | -2.651258557 |
| San Marino | 2443.970408 | 2332.050488 | -4.579430207 |
| Austria | 734216.291 | 689824.0314 | -6.046210109 |
| Netherlands | 1416164.629 | 1327909.931 | -6.231951923 |
| China | 81605907.96 | 75970637.6 | -6.905468614 |
| Mauritius | 110218.0185 | 101408.2703 | -7.993019954 |
| Finland | 470364.761 | 429635.4335 | -8.659094153 |
| Guyana | 67919.811 | 61936.58766 | -8.80924616 |
| Barbados | 21746.02303 | 19662.21433 | -9.582481807 |
| Saint Vincent and the Grenadines | 9087.662371 | 8162.883176 | -10.17620547 |
| France | 5685345.006 | 5073408.06 | -10.76340916 |
| Dominica | 5735.760331 | 5102.653891 | -11.0378817 |
| Guam | 12284.34616 | 10829.01188 | -11.84706342 |
| Germany | 8159357.554 | 7106517.647 | -12.90346575 |
| American Samoa | 3951.783575 | 3390.617381 | -14.2003271 |
| Tokelau | 112.7670923 | 96.4137431 | -14.50188069 |
| Taiwan | 1605815.999 | 1368040.84 | -14.80712356 |
| Poland | 2829322.971 | 2399460.516 | -15.19312074 |
| Slovakia | 381313.4324 | 322719.8721 | -15.36624608 |
| Spain | 3939341.978 | 3332103.808 | -15.41471073 |
| Palau | 1357.388332 | 1124.782512 | -17.13627664 |
| Montenegro | 46287.03008 | 38327.25251 | -17.19656143 |
| Serbia | 666715.0632 | 549044.6625 | -17.64927886 |
| Russia | 11382280.65 | 9195204.689 | -19.2147429 |
| Czech Republic | 689075.2969 | 554921.2603 | -19.46870497 |
| Greenland | 6251.067765 | 4929.080103 | -21.14818958 |
| Portugal | 978735.6679 | 768365.9449 | -21.49402846 |
| Cook Islands | 1495.055153 | 1171.949005 | -21.61165409 |
| Thailand | 6902799.466 | 5228224.259 | -24.25936339 |
| Armenia | 270932.0442 | 204941.3909 | -24.35690229 |
| Belarus | 700733.4725 | 526648.8651 | -24.84319848 |
| Hungary | 690705.3736 | 515858.5138 | -25.31424635 |
| Slovenia | 143090.0304 | 106720.7346 | -25.41707178 |
| Greece | 1004689.845 | 747429.8206 | -25.60591468 |
| Italy | 5981706.718 | 4447603.941 | -25.64657295 |
| Japan | 6928111.706 | 5147133.365 | -25.70654771 |
| Ukraine | 3526129.591 | 2590757.235 | -26.52688541 |
| South Korea | 3797550.957 | 2790035.979 | -26.53065067 |
| Cuba | 966979.8486 | 704983.4471 | -27.09429797 |
| Puerto Rico | 283611.2242 | 206154.3029 | -27.31095056 |
| Moldova | 311394.3288 | 222891.2971 | -28.42152973 |
| Niue | 155.4718359 | 110.817847 | -28.72159365 |
| Estonia | 100530.395 | 70168.88637 | -30.20132231 |
| Northern Mariana Islands | 4654.49015 | 3219.898453 | -30.82167222 |
| Croatia | 339021.1384 | 233566.9891 | -31.10547909 |
| Bermuda | 5112.484946 | 3486.547422 | -31.80327259 |
| Albania | 261644.3805 | 174588.8452 | -33.27246514 |
| Bulgaria | 555142.5658 | 356803.2319 | -35.72763937 |
| Romania | 1610887.234 | 1005631.582 | -37.57281323 |
| Lithuania | 232569.5765 | 135080.3099 | -41.91832314 |
| United States Virgin Islands | 7985.957974 | 4616.796293 | -42.18857264 |
| Latvia | 168851.3532 | 95961.13146 | -43.1682781 |
| Bosnia and Herzegovina | 350492.9192 | 188415.1245 | -46.24281572 |
| Georgia | 401993.2524 | 213590.5251 | -46.86713675 |
