# Supplementary material for: Global, regional, and national burden and trends of migraine among youths and young adults aged 15–39 years from 1990 to 2021: findings from the global burden of disease study 2021
Source: J Headache Pain. 2024 Aug 12;25(1):131. doi: 10.1186/s10194-024-01832-0 (PMC11318134; doi:10.1186/s10194-024-01832-0)
Supplement: Supplementary file 26 — Supplementary Material 26: Table S4 Prevalence of Migraine Between 1990 and 2021 in 15 to 39 years at the 204 Countries Level [file 10194_2024_1832_MOESM26_ESM.docx]

| **TableS4 Prevalence of Migraine Between 1990 and 2021 in 15 to 39 years at the 204 Countries Level** | | | | | |
| --- | --- | --- | --- | --- | --- |
| **location** | **1990** | | **2021** | | **EAPC_95%CI** |
|  | **Number** | **ASR** | **Number** | **ASR** |  |
| Maldives | 17682.5 (14143.3-21804.2) | 21758.8 (17403.7-26830.6) | 55418.2 (44903.6-68201.2) | 21301.1 (17259.6-26214.5) | -0.09 (-0.11--0.07) |
| Myanmar | 3788176.1 (3035072.8-4636226.9) | 22066.1 (17679.3-27006) | 5000996.6 (4017474-6096296.2) | 22244.7 (17870-27116.7) | 0.02 (0.02-0.03) |
| Papua New Guinea | 320584.9 (258540.9-398943.4) | 19368.4 (15620-24102.5) | 836413 (674820.5-1036436.2) | 19544.5 (15768.5-24218.4) | 0.02 (0.01-0.02) |
| Philippines | 5888440.1 (4863258.8-7055942.6) | 22718.6 (18763.3-27223) | 10791921.4 (8921282.8-12924637.2) | 22839.9 (18880.9-27353.6) | 0.01 (0-0.01) |
| Sri Lanka | 1635575 (1311199.3-1995104.5) | 22118.5 (17731.8-26980.5) | 1801421 (1450731.4-2194316.5) | 22337.3 (17988.8-27209.1) | 0.05 (0.04-0.05) |
| Samoa | 12740.6 (10242.3-15910.3) | 19026 (15295.2-23759.4) | 15596.5 (12579.4-19337) | 19411.2 (15656.1-24066.5) | 0.05 (0.04-0.06) |
| Romania | 1610887.2 (1301127.2-1961640) | 18543.6 (14977.8-22581.3) | 1005631.6 (814615.8-1229655.1) | 18660 (15115.6-22816.8) | 0.06 (0.05-0.07) |
| Mongolia | 162767.8 (130363.7-202135.4) | 18419.4 (14752.4-22874.4) | 238314.6 (192609.8-293832.3) | 18881.9 (15260.7-23280.6) | 0.07 (0.06-0.09) |
| Serbia | 666715.1 (538367.7-814632.5) | 18565.5 (14991.5-22684.5) | 549044.7 (444812.3-671232.5) | 18522 (15005.7-22644) | 0.02 (0.01-0.03) |
| Montenegro | 46287 (37344.5-56515.6) | 18439.2 (14876.8-22514) | 38327.3 (30985.3-46834.5) | 18630 (15061.2-22765.2) | 0.04 (0.04-0.05) |
| Belgium | 1090642.5 (897102.3-1324122.6) | 29313.7 (24111.8-35589) | 1103609.3 (914299.9-1349429.2) | 31509.1 (26104.1-38527.4) | 0.25 (0.18-0.31) |
| Solomon Islands | 24661 (19853.6-30789.8) | 19226.4 (15478.4-24004.5) | 53339.1 (43016.1-66080.2) | 19501.1 (15726.9-24159.3) | 0.06 (0.05-0.07) |
| Central African Republic | 178227.1 (142882.5-221056.3) | 17115.2 (13721-21228.1) | 374049 (299905.3-462957.3) | 17132.8 (13736.8-21205.2) | 0.01 (0-0.02) |
| Andorra | 6368.2 (5188.1-7913.5) | 25463.2 (20744.6-31642.4) | 6586.9 (5415.6-8152.8) | 25833.8 (21239.9-31975.4) | 0.03 (0-0.06) |
| Cyprus | 79033 (64481-97877.3) | 25697 (20965.5-31824.1) | 131135.6 (108000.2-163062.3) | 26135.8 (21524.8-32498.9) | 0.07 (0.06-0.08) |
| Cuba | 966979.8 (774614.8-1184417.7) | 19816.5 (15874.3-24272.5) | 704983.4 (572816.1-859154.1) | 19661.4 (15975.3-23961) | -0.02 (-0.02--0.02) |
| Argentina | 1963206.2 (1609519.2-2404920.1) | 16071.8 (13176.4-19688) | 2940947.7 (2404672.2-3601555.1) | 16783.8 (13723.3-20553.8) | 0.2 (0.16-0.23) |
| Yemen | 980086.7 (802921.1-1202280.9) | 21320.7 (17466.7-26154.3) | 2928351.7 (2399034-3584790) | 21282.1 (17435.3-26052.9) | 0.03 (0.01-0.05) |
| Russian Federation | 11382280.7 (9535226-13526614.7) | 19566.8 (16391.6-23253) | 9195204.7 (7730659.8-10921783.9) | 19785.2 (16633.9-23500.2) | 0.09 (0.06-0.11) |
| Tonga | 7098.1 (5712.4-8876.8) | 19237.8 (15482.1-24058.4) | 7604.4 (6129-9428.4) | 19549.9 (15756.8-24239) | 0.06 (0.06-0.06) |
| Thailand | 6902799.5 (5676907.6-8323093.9) | 26618.8 (21891.5-32095.8) | 5228224.3 (4262288.7-6369186) | 24661.8 (20105.4-30043.7) | -0.28 (-0.37--0.2) |
| Dominica | 5735.8 (4590.5-7010.6) | 19639.1 (15717.9-24004.1) | 5102.7 (4120.7-6218.7) | 19702.7 (15910.9-24011.9) | 0.02 (0.01-0.03) |
| Slovakia | 381313.4 (308271.6-465103.1) | 18627.4 (15059.2-22720.6) | 322719.9 (263195-396000) | 18858.1 (15379.8-23140.2) | 0.07 (0.05-0.08) |
| Turkmenistan | 284830.8 (228550.6-352860.5) | 18555.2 (14888.8-22986.9) | 381576.3 (306726.5-470793.2) | 18346.2 (14747.4-22635.7) | -0.06 (-0.08--0.05) |
| Botswana | 88384.6 (70792.6-109602.2) | 17169.9 (13752.4-21291.7) | 184637.3 (148061.8-227021.7) | 17330.8 (13897.6-21309.1) | 0.04 (0.03-0.05) |
| Denmark | 422989.2 (348019.6-514929.6) | 22170.1 (18240.8-26989) | 417865.4 (341416.2-510373.9) | 22913.6 (18721.5-27986.2) | 0.1 (0.06-0.14) |
| Slovenia | 143090 (115667.2-175052) | 18672 (15093.5-22842.7) | 106720.7 (86803-130753) | 18742.7 (15244.6-22963.3) | 0.02 (0.01-0.03) |
| Kenya | 1126375.6 (926510.4-1351381.7) | 12861.7 (10579.5-15431) | 2818888.9 (2328261.1-3371323.6) | 13019.3 (10753.3-15570.8) | 0.05 (0.05-0.06) |
| Tajikistan | 390781.2 (313072.2-485263.5) | 18479.6 (14804.9-22947.6) | 777754.9 (625174.5-959442) | 18646 (14988-23001.8) | 0 (-0.01-0.02) |
| Brunei Darussalam | 18643.9 (14973.6-22938.2) | 15122.1 (12145.2-18605.3) | 30975 (24954.5-38109) | 15180.9 (12230.2-18677.3) | -0.02 (-0.05-0) |
| Finland | 470364.8 (384911.3-583155.3) | 25909.3 (21202.3-32122.2) | 429635.4 (351788.7-532362.7) | 25788.5 (21115.8-31954.6) | -0.01 (-0.02-0) |
| Japan | 6928111.7 (5704766.3-8253496) | 15459.5 (12729.7-18417) | 5147133.4 (4289386.5-6085668.9) | 15881.2 (13234.7-18777.1) | 0.12 (0.09-0.14) |
| Cambodia | 858987.2 (687039.4-1052822.4) | 22309.7 (17843.8-27344) | 1610706.8 (1296158.7-1966255.4) | 22237.7 (17895-27146.4) | -0.01 (-0.04-0.01) |
| Uzbekistan | 1591591.7 (1277234.9-1971726) | 18542.5 (14880.2-22971.2) | 2585395.3 (2092278.9-3186154.6) | 18820.5 (15230.8-23193.7) | 0.04 (0.02-0.05) |
| Kuwait | 174602.8 (143297.9-215769.8) | 20660.3 (16956.1-25531.5) | 466567.1 (385874.6-568595.7) | 21979.5 (18178.2-26786) | 0.22 (0.16-0.27) |
| Madagascar | 556969.7 (453247.7-681448.7) | 12296.1 (10006.3-15044.3) | 1445654.2 (1177217.1-1768293.5) | 12344.7 (10052.4-15099.7) | 0.01 (0.01-0.02) |
| Austria | 734216.3 (594394.7-887984.2) | 24457.7 (19800.1-29579.9) | 689824 (560159.5-832580.3) | 24440.3 (19846.3-29498.1) | 0.05 (0.01-0.1) |
| Vanuatu | 11438.2 (9226.4-14198.1) | 19525.3 (15749.7-24236.6) | 24406.2 (19658.9-30257.5) | 19581.6 (15772.7-24276.2) | 0.02 (0.01-0.02) |
| Lesotho | 94093.8 (75596.3-116344.5) | 17438.7 (14010.5-21562.5) | 142322.3 (114261.9-175782.3) | 17109.7 (13736.3-21132.2) | -0.05 (-0.06--0.04) |
| Guyana | 67919.8 (54311.9-83121) | 19955.2 (15957.2-24421.5) | 61936.6 (49703.7-75927.6) | 19932.1 (15995.4-24434.7) | 0 (0-0.01) |
| Indonesia | 17852206.2 (14762256.5-21395565.3) | 22874.6 (18915.3-27414.8) | 26234884 (21703197.4-31320095.7) | 23038.4 (19058.9-27504.1) | 0.02 (0.02-0.03) |
| Chile | 948569.9 (768536.4-1168830.3) | 16562.2 (13418.8-20407.9) | 1209587.2 (980751.2-1483202.6) | 17095.5 (13861.3-20962.6) | 0.13 (0.11-0.15) |
| Lao People's Democratic Republic | 341161.1 (273250.3-417273.7) | 22081.4 (17685.9-27007.7) | 710685.2 (569943.5-868117.3) | 22154.5 (17767.1-27062.2) | 0 (-0.01-0.02) |
| Sierra Leone | 325127.2 (262995.1-404356) | 20353.4 (16463.8-25313.2) | 750903.9 (605976.2-935207.8) | 20132.9 (16247.2-25074.4) | -0.06 (-0.07--0.05) |
| Ukraine | 3526129.6 (2935332.2-4178512.9) | 18564 (15453.6-21998.6) | 2590757.2 (2178340.3-3085204.8) | 18794.1 (15802.3-22380.9) | 0.06 (0.03-0.08) |
| Bolivia (Plurinational State of) | 370274.8 (300119.6-450772.8) | 15014.9 (12170-18279.1) | 731637.7 (594634.5-884971.8) | 14888.9 (12100.9-18009.3) | -0.03 (-0.04--0.03) |
| Uruguay | 190989.1 (153819.1-238080) | 16815.1 (13542.6-20961.1) | 202266.1 (163256.1-252211.4) | 16908.6 (13647.5-21083.8) | 0.02 (0.02-0.02) |
| Haiti | 491629.4 (394255.9-601463.7) | 20196.1 (16196-24708) | 1098757.1 (884924.9-1341137.2) | 20016.1 (16120.7-24431.5) | -0.03 (-0.04--0.03) |
| Ecuador | 671708.5 (557120.8-816535.5) | 16278.6 (13501.6-19788.4) | 1232252 (994884.1-1517641.4) | 16869.8 (13620.2-20776.9) | 0.17 (0.14-0.2) |
| Jamaica | 196824.3 (157260.7-240898.6) | 20027.8 (16002-24512.5) | 237098.7 (191275.4-289342.9) | 19870 (16029.8-24248.4) | -0.03 (-0.03--0.02) |
| Venezuela (Bolivarian Republic of) | 1506615 (1247253.2-1831394.4) | 18811.2 (15572.9-22866.3) | 1858046.9 (1507799-2260592.1) | 19845.3 (16104.4-24144.7) | 0.16 (0.14-0.19) |
| American Samoa | 3951.8 (3185.6-4905) | 19532.9 (15745.9-24244.4) | 3390.6 (2733.2-4186.6) | 19403.2 (15641-23958.6) | -0.03 (-0.04--0.03) |
| Iceland | 26759.3 (21819.8-33119.6) | 25759.4 (21004.4-31881.9) | 30776.7 (25168-38177.6) | 25717.8 (21031-31902.2) | -0.01 (-0.02--0.01) |
| Puerto Rico | 283611.2 (228535.6-346397.7) | 20049.2 (16155.8-24487.8) | 206154.3 (167008.1-250972.7) | 19932.3 (16147.4-24265.7) | 0 (0-0) |
| Ireland | 352094.6 (286982.9-436463.2) | 25667.9 (20921.3-31818.5) | 406619.4 (333390.3-502571.6) | 25986.8 (21306.8-32119.1) | 0.07 (0.05-0.08) |
| Latvia | 168851.4 (138090.3-207424.7) | 17699.9 (14475.3-21743.3) | 95961.1 (78363.7-117884) | 17816.4 (14549.2-21886.7) | 0.02 (0-0.04) |
| Albania | 261644.4 (211555.1-319586) | 18419.5 (14893.2-22498.5) | 174588.8 (140952-214540.5) | 18415.1 (14867.2-22629.1) | -0.05 (-0.07--0.03) |
| Lithuania | 232569.6 (187693.7-284842.9) | 16691.7 (13471-20443.5) | 135080.3 (109495-166080.2) | 16778.2 (13600.3-20628.6) | -0.1 (-0.14--0.06) |
| Canada | 2742744.1 (2302088.1-3261022.4) | 24672.2 (20708.3-29334.4) | 2875100.8 (2353925.1-3497333.5) | 24238.7 (19844.9-29484.5) | -0.08 (-0.09--0.06) |
| Netherlands | 1416164.6 (1176462.4-1731873.3) | 23486.1 (19510.8-28722) | 1327909.9 (1085584.7-1627392.7) | 25147.8 (20558.7-30819.4) | 0.35 (0.26-0.45) |
| Poland | 2829323 (2347050.1-3381832.7) | 19586.9 (16248.2-23411.9) | 2399460.5 (1992442.2-2862236.4) | 19832.6 (16468.4-23657.7) | 0.08 (0.05-0.11) |
| Australia | 1308543.4 (1051183.3-1601973.1) | 19321.7 (15521.6-23654.4) | 1689626.7 (1359202.7-2076745.9) | 19489.3 (15678-23954.6) | 0.01 (0.01-0.02) |
| Italy | 5981706.7 (5009428.9-7086821) | 28019.5 (23465.1-33196) | 4447603.9 (3733036.4-5274410.6) | 28158.3 (23634.3-33392.9) | 0.16 (0.02-0.29) |
| Brazil | 16148820.4 (13460248.6-19241525.7) | 25736.9 (21452-30665.8) | 22070991.9 (18372765.4-26267214.2) | 25889.7 (21551.6-30812) | 0.07 (0.03-0.12) |
| Lebanon | 248482.1 (203760.1-305120.8) | 21555.1 (17675.6-26468.4) | 499061.2 (409234.4-612372.7) | 21508 (17636.8-26391.4) | -0.02 (-0.04--0.01) |
| Fiji | 63010.1 (50804.9-78144.8) | 19540.7 (15755.6-24234.3) | 70045.6 (56506.7-86215.5) | 19635.3 (15840.1-24168.1) | 0.03 (0.02-0.04) |
| Israel | 492153.6 (401174.6-610532.7) | 25754.2 (20993.3-31948.9) | 854595 (696998.6-1058810.3) | 25717.2 (20974.7-31862.6) | 0 (0-0.01) |
| New Zealand | 278286.2 (227954.6-331511.3) | 20144.3 (16500.9-23997.1) | 363229.3 (297549.2-430998.5) | 20163.8 (16517.7-23925.9) | 0 (-0.02-0.01) |
| Congo | 160815.9 (128684.2-199589.7) | 16975.7 (13583.9-21068.6) | 381176.6 (306533-469665.7) | 17204.1 (13835.1-21198) | 0.07 (0.06-0.08) |
| Saint Kitts and Nevis | 3437.9 (2760.1-4204.4) | 19893.6 (15971.7-24329.3) | 4504.3 (3659.8-5479.5) | 19818.9 (16103.1-24109.9) | -0.01 (-0.02--0.01) |
| Bermuda | 5112.5 (4172.8-6219.3) | 19881.1 (16226.8-24185.2) | 3486.5 (2856.6-4226.5) | 19913.3 (16315.5-24139.4) | 0.01 (0.01-0.01) |
| Togo | 276421.5 (222370.7-343508.7) | 20163.8 (16221-25057.6) | 679724.3 (548827.8-844944.8) | 20202.7 (16312.2-25113.4) | 0 (0-0) |
| Dominican Republic | 617757.2 (493569.2-755866.6) | 20109.3 (16066.7-24605) | 899699.7 (724443.7-1098542) | 19785.5 (15931.4-24158.3) | -0.06 (-0.07--0.06) |
| Peru | 1062441.6 (892088.6-1275204.5) | 11976.2 (10055.9-14374.5) | 1966608.4 (1604308.4-2382383.3) | 13233.3 (10795.4-16031) | 0.36 (0.26-0.46) |
| Kiribati | 6000 (4838.4-7447) | 19651.6 (15847-24391.1) | 9816.5 (7910-12118.4) | 19742.2 (15908-24371.5) | 0 (-0.02-0.01) |
| United States of America | 25841111.6 (21505458.1-30339206.6) | 25292.3 (21048.7-29694.9) | 27637644 (22991228.7-32629033.6) | 24831.6 (20657-29316.2) | -0.02 (-0.11-0.08) |
| Norway | 383559.9 (320359.5-455359.1) | 23975.3 (20024.8-28463.3) | 467601 (387793.1-551683.5) | 26347.3 (21850.5-31085) | 0.39 (0.31-0.47) |
| Costa Rica | 248609.7 (200281.4-303879.7) | 19353.1 (15591-23655.6) | 372023.9 (302799.6-455709.9) | 19546.4 (15909.3-23943.4) | 0.03 (0.03-0.04) |
| Trinidad and Tobago | 99371.4 (79831.5-121447.4) | 19820.8 (15923.4-24224.2) | 98405.6 (80635.4-119263) | 19768.9 (16199-23959) | -0.01 (-0.02--0.01) |
| Azerbaijan | 595842 (477566.4-736089.2) | 18748.2 (15026.7-23161.1) | 799250.3 (646930.9-987264) | 18868.5 (15272.6-23307.1) | -0.02 (-0.04-0) |
| Marshall Islands | 3318.8 (2675.4-4126.2) | 19348.1 (15597.5-24055.6) | 4628.7 (3727.6-5715.2) | 19513 (15714-24093.1) | 0.05 (0.04-0.06) |
| El Salvador | 408702.1 (330543.3-499306.8) | 19561.6 (15820.7-23898.1) | 509256.1 (411435.2-622297.8) | 19647.6 (15873.6-24008.9) | 0.01 (0-0.02) |
| Portugal | 978735.7 (797150.7-1213064.2) | 25850.7 (21054.6-32039.8) | 768365.9 (631749.1-951351.2) | 26032 (21403.5-32231.5) | 0.04 (0.03-0.05) |
| Malawi | 460068.7 (374255.9-563591.4) | 12312.9 (10016.3-15083.5) | 1006369.3 (817476.4-1231855.6) | 12298 (9989.7-15053.5) | 0.02 (0.01-0.02) |
| Namibia | 95263.2 (76246.5-118250.1) | 17035.3 (13634.7-21145.9) | 180057.8 (144316-221973.6) | 17229.1 (13809.1-21239.8) | 0.03 (0.02-0.04) |
| China | 81605908 (66625124.8-97320466.5) | 14887.8 (12154.8-17754.7) | 75970637.6 (62647973.5-90189567.4) | 16463.6 (13576.5-19545) | 0.32 (0.28-0.36) |
| Gambia | 76039.1 (61276.1-94766.1) | 20167.6 (16252-25134.4) | 201480.6 (162217.4-250458.7) | 20145.3 (16219.5-25042.5) | 0 (0-0.01) |
| Luxembourg | 37215.4 (30375.3-45504.7) | 25218.2 (20583.1-30835.2) | 55603.3 (45600-67967.4) | 25204.8 (20670.3-30809.4) | -0.04 (-0.07-0) |
| Libya | 346874.2 (281866.1-425723) | 20644.8 (16775.7-25337.6) | 640653.5 (525879.1-783988.8) | 21351.3 (17526.2-26128.3) | 0.09 (0.08-0.1) |
| South Africa | 2806947.5 (2299860-3364815.2) | 17838 (14615.5-21383.2) | 4382065.1 (3613695.4-5218761.3) | 18072.6 (14903.7-21523.3) | 0.03 (0.02-0.04) |
| Ghana | 1157021.2 (933304.9-1438422.4) | 20156.6 (16259.2-25058.9) | 2897770.4 (2342461.7-3599821.4) | 20261.7 (16378.9-25170.5) | 0 (0-0) |
| Oman | 162233 (131792.3-197792.4) | 19553.2 (15884.4-23839.1) | 458442.4 (374633.3-558229.8) | 19809.1 (16187.7-24120.9) | 0.03 (-0.02-0.07) |
| Georgia | 401993.3 (324157.2-495422) | 18882.2 (15226.1-23270.7) | 213590.5 (172998.7-263471.6) | 18823.7 (15246.3-23219.7) | -0.02 (-0.04--0.01) |
| Qatar | 44910 (36609-54550.9) | 18993.3 (15482.7-23070.6) | 312443.2 (255087.2-379909.4) | 18907.2 (15436.4-22989.9) | -0.19 (-0.27--0.12) |
| Timor-Leste | 69788.1 (55901.5-85422.6) | 21925.4 (17562.6-26837.3) | 124466.6 (99615.9-153275.1) | 21785.1 (17435.5-26827.4) | -0.04 (-0.05--0.03) |
| Cook Islands | 1495.1 (1207-1855.6) | 19371.1 (15638.9-24043.1) | 1171.9 (942.3-1445.5) | 19888.1 (15991.3-24530.5) | 0 (0-0) |
| Mauritius | 110218 (88485.2-134664.2) | 22171.5 (17799.7-27089.1) | 101408.3 (81778-123908.1) | 22277.3 (17965-27220.1) | 0.01 (0.01-0.02) |
| Kazakhstan | 1269675.1 (1021822.6-1564700.6) | 18702 (15051.2-23047.7) | 1319894.8 (1067496.6-1629460.1) | 18938.5 (15317-23380.3) | 0.04 (0.02-0.06) |
| Bhutan | 52787.6 (42349-65369.7) | 19590.3 (15716.4-24259.7) | 69749.4 (56258.1-86231.6) | 20124.3 (16231.8-24879.8) | 0.08 (0.07-0.09) |
| Burundi | 257841.6 (209657.1-315247) | 12438.1 (10113.7-15207.3) | 651217.1 (530946.5-797226.2) | 12351.8 (10070.6-15121.1) | -0.03 (-0.05-0) |
| India | 70187747.1 (57898981.4-83778323.7) | 20581.3 (16977.8-24566.5) | 126198713.5 (103993641-149958552.6) | 20706.8 (17063.4-24605.4) | -0.02 (-0.06-0.01) |
| Saudi Arabia | 1317525 (1075306.8-1617974.1) | 19826.5 (16181.6-24347.8) | 3742573.9 (3067987.7-4590438.2) | 20205.8 (16563.8-24783.4) | 0.04 (0.03-0.05) |
| Democratic Republic of the Congo | 2435496.1 (1951359.2-3019499) | 17001.4 (13621.8-21078.2) | 6112960.5 (4901523.2-7569332.8) | 16940 (13582.9-20975.8) | -0.01 (-0.02--0.01) |
| San Marino | 2444 (1988.9-3024.3) | 26022.7 (21177.6-32202.1) | 2332.1 (1911.4-2883.9) | 26020.5 (21327.2-32178.3) | -0.02 (-0.05-0.02) |
| Morocco | 2211513.1 (1807416.7-2715705.3) | 21271.3 (17384.5-26120.8) | 3147777.2 (2578540.4-3851892.5) | 21440.6 (17563.3-26236.6) | 0.02 (0.02-0.03) |
| Malta | 35613.6 (29217.3-44085.4) | 25808.2 (21173-31947.5) | 34669.4 (28430.1-42960.2) | 25900.5 (21239.3-32094.3) | 0.02 (0.01-0.03) |
| Greenland | 6251.1 (5100.6-7637.2) | 23617.3 (19270.8-28854.2) | 4929.1 (4033.7-6025) | 24164.2 (19774.8-29536.9) | 0.03 (0-0.05) |
| Nicaragua | 288471 (233245.4-352387) | 19543.4 (15802-23873.6) | 549852.5 (446592.8-672346) | 19319.2 (15691.2-23623.1) | -0.04 (-0.04--0.04) |
| Panama | 194658.3 (157169.8-238036.2) | 19243.3 (15537.3-23531.5) | 316934.4 (257670.2-387133.4) | 19202.9 (15612.1-23456.2) | 0 (0-0.01) |
| Algeria | 2124923.9 (1729252.9-2609847.6) | 21029.7 (17113.8-25828.8) | 3670213.1 (3008831.4-4505290.8) | 21555.4 (17671-26459.8) | 0.09 (0.08-0.1) |
| Comoros | 21173.8 (17199.8-25925.6) | 12257.5 (9957-15008.3) | 38480.3 (31378.6-47096.6) | 12421.5 (10129.1-15202.9) | 0.04 (0.04-0.04) |
| Djibouti | 21144.9 (17175.9-25922.2) | 12060.4 (9796.7-14785.3) | 66900.7 (54739.2-81904.9) | 12371.5 (10122.5-15146.1) | 0.1 (0.09-0.11) |
| Nepal | 1567266.6 (1268267.1-1936596.8) | 21454.4 (17361.4-26510.2) | 2896832.2 (2346326.3-3591174.4) | 21605.9 (17499.9-26784.6) | 0.03 (0.02-0.03) |
| Seychelles | 6848.7 (5482.1-8381.8) | 21937.4 (17560.1-26848.1) | 8419.6 (6802.4-10305.8) | 21934.6 (17721.4-26848.2) | -0.03 (-0.05--0.02) |
| Somalia | 353978.5 (289056.8-433355.7) | 12209.3 (9970-14947.1) | 1002586.8 (814080.4-1228082.6) | 12092.9 (9819.2-14812.8) | 0.02 (-0.01-0.05) |
| Equatorial Guinea | 26060.1 (20881.1-32259.5) | 17258 (13828.3-21363.5) | 115468.7 (92543.9-143049.2) | 16599.7 (13304-20564.6) | -0.14 (-0.14--0.13) |
| Colombia | 2766537.8 (2278182.1-3392527.9) | 19684.7 (16209.9-24138.8) | 3977098.2 (3214327.7-4892760.6) | 19792.4 (15996.4-24349.2) | 0.03 (0.02-0.05) |
| Tokelau | 112.8 (91-139.6) | 19438.2 (15680.2-24069.4) | 96.4 (77.7-119.2) | 19487.2 (15697.5-24094) | 0 (0-0) |
| Mali | 604985 (487543.4-751071.6) | 20262.9 (16329.4-25155.8) | 1787086.4 (1432132.3-2221425.7) | 20063.6 (16078.5-24939.9) | 0 (0-0) |
| Grenada | 6604.3 (5288.5-8079.4) | 19808.6 (15862-24232.7) | 7915.7 (6365.7-9688.4) | 19584.1 (15749.4-23970) | -0.03 (-0.04--0.02) |
| Czechia | 689075.3 (556773.9-839567.9) | 18570.3 (15004.8-22626) | 554921.3 (451559.2-680926.1) | 18791.3 (15291.2-23058.3) | 0.08 (0.07-0.1) |
| Mauritania | 154710.1 (124579.9-192198.6) | 20122.9 (16203.9-24999) | 343726.8 (276499.5-426739.5) | 20136 (16197.7-24998.9) | 0 (0-0) |
| Tuvalu | 718.9 (579.3-885.9) | 19919.1 (16052.2-24546.1) | 960 (774.9-1190.1) | 19319.7 (15594.2-23950.5) | 0 (0-0) |
| Benin | 347299.2 (280545-431794.5) | 20395.8 (16475.5-25357.9) | 1054634.6 (849505.9-1311917.2) | 20116.8 (16204.1-25024.4) | 0 (0-0) |
| Hungary | 690705.4 (558883.7-841868.1) | 18688.3 (15121.6-22778.3) | 515858.5 (418501.2-632371.5) | 18748.1 (15209.8-22982.6) | 0.05 (0.04-0.07) |
| United Republic of Tanzania | 1177351.1 (957615-1448652.2) | 12151.5 (9883.6-14951.6) | 2959678.3 (2387653.2-3640094.5) | 12684.2 (10232.7-15600.3) | 0.29 (0.22-0.35) |
| Democratic People's Republic of Korea | 1370832.1 (1108867.2-1682538.3) | 16435.2 (13294.4-20172.3) | 1634799.4 (1321764.1-2005776.1) | 16253.2 (13141-19941.4) | -0.1 (-0.12--0.08) |
| United Arab Emirates | 184591 (149868-224785.1) | 19310.5 (15678.1-23515.3) | 764046.7 (621673.1-936789.2) | 19018.5 (15474.5-23318.3) | -0.07 (-0.11--0.03) |
| Niger | 560862 (451465-696924.9) | 20150.6 (16220.2-25039.1) | 1776590.7 (1422433.7-2220381.7) | 19922.7 (15951.2-24899.4) | 0 (0-0) |
| Afghanistan | 672110 (546369.5-823493.3) | 21339.9 (17347.6-26146.4) | 2555091.7 (2067272.1-3135094.8) | 20907.4 (16915.8-25653.4) | -0.07 (-0.1--0.05) |
| Viet Nam | 6318518.4 (5062032.8-7731608.9) | 22156.5 (17750.5-27111.7) | 8605913.3 (6962982.8-10524361.5) | 22417 (18137.4-27414.2) | 0.04 (0.02-0.06) |
| Palestine | 160948.3 (130497.5-197691.8) | 20970.6 (17003-25758) | 461682.6 (376406.4-566942.1) | 21142.8 (17237.6-25963.2) | 0.03 (0.03-0.03) |
| Uganda | 787566 (637669.3-965149) | 12278.3 (9941.4-15046.8) | 2111702.6 (1714786.5-2586102.8) | 12278.4 (9970.5-15036.8) | 0 (0-0) |
| Monaco | 2398.6 (1958.1-2980.8) | 26216.3 (21401.6-32579.5) | 2420.6 (1987.6-2998.4) | 25967 (21322-32165.7) | 0 (0-0) |
| Burkina Faso | 646935 (519859.3-801734.1) | 20292.8 (16306.8-25148.5) | 1753264.4 (1410534.2-2174247.5) | 20258.4 (16298.3-25122.7) | 0 (-0.01-0) |
| United Kingdom | 5339957 (4456659.4-6401338.2) | 25550.3 (21323.9-30628.7) | 5630049.6 (4683294.8-6706808.8) | 25883.5 (21530.9-30833.7) | 0.02 (0.01-0.04) |
| Nigeria | 7184400.7 (5884065.5-8623526.7) | 21041.4 (17233-25256.2) | 18869169.4 (15450279.6-22516719) | 20980 (17178.7-25035.6) | -0.01 (-0.03-0) |
| Barbados | 21746 (17534.5-26513.5) | 19923.1 (16064.7-24290.9) | 19662.2 (15976.1-23901.3) | 19905.4 (16173.7-24196.9) | 0 (0-0.01) |
| Cameroon | 767979.6 (618724.9-954648) | 20192.6 (16268.2-25100.7) | 2591360.9 (2090103-3220594) | 20099.2 (16211.3-24979.6) | -0.01 (-0.02--0.01) |
| Iraq | 1500730.1 (1221869.8-1841853.3) | 20886.5 (17005.4-25634.1) | 3674182.2 (3004285.2-4504911.4) | 21071.8 (17229.9-25836.1) | 0.04 (0.03-0.04) |
| Sudan | 1626710 (1329148.2-1998873.8) | 21346.4 (17441.6-26230) | 3938444 (3225884.3-4831928.9) | 21305.5 (17450.8-26138.9) | 0 (0-0) |
| Paraguay | 388606 (315720.8-470364.9) | 24794.7 (20144.3-30011.2) | 748093.3 (608474.1-907165.1) | 24451.1 (19887.7-29650.2) | -0.05 (-0.06--0.04) |
| Guinea | 419073.9 (338854.7-521104.8) | 20398.7 (16494-25365.1) | 1049537.9 (846127.4-1303018.2) | 20312.1 (16375.4-25217.8) | 0.01 (0-0.01) |
| Angola | 664777.5 (533195-822621.4) | 16990.5 (13627.5-21024.7) | 2083961 (1671800-2581472) | 17129.2 (13741.4-21218.5) | 0.04 (0.03-0.04) |
| Belize | 14515.1 (11604.3-17748.6) | 19838 (15859.8-24257.3) | 37682.9 (30314.4-46098.8) | 19959.8 (16056.8-24417.5) | 0.02 (0.02-0.03) |
| Jordan | 317224.1 (256809.2-388567.4) | 20639.8 (16709-25281.7) | 1115410.6 (912383.3-1364221.6) | 20778.1 (16996-25413) | 0.02 (0-0.04) |
| Nauru | 790.6 (636.5-979.4) | 19554.8 (15741.9-24223.5) | 908.4 (731.4-1125.4) | 19518.4 (15715.9-24180.2) | 0 (-0.01-0) |
| Niue | 155.5 (125.4-192.5) | 19315.3 (15579.2-23918.9) | 110.8 (89.2-136.7) | 19516.6 (15709.8-24078.1) | 0 (0-0) |
| Gabon | 65338.1 (52398.1-80940.3) | 16987.1 (13622.9-21043.5) | 129643.3 (104114.6-160146) | 17296 (13890.1-21365.4) | 0.05 (0.05-0.06) |
| Guinea-Bissau | 74913.8 (60249.9-92978.8) | 20201.1 (16246.9-25072.5) | 170591.6 (137805.4-212106.1) | 20217.5 (16331.9-25137.6) | 0 (0-0) |
| South Sudan | 279320.8 (226733.2-342356.6) | 12099.8 (9821.8-14830.5) | 440889.2 (357190-538438.1) | 12250 (9924.4-14960.4) | 0 (0-0) |
| Mozambique | 594776 (485160.6-726940.3) | 12539.9 (10228.8-15326.3) | 1488044.1 (1209182-1821972.9) | 12376 (10056.7-15153.3) | -0.04 (-0.05--0.03) |
| Saint Lucia | 11261.4 (8995.7-13772.2) | 20017 (15989.7-24480) | 13045.5 (10579.3-15881.4) | 19737.7 (16006.3-24028.4) | -0.05 (-0.05--0.05) |
| Eswatini | 51889.2 (41513.1-64410.7) | 17218 (13775-21373) | 87742.5 (70345.2-108176.4) | 17233.7 (13816.7-21247.2) | 0.01 (0-0.02) |
| Liberia | 186325.7 (150818.8-231676.9) | 20196 (16347.4-25111.6) | 450499.8 (362984.3-558736.9) | 20065.1 (16167.2-24885.9) | -0.04 (-0.06--0.03) |
| Taiwan (Province of China) | 1605816 (1342645.6-1910079.8) | 17404.4 (14552.1-20702.1) | 1368040.8 (1114001-1686216) | 18132.3 (14765.2-22349.5) | 0.16 (0.14-0.19) |
| Bosnia and Herzegovina | 350492.9 (282954.2-428504.6) | 18455.6 (14899.3-22563.4) | 188415.1 (152765.3-230501.1) | 18724 (15181.3-22906.4) | 0.01 (-0.01-0.03) |
| Rwanda | 338593.6 (275654.8-414116.2) | 12354.9 (10058.3-15110.6) | 703847.5 (574284.7-861238.4) | 12410.5 (10126-15185.7) | 0.01 (-0.02-0.04) |
| Republic of Korea | 3797551 (3015171.4-4599422.2) | 18042.2 (14325.1-21851.9) | 2790036 (2252722.6-3437796.3) | 17436.1 (14078.2-21484.2) | -0.29 (-0.35--0.23) |
| Bangladesh | 8433147.3 (6792397.9-10405544.7) | 19979 (16091.9-24651.8) | 14021125.2 (11311982.3-17270298.4) | 20375.4 (16438.5-25097) | 0.06 (0.06-0.07) |
| Bahrain | 51782.3 (42020.9-63367.1) | 20200.7 (16392.7-24720) | 138749.6 (112927.3-169738.7) | 19721.1 (16050.8-24125.7) | -0.15 (-0.18--0.12) |
| Singapore | 183548 (152848.9-228732.2) | 12161.4 (10127.4-15155.2) | 276881.4 (225053.8-345065.7) | 14393.1 (11699-17937.5) | 0.6 (0.44-0.76) |
| Armenia | 270932 (218369.5-334611.2) | 18851.2 (15194-23282) | 204941.4 (166079.5-253579.8) | 19063.5 (15448.6-23587.8) | 0.03 (0.01-0.06) |
| Belarus | 700733.5 (573010-861829.2) | 17764.9 (14526.9-21849) | 526648.9 (430773.4-645763.2) | 17951.8 (14683.7-22012) | 0.04 (0.01-0.06) |
| Estonia | 100530.4 (82213.2-123442.3) | 17699.5 (14474.5-21733.4) | 70168.9 (57272.1-86229.4) | 17743.9 (14482.6-21805.2) | 0.01 (-0.01-0.03) |
| France | 5685345 (4633511.7-6985041.6) | 25841.8 (21060.8-31749.3) | 5073408.1 (4177699.9-6176186.2) | 25537.3 (21028.7-31088.3) | -0.06 (-0.08--0.04) |
| Bulgaria | 555142.6 (448434.7-676723.1) | 18648.3 (15063.8-22732.5) | 356803.2 (290276.4-437729.6) | 18783 (15280.9-23043.2) | 0.05 (0.04-0.06) |
| Croatia | 339021.1 (274258.9-414625.4) | 18683.9 (15114.7-22850.5) | 233567 (191418.7-285545.9) | 18712.5 (15335.8-22876.9) | -0.06 (-0.16-0.04) |
| Antigua and Barbuda | 5166.2 (4148.2-6320.7) | 20032.7 (16085.3-24509.5) | 6843.3 (5550-8330.3) | 19894.2 (16134.5-24217.2) | -0.04 (-0.06--0.02) |
| Guam | 12284.3 (9930.1-15236.7) | 19367.4 (15655.7-24022.1) | 10829 (8747.5-13377.7) | 19533.8 (15779.2-24131.3) | -0.01 (-0.03-0.01) |
| Zimbabwe | 675142.9 (540049.3-838024.5) | 17029.2 (13621.7-21137.5) | 1090534.5 (875288.4-1347035.3) | 17207.3 (13811-21254.5) | 0.05 (0.04-0.06) |
| United States Virgin Islands | 7986 (6464-9729.6) | 20160.1 (16318-24561.7) | 4616.8 (3762.4-5605.1) | 19962.1 (16267.8-24235.3) | 0 (0-0) |
| Bahamas | 23545.3 (18894.1-28844.9) | 19951.1 (16009.9-24441.7) | 30912.5 (24962.5-37677) | 19987.1 (16139.9-24360.7) | 0.01 (0.01-0.01) |
| Germany | 8159357.6 (6698420.5-10048403.5) | 27464.8 (22547.2-33823.4) | 7106517.6 (5878505.4-8694972.7) | 28090.7 (23236.6-34369.6) | 0 (-0.08-0.08) |
| Malaysia | 1465149.1 (1180221.8-1791058.1) | 19740.8 (15901.8-24132) | 2819797.7 (2291312.8-3467622.5) | 20282.7 (16481.3-24942.4) | 0.1 (0.08-0.12) |
| Spain | 3939342 (3225724.1-4906797.9) | 26563.7 (21751.6-33087.4) | 3332103.8 (2747039.1-4081918.6) | 26837 (22124.9-32876.1) | 0.04 (0.03-0.05) |
| Micronesia (Federated States of) | 7759.5 (6257.2-9626.5) | 19366.8 (15617.4-24026.6) | 8237 (6644.8-10211.7) | 19396.7 (15647.4-24046.7) | 0 (0-0.01) |
| Greece | 1004689.8 (825735.4-1243697.3) | 26725.3 (21965-33083) | 747429.8 (614979.2-923703.9) | 26849 (22091.1-33181) | 0.03 (0.02-0.04) |
| Saint Vincent and the Grenadines | 9087.7 (7261.1-11112.3) | 19784.9 (15808.3-24192.7) | 8162.9 (6598.9-9957.1) | 19754.5 (15969.5-24096.7) | 0.01 (0-0.01) |
| Suriname | 32090.9 (25642.3-39287.8) | 19706.4 (15746.4-24125.9) | 42729.8 (34555.7-52091.5) | 19906.5 (16098.5-24267.8) | 0.04 (0.03-0.04) |
| Sweden | 767331.4 (642081.3-904233.9) | 26140.3 (21873.4-30804) | 850426.7 (711710.9-1003447.7) | 26235.7 (21956.3-30956.4) | 0.01 (0-0.02) |
| Syrian Arab Republic | 1006450.8 (818222-1236572.1) | 20978.9 (17055.4-25775.6) | 1104901.5 (903023.9-1350064) | 21723.7 (17754.5-26543.8) | 0.14 (0.11-0.18) |
| Guatemala | 576915.5 (467022.6-706035.5) | 19530.3 (15810.1-23901.4) | 1329586.1 (1072966.1-1625648.1) | 19533.4 (15763.3-23883) | 0 (0-0.01) |
| Tunisia | 729043.1 (594702.2-895604.6) | 21194.3 (17288.9-26036.5) | 945318.8 (776430.2-1161031.7) | 21722.7 (17841.8-26679.6) | 0.09 (0.08-0.1) |
| Honduras | 336105.4 (272040.2-411311.9) | 19468 (15757.2-23824.1) | 860604.8 (693813.2-1052139.9) | 19578.6 (15784.1-23936) | 0.03 (0.03-0.03) |
| Turkey | 4957077.2 (4041899.7-6064349.2) | 20724.5 (16898.4-25353.8) | 6676476.1 (5492914.1-8153332.9) | 20955.9 (17241-25591.4) | 0.05 (0.01-0.09) |
| Mexico | 6917584.1 (5735666.6-8296360.3) | 19397.7 (16083.4-23263.9) | 10119349 (8352985.9-12179388.9) | 19644.8 (16215.7-23644) | 0.05 (0.04-0.06) |
| Switzerland | 612843.7 (501533.2-749455.2) | 23258.4 (19034-28443) | 656532.3 (535689.5-804911.8) | 23645.6 (19293.4-28989.6) | 0.05 (0.05-0.05) |
| Cabo Verde | 26349.1 (21135.8-32850.2) | 20150.8 (16163.9-25122.6) | 50428.1 (40811.7-62417.4) | 20121.4 (16284.3-24905.3) | -0.02 (-0.03-0) |
| Zambia | 408005.7 (327953.7-507706.9) | 13446.6 (10808.4-16732.5) | 1103318.8 (892217.2-1367035.3) | 13633.2 (11024.7-16891.8) | -0.02 (-0.04-0.01) |
| Chad | 424189.4 (340935.4-526084.8) | 20208.4 (16242.2-25062.7) | 1262271.4 (1010677-1569046) | 20058.7 (16060.6-24933.6) | 0 (0-0) |
| Northern Mariana Islands | 4654.5 (3772.6-5724.7) | 19874.5 (16109-24444.3) | 3219.9 (2596.2-3960.2) | 19528.8 (15746-24018.7) | 0 (0-0) |
| Palau | 1357.4 (1095.2-1681) | 19459.3 (15700.6-24098.7) | 1124.8 (911.7-1386.4) | 19097.4 (15480.3-23538.6) | -0.08 (-0.11--0.06) |
| Sao Tome and Principe | 8567.9 (6860.8-10671.6) | 19958.8 (15982.1-24859.3) | 18215.1 (14680.5-22627.1) | 20041.3 (16152.3-24895.6) | 0.02 (0.02-0.02) |
| Pakistan | 8176169.8 (6705831.2-9813867.6) | 20037.2 (16433.9-24050.7) | 20048658.5 (16522571-23989135.1) | 20270.4 (16705.3-24254.4) | 0.01 (-0.01-0.03) |
| Egypt | 4684923.4 (3840248.6-5724820) | 21370.4 (17517.4-26114) | 9460165.5 (8059011.6-11144061.6) | 22411.2 (19091.8-26400.3) | 0.21 (0.17-0.25) |
| North Macedonia | 146947 (118575.1-179396.1) | 18504.8 (14931.9-22591) | 143220.6 (116553.5-175583.1) | 18727.3 (15240.4-22959) | 0.03 (0.02-0.04) |
| Senegal | 556175.2 (446961.5-690893) | 20155.3 (16197.5-25037.3) | 1290485.4 (1039093.9-1604667.1) | 20014.2 (16115.3-24886.8) | 0 (0-0) |
| Iran (Islamic Republic of) | 4856906.5 (4028964.5-5783176) | 22363.2 (18551.1-26628.2) | 8088631.1 (6845799.9-9576529.6) | 23308.1 (19726.7-27595.6) | 0.13 (0.06-0.2) |
| Eritrea | 159244.4 (129581.5-194832.3) | 12288.3 (9999.4-15034.5) | 343960.2 (280149.6-421067.8) | 12284.5 (10005.5-15038.4) | 0 (-0.01-0.01) |
| Kyrgyzstan | 335124.9 (269050.8-414021) | 18580.6 (14917.2-22955) | 510246.1 (410966.3-629129.2) | 18748.4 (15100.5-23116.7) | 0.02 (-0.01-0.04) |
| Ethiopia | 2061874.2 (1695207.8-2469317.4) | 11285.8 (9278.8-13515.9) | 5217630.6 (4269592.2-6226635.1) | 11255.9 (9210.7-13432.6) | 0.02 (0-0.03) |
| Republic of Moldova | 311394.3 (254726.5-382675) | 17867.3 (14615.8-21957.3) | 222891.3 (182627.6-273430.9) | 17971.3 (14724.9-22046.2) | 0.02 (-0.01-0.06) |
