# Supplementary material for: Global, regional, and national burden and trends of migraine among youths and young adults aged 15–39 years from 1990 to 2021: findings from the global burden of disease study 2021
Source: J Headache Pain. 2024 Aug 12;25(1):131. doi: 10.1186/s10194-024-01832-0 (PMC11318134; doi:10.1186/s10194-024-01832-0)
Supplement: Supplementary file 27 — Supplementary Material 27: Table S5 Incidence of Migraine Between 1990 and 2021 in 15 to 39 years at the 204 Countries Level [file 10194_2024_1832_MOESM27_ESM.docx]

| **TableS5 Incidence of Migraine Between 1990 and 2021 in 15 to 39 years at the 204 Countries Level** | | | | | |
| --- | --- | --- | --- | --- | --- |
| **Location** | **1990** | | **2021** | | **EAPC_95%CI** |
|  | **Number** | **ASR** | **Number** | **ASR** |  |
| Maldives | 1395.8 (1087.2-1795.5) | 1717.6 (1337.8-2209.4) | 4013.6 (3173.5-5207.3) | 1542.7 (1219.8-2001.5) | -0.35 (-0.42--0.29) |
| Myanmar | 293393.6 (230133.2-376967.9) | 1709 (1340.5-2195.8) | 381807.8 (303439.1-489535.5) | 1698.3 (1349.7-2177.5) | -0.02 (-0.03--0.02) |
| Papua New Guinea | 25317.3 (19786.6-32742.3) | 1529.6 (1195.4-1978.2) | 65163.8 (51526.5-83807.5) | 1522.7 (1204-1958.3) | -0.01 (-0.02--0.01) |
| Philippines | 466918.4 (385911.7-570137.8) | 1801.4 (1488.9-2199.7) | 840566.8 (696814.6-1026355.7) | 1779 (1474.7-2172.2) | -0.04 (-0.04--0.03) |
| Sri Lanka | 125206.5 (99229.3-160531.8) | 1693.2 (1341.9-2170.9) | 136573.9 (109095.5-175377.9) | 1693.5 (1352.8-2174.7) | -0.01 (-0.01--0.01) |
| Samoa | 1024.1 (802.6-1337) | 1529.4 (1198.6-1996.6) | 1228.8 (969.2-1594.1) | 1529.3 (1206.2-1983.9) | 0.01 (0.01-0.02) |
| Romania | 123048.8 (98259.8-159756.6) | 1416.5 (1131.1-1839) | 75748.8 (60365.4-98289.8) | 1405.6 (1120.1-1823.8) | -0.03 (-0.04--0.03) |
| Mongolia | 12723.5 (9975.8-16478.9) | 1439.8 (1128.9-1864.8) | 17916.6 (14281.1-22908.1) | 1419.6 (1131.5-1815) | -0.05 (-0.06--0.05) |
| Serbia | 50501.7 (40335-65229.4) | 1406.3 (1123.2-1816.4) | 41287 (32888.4-53422.4) | 1392.8 (1109.5-1802.2) | -0.03 (-0.04--0.01) |
| Montenegro | 3521.4 (2822.4-4536) | 1402.8 (1124.4-1807) | 2893 (2303.5-3734) | 1406.2 (1119.7-1815) | 0 (-0.01-0.01) |
| Belgium | 68725.2 (53981.6-87337) | 1847.2 (1450.9-2347.4) | 66180 (51845.6-83858) | 1889.5 (1480.2-2394.2) | 0.11 (0.08-0.14) |
| Solomon Islands | 1981.5 (1553-2592) | 1544.8 (1210.8-2020.8) | 4198.1 (3314.8-5453.8) | 1534.9 (1211.9-1993.9) | -0.02 (-0.03--0.02) |
| Central African Republic | 13794.7 (10735.2-17986.7) | 1324.7 (1030.9-1727.3) | 29032.9 (22791.1-37703.9) | 1329.8 (1043.9-1727) | 0 (0-0) |
| Andorra | 431.9 (343.5-544.1) | 1727.1 (1373.4-2175.6) | 447.9 (360.9-564.2) | 1756.7 (1415.3-2213) | 0.07 (0.05-0.1) |
| Cyprus | 5494.2 (4382.4-6929.7) | 1786.4 (1424.9-2253.2) | 8687.8 (6933.7-11014.2) | 1731.5 (1381.9-2195.2) | -0.11 (-0.14--0.08) |
| Cuba | 61781.1 (48101.3-79739.6) | 1266.1 (985.7-1634.1) | 45137.4 (35204.2-58986.9) | 1258.8 (981.8-1645.1) | -0.02 (-0.03-0) |
| Argentina | 156218.6 (124507.2-201292.3) | 1278.9 (1019.3-1647.9) | 228765.3 (182234.6-292699) | 1305.5 (1040-1670.4) | 0.1 (0.07-0.12) |
| Yemen | 68287.3 (53269-88828.7) | 1485.5 (1158.8-1932.4) | 202785.9 (158215.6-261689.8) | 1473.8 (1149.8-1901.9) | 0 (0-0) |
| Russian Federation | 832230.7 (688986.1-1017737.9) | 1430.6 (1184.4-1749.5) | 662958.3 (549288.7-809746) | 1426.5 (1181.9-1742.3) | -0.02 (-0.05-0) |
| Tonga | 572.1 (449-748.9) | 1550.6 (1216.8-2029.7) | 601.5 (474.5-784) | 1546.5 (1219.9-2015.6) | 0 (-0.01-0.01) |
| Thailand | 468277.4 (370957.1-599769.2) | 1805.8 (1430.5-2312.9) | 368520.5 (295331.4-467803.5) | 1738.3 (1393.1-2206.7) | -0.13 (-0.16--0.09) |
| Dominica | 366.4 (284.6-475.7) | 1254.7 (974.4-1628.7) | 327.1 (257.4-425.9) | 1263 (993.7-1644.3) | 0.03 (0.01-0.04) |
| Slovakia | 28921.7 (23111.9-37434.3) | 1412.8 (1129-1828.7) | 24007.9 (19132.4-30730) | 1402.9 (1118-1795.7) | -0.04 (-0.05--0.04) |
| Turkmenistan | 22097.7 (17384.4-28378.8) | 1439.5 (1132.5-1848.7) | 29139.9 (23153-37570) | 1401 (1113.2-1806.4) | -0.1 (-0.1--0.09) |
| Botswana | 6894.7 (5375.4-8942.9) | 1339.4 (1044.2-1737.3) | 13893.1 (11063.5-17959.1) | 1304.1 (1038.5-1685.7) | 0 (0-0) |
| Denmark | 31656.6 (25549.9-40181.6) | 1659.2 (1339.1-2106) | 30798.1 (24756.7-39164.4) | 1688.8 (1357.5-2147.6) | 0.13 (0.11-0.15) |
| Slovenia | 10798.9 (8600.2-13953) | 1409.2 (1122.3-1820.7) | 7962.2 (6355.6-10205.1) | 1398.4 (1116.2-1792.2) | -0.05 (-0.06--0.04) |
| Coted'Ivoire | 68524.6 (53723.7-88637.3) | 1448.7 (1135.8-1873.9) | 161875.7 (127402.7-208626.6) | 1443.8 (1136.3-1860.8) | 0 (0-0) |
| Kenya | 93921.1 (76245.5-115230.8) | 1072.5 (870.6-1315.8) | 231097 (189827.4-280574) | 1067.3 (876.7-1295.9) | 0 (0-0) |
| Tajikistan | 30493.3 (23958-39415.3) | 1442 (1132.9-1863.9) | 59338.8 (47184.7-76289.1) | 1422.6 (1131.2-1829) | -0.04 (-0.06--0.03) |
| Brunei Darussalam | 1451.9 (1157.6-1868.8) | 1177.7 (938.9-1515.8) | 2356.9 (1882.3-3042.2) | 1155.1 (922.5-1491) | -0.07 (-0.11--0.03) |
| Finland | 32086.2 (25685.6-40358.5) | 1767.4 (1414.8-2223.1) | 29471 (23501.9-37187) | 1769 (1410.7-2232.1) | 0 (-0.02-0.02) |
| Japan | 541449.5 (444516-656458) | 1208.2 (991.9-1464.8) | 393698.7 (325537.5-481583.5) | 1214.7 (1004.4-1485.9) | 0.04 (-0.01-0.09) |
| Cambodia | 66869.9 (52432.2-85575.7) | 1736.8 (1361.8-2222.6) | 121918 (96559.3-155830.1) | 1683.2 (1333.1-2151.4) | -0.09 (-0.09--0.08) |
| Uzbekistan | 123435.5 (97167.1-158360.1) | 1438.1 (1132-1844.9) | 195027.5 (155545.1-249320.1) | 1419.7 (1132.3-1814.9) | -0.04 (-0.06--0.03) |
| Kuwait | 11859.8 (9296.8-15455.4) | 1403.3 (1100.1-1828.8) | 31182.6 (24524.4-40971.2) | 1469 (1155.3-1930.1) | 0 (0-0) |
| Madagascar | 45793.8 (36197.4-60193.5) | 1011 (799.1-1328.9) | 118609.4 (93842.5-155583.2) | 1012.8 (801.3-1328.5) | 0.01 (0.01-0.01) |
| Austria | 52248.7 (42003-65885.6) | 1740.5 (1399.2-2194.7) | 48349.3 (39000.9-61603.8) | 1713 (1381.8-2182.6) | 0.05 (0.02-0.09) |
| Vanuatu | 902.8 (709.5-1168.4) | 1541.1 (1211.1-1994.5) | 1915.6 (1509-2469.9) | 1536.9 (1210.7-1981.7) | -0.01 (-0.01-0) |
| Lesotho | 7298.3 (5747.6-9503.5) | 1352.6 (1065.2-1761.3) | 10942.6 (8578.7-14275.3) | 1315.5 (1031.3-1716.1) | 0 (0-0) |
| Guyana | 4338.2 (3364.7-5631.5) | 1274.6 (988.6-1654.6) | 3955.6 (3080.9-5127) | 1273 (991.5-1649.9) | 0.01 (0-0.02) |
| Indonesia | 1407197.9 (1163044.1-1719913.8) | 1803.1 (1490.2-2203.8) | 2010613.8 (1660217.8-2454202.3) | 1765.6 (1457.9-2155.2) | -0.07 (-0.07--0.07) |
| Chile | 74804.5 (60005.2-95071.1) | 1306.1 (1047.7-1660) | 91279.8 (72843.2-116953.3) | 1290.1 (1029.5-1652.9) | 0.03 (0-0.07) |
| Lao People's Democratic Republic | 26603.5 (20967.4-34055.1) | 1721.9 (1357.1-2204.2) | 54099.3 (42939.1-69178.1) | 1686.5 (1338.6-2156.5) | -0.06 (-0.07--0.06) |
| Sierra Leone | 23422.6 (18419.5-30172.1) | 1466.3 (1153.1-1888.8) | 54385.9 (42679.6-70084.8) | 1458.2 (1144.3-1879.1) | 0 (0-0) |
| Ukraine | 265471.6 (219642.9-324400) | 1397.6 (1156.4-1707.9) | 190146 (156849.6-229953.6) | 1379.4 (1137.8-1668.1) | -0.06 (-0.07--0.05) |
| Bolivia (Plurinational State of) | 24613.1 (19106.5-32381.3) | 998.1 (774.8-1313.1) | 48833.6 (38115.3-64290.7) | 993.8 (775.7-1308.3) | -0.02 (-0.03--0.02) |
| Uruguay | 15006 (11936.4-19524) | 1321.2 (1050.9-1718.9) | 15699.9 (12542.6-20321.7) | 1312.4 (1048.5-1698.8) | -0.02 (-0.02--0.01) |
| Haiti | 31478.5 (24576.1-40731.9) | 1293.1 (1009.6-1673.3) | 70441.1 (55305.7-91470) | 1283.2 (1007.5-1666.3) | -0.03 (-0.03--0.03) |
| Ecuador | 42167.1 (33031-54691.6) | 1021.9 (800.5-1325.4) | 76643.2 (58544-101039.1) | 1049.3 (801.5-1383.3) | 0.12 (0.1-0.14) |
| Jamaica | 12574.6 (9732.8-16313.7) | 1279.5 (990.4-1660) | 15172.6 (11893.7-19693.1) | 1271.5 (996.8-1650.4) | -0.02 (-0.03--0.02) |
| Venezuela (Bolivarian Republic of) | 100508.2 (77994.5-130127.8) | 1254.9 (973.8-1624.7) | 121547.7 (95482.9-157350.4) | 1298.2 (1019.8-1680.6) | 0.08 (0.06-0.1) |
| American Samoa | 310.3 (241.6-399.8) | 1533.9 (1194.2-1976.3) | 267.9 (213.7-351.1) | 1533.1 (1223.1-2009) | 0 (0-0) |
| Iceland | 1864.9 (1487.1-2355.1) | 1795.2 (1431.5-2267.1) | 2117.9 (1687.4-2662.8) | 1769.8 (1410-2225.1) | -0.04 (-0.05--0.02) |
| Puerto Rico | 18195.9 (14258.5-23529.4) | 1286.3 (1008-1663.4) | 13220.6 (10425.7-17237.5) | 1278.3 (1008-1666.6) | 0 (0-0) |
| Ireland | 25148.6 (19954.2-32294.2) | 1833.3 (1454.7-2354.3) | 28340.1 (22750.5-35997.2) | 1811.2 (1454-2300.6) | -0.1 (-0.13--0.06) |
| Latvia | 12624.2 (10108.5-16389.5) | 1323.3 (1059.6-1718) | 7049.3 (5625-9207.9) | 1308.8 (1044.4-1709.6) | -0.05 (-0.06--0.04) |
| Albania | 20079.1 (15892.1-25838.5) | 1413.5 (1118.8-1819) | 13175.9 (10455.5-16937.5) | 1389.8 (1102.8-1786.5) | -0.06 (-0.1--0.03) |
| Lithuania | 17999 (14436.9-23127.6) | 1291.8 (1036.1-1659.9) | 10288.1 (8265.5-13295.6) | 1277.9 (1026.6-1651.4) | -0.09 (-0.11--0.07) |
| Canada | 189492.4 (153065.1-237084.1) | 1704.6 (1376.9-2132.7) | 200903.3 (161243.3-252814.3) | 1693.7 (1359.4-2131.4) | 0.01 (-0.03-0.04) |
| Netherlands | 102292.3 (82370.5-127595.9) | 1696.4 (1366.1-2116.1) | 93609.9 (74970.7-120029.1) | 1772.8 (1419.8-2273.1) | 0.29 (0.21-0.36) |
| Poland | 215072.2 (177736.6-261763.3) | 1488.9 (1230.4-1812.1) | 179094.5 (147055.8-218537.4) | 1480.3 (1215.5-1806.3) | -0.05 (-0.06--0.04) |
| Australia | 100668.3 (81363.2-128784.4) | 1486.4 (1201.4-1901.6) | 127407.4 (102309-163017.8) | 1469.6 (1180.1-1880.4) | -0.02 (-0.03--0.01) |
| Italy | 419991.8 (348630.3-505217.4) | 1967.3 (1633.1-2366.5) | 306840.9 (255514.4-368731.3) | 1942.6 (1617.7-2334.5) | 0.12 (0.05-0.18) |
| Brazil | 861450 (695578.1-1066669.4) | 1372.9 (1108.6-1700) | 1156887.6 (945039.3-1424773.8) | 1357.1 (1108.5-1671.3) | -0.14 (-0.22--0.07) |
| Lebanon | 17310.1 (13648-22068.8) | 1501.6 (1183.9-1914.4) | 33953.6 (26627.3-43646.9) | 1463.3 (1147.6-1881) | -0.08 (-0.09--0.06) |
| Fiji | 4927.5 (3887.2-6336.7) | 1528.1 (1205.5-1965.1) | 5415.3 (4302.3-6938.8) | 1518 (1206-1945.1) | -0.03 (-0.03--0.03) |
| Israel | 35078 (27891.9-45046.3) | 1835.6 (1459.6-2357.3) | 60346.6 (48120.6-77184.7) | 1816 (1448.1-2322.7) | -0.07 (-0.08--0.05) |
| New Zealand | 21722.5 (18103.3-26675) | 1572.4 (1310.4-1930.9) | 27693.6 (23110.2-33960.3) | 1537.3 (1282.9-1885.2) | -0.01 (-0.03-0.01) |
| Congo | 12554 (9794-16283.3) | 1325.2 (1033.8-1718.9) | 29214.1 (23072-38012.4) | 1318.6 (1041.3-1715.7) | -0.02 (-0.02--0.01) |
| Saint Kitts and Nevis | 219.9 (170.5-283.4) | 1272.3 (986.4-1639.8) | 288.7 (226.3-376.3) | 1270.3 (995.7-1655.6) | 0 (0-0) |
| Bermuda | 328 (254.7-427) | 1275.3 (990.5-1660.6) | 224.5 (175.4-292.6) | 1282 (1001.8-1671.3) | 0 (0-0) |
| Togo | 20143.7 (15750.7-25936.2) | 1469.4 (1148.9-1891.9) | 49024.6 (38566.5-63213.9) | 1457.1 (1146.3-1878.8) | 0 (0-0) |
| Dominican Republic | 39439.6 (30580.7-51120.7) | 1283.8 (995.5-1664.1) | 57539.7 (45063.4-74824) | 1265.4 (991-1645.5) | -0.06 (-0.07--0.06) |
| Peru | 75063.6 (59098.2-95776) | 846.1 (666.2-1079.6) | 138877.1 (109325.4-182354.4) | 934.5 (735.6-1227.1) | 0.35 (0.26-0.45) |
| Kiribati | 469.7 (366.6-605.9) | 1538.5 (1200.7-1984.4) | 763.2 (603-981.1) | 1534.9 (1212.6-1973.2) | -0.01 (-0.01-0) |
| United States of America | 1886999.5 (1566734.4-2240572.7) | 1846.9 (1533.5-2193) | 2050277 (1715839.5-2471429) | 1842.1 (1541.6-2220.5) | 0.01 (-0.01-0.03) |
| Norway | 29972.8 (25224.5-36085.9) | 1873.5 (1576.7-2255.6) | 32680.8 (27257.6-39585.9) | 1841.4 (1535.9-2230.5) | -0.18 (-0.23--0.12) |
| Costa Rica | 16258.9 (12580-21171) | 1265.7 (979.3-1648.1) | 24358.3 (19211.9-31911.6) | 1279.8 (1009.4-1676.7) | 0.04 (0.03-0.06) |
| Trinidad and Tobago | 6362.3 (4970.3-8229.4) | 1269 (991.4-1641.4) | 6329.7 (4969.7-8303.9) | 1271.6 (998.4-1668.2) | -0.01 (-0.02-0) |
| Azerbaijan | 45730.1 (36067.9-58501.6) | 1438.9 (1134.9-1840.8) | 59640.4 (47540.1-75918.4) | 1408 (1122.3-1792.3) | -0.08 (-0.1--0.06) |
| Marshall Islands | 263.6 (207.7-343.7) | 1536.7 (1211-2003.9) | 360.6 (285.6-465.9) | 1520.1 (1204.1-1964.2) | -0.06 (-0.07--0.05) |
| El Salvador | 26675.6 (20633.2-34795.2) | 1276.8 (987.6-1665.4) | 33401.7 (25825.1-43563.3) | 1288.7 (996.4-1680.7) | 0.02 (0.01-0.03) |
| Portugal | 69151.2 (55006.6-88442.8) | 1826.4 (1452.9-2336) | 53030.2 (42575.1-66797.5) | 1796.6 (1442.4-2263.1) | -0.07 (-0.1--0.04) |
| Malawi | 37940.2 (29849.5-49900.2) | 1015.4 (798.9-1335.5) | 83193.8 (65740.1-108370.5) | 1016.6 (803.4-1324.3) | 0 (0-0) |
| Namibia | 7433.8 (5792.7-9645) | 1329.3 (1035.9-1724.8) | 13756.3 (10805-17822) | 1316.3 (1033.9-1705.3) | 0 (0-0) |
| China | 6814464 (5574886.1-8344913.6) | 1243.2 (1017.1-1522.4) | 5988037.3 (4916519.8-7253657.7) | 1297.7 (1065.5-1571.9) | 0.17 (0.13-0.21) |
| Gambia | 5518.5 (4319.5-7132.3) | 1463.7 (1145.7-1891.7) | 14633.1 (11470.1-18804.3) | 1463.1 (1146.9-1880.2) | 0 (0-0) |
| Luxembourg | 2557.8 (2055.3-3246.4) | 1733.2 (1392.7-2199.9) | 3810.1 (3065.6-4843.1) | 1727.1 (1389.6-2195.4) | 0.01 (0-0.03) |
| Libya | 24179.6 (18723.7-31564.3) | 1439.1 (1114.4-1878.6) | 43722.2 (34256.8-56319.7) | 1457.2 (1141.7-1877) | -0.01 (-0.03-0.01) |
| South Africa | 220298.3 (181446.8-266922.3) | 1400 (1153.1-1696.3) | 332873.1 (276061.3-407120.4) | 1372.8 (1138.5-1679.1) | 0 (0-0) |
| Ghana | 83886.6 (65770.3-107868.4) | 1461.4 (1145.8-1879.2) | 208180.4 (164248.5-267080.7) | 1455.6 (1148.5-1867.5) | 0 (0-0) |
| Oman | 10939.8 (8543.8-14116.3) | 1318.5 (1029.8-1701.4) | 30390.2 (23777.9-39192.1) | 1313.1 (1027.4-1693.5) | 0 (0-0) |
| Georgia | 30536.4 (24343-38972) | 1434.3 (1143.4-1830.6) | 15989.2 (12794.9-20509.2) | 1409.1 (1127.6-1807.5) | -0.07 (-0.09--0.05) |
| Qatar | 2948.4 (2313.3-3820.1) | 1246.9 (978.3-1615.6) | 20197.7 (15553.5-26567.2) | 1222.2 (941.2-1607.7) | -0.26 (-0.36--0.17) |
| Timor-Leste | 5373 (4214-6901.4) | 1688 (1323.9-2168.2) | 9809.2 (7680.6-12605.2) | 1716.9 (1344.3-2206.3) | 0.07 (0.07-0.08) |
| Cook Islands | 118 (92.9-152.7) | 1529.2 (1203.1-1979) | 91.4 (72.2-117.3) | 1550.3 (1225.8-1990.9) | 0 (0-0) |
| Mauritius | 8354.2 (6608-10673.4) | 1680.5 (1329.3-2147.1) | 7637.3 (6052.9-9774.3) | 1677.8 (1329.7-2147.2) | 0 (0-0) |
| Kazakhstan | 96883.6 (77098.2-123941.1) | 1427.1 (1135.6-1825.6) | 99292.9 (79177.8-126882.6) | 1424.7 (1136.1-1820.6) | -0.02 (-0.03-0) |
| Bhutan | 4006.4 (3113.2-5174.3) | 1486.8 (1155.4-1920.3) | 5170.6 (4102.4-6694.6) | 1491.9 (1183.6-1931.6) | 0 (0-0) |
| Burundi | 21043.2 (16631.9-27498.3) | 1015.1 (802.3-1326.5) | 53114.9 (42074.2-69540.8) | 1007.4 (798-1319) | 0 (0-0) |
| India | 5329707.3 (4419079.8-6442286) | 1562.8 (1295.8-1889.1) | 9537413.5 (7922632.7-11557511.3) | 1564.9 (1300-1896.4) | -0.02 (-0.06-0.02) |
| Saudi Arabia | 91265 (70501.4-117927.7) | 1373.4 (1060.9-1774.6) | 253104.1 (199976.7-331918.3) | 1366.5 (1079.7-1792) | 0 (0-0) |
| Democratic Republic of the Congo | 189120.3 (147626.9-246282.9) | 1320.2 (1030.5-1719.2) | 474052.5 (371341.7-617592.3) | 1313.7 (1029-1711.4) | 0 (0-0) |
| San Marino | 170.6 (135.4-215.9) | 1816.1 (1441.2-2299.1) | 162.4 (130.1-206.4) | 1812.3 (1452.2-2303.4) | 0 (0-0) |
| Morocco | 154166.6 (120419.3-198666.5) | 1482.8 (1158.2-1910.9) | 216213 (169681.5-279136.2) | 1472.7 (1155.8-1901.3) | -0.02 (-0.03--0.01) |
| Malta | 2459.8 (1971.2-3103.4) | 1782.6 (1428.4-2248.9) | 2331.3 (1853.2-2955.2) | 1741.7 (1384.5-2207.8) | -0.11 (-0.14--0.08) |
| Greenland | 438.3 (344.2-556.3) | 1655.9 (1300.3-2101.6) | 348.3 (278.8-438.7) | 1707.6 (1367-2150.5) | 0 (0-0) |
| Nicaragua | 18821.4 (14543.9-24581.9) | 1275.1 (985.3-1665.4) | 35933.9 (28179.8-46801) | 1262.6 (990.1-1644.4) | -0.03 (-0.03--0.03) |
| Panama | 12699 (9853.8-16565) | 1255.4 (974.1-1637.6) | 20675.9 (16185.1-26958.7) | 1252.7 (980.6-1633.4) | 0 (-0.01-0) |
| Algeria | 148450.3 (115051.4-193860.2) | 1469.2 (1138.6-1918.6) | 250200.3 (195294.6-322698) | 1469.4 (1147-1895.2) | 0.01 (0.01-0.02) |
| Comoros | 1753.3 (1380.4-2286) | 1015 (799.1-1323.4) | 3108.4 (2454-4034.5) | 1003.4 (792.2-1302.3) | -0.04 (-0.04--0.03) |
| Djibouti | 1744.9 (1374-2285.4) | 995.2 (783.7-1303.5) | 5317.3 (4219.6-6918.4) | 983.3 (780.3-1279.4) | 0 (0-0) |
| Nepal | 113774.2 (88799.6-146168.9) | 1557.5 (1215.6-2000.9) | 210525 (164477.7-272342.1) | 1570.2 (1226.7-2031.2) | 0 (0-0) |
| Seychelles | 529.4 (414.2-680.8) | 1695.8 (1326.7-2180.7) | 629.5 (501.8-810.4) | 1639.9 (1307.3-2111.3) | 0 (0-0) |
| Somalia | 28851.5 (22852-37731.9) | 995.1 (788.2-1301.4) | 82839.6 (65301.2-107682) | 999.2 (787.6-1298.8) | 0 (0-0) |
| Equatorial Guinea | 2026.5 (1590.4-2624.3) | 1342 (1053.2-1737.9) | 8934.5 (6992.4-11667.2) | 1284.4 (1005.2-1677.3) | -0.16 (-0.17--0.15) |
| Colombia | 180522.8 (140342.1-234783.9) | 1284.5 (998.6-1670.6) | 256236.4 (198698.4-332217) | 1275.2 (988.8-1653.3) | -0.03 (-0.03--0.02) |
| Tokelau | 8.9 (7.1-11.6) | 1536.2 (1218.5-2002) | 7.5 (6-9.7) | 1523.5 (1207-1965.7) | 0 (0-0) |
| Mali | 43886.7 (34407.8-56322.6) | 1469.9 (1152.4-1886.4) | 130811.4 (102183.9-169167.5) | 1468.6 (1147.2-1899.2) | 0 (0-0) |
| Grenada | 422.5 (328.7-547.9) | 1267.1 (985.8-1643.2) | 505.4 (395.2-653.7) | 1250.4 (977.7-1617.2) | -0.03 (-0.04--0.03) |
| Czechia | 52569 (42212-68467) | 1416.7 (1137.6-1845.2) | 41444.4 (33014.8-53244.7) | 1403.4 (1118-1803) | -0.04 (-0.05--0.03) |
| Mauritania | 11230.3 (8805.5-14414) | 1460.7 (1145.3-1874.8) | 25002.3 (19573.2-32088.5) | 1464.7 (1146.6-1879.8) | 0 (0-0) |
| Tuvalu | 55.7 (44.1-71.6) | 1543.4 (1222-1982.8) | 75 (59.3-96.5) | 1510.2 (1193.1-1942.3) | 0 (0-0) |
| Benin | 25133.8 (19712.6-32419.2) | 1476 (1157.7-1903.9) | 76662.3 (60042.7-98712.1) | 1462.3 (1145.3-1882.9) | 0 (0-0) |
| Hungary | 52336.8 (41799.5-68118.9) | 1416.1 (1131-1843.1) | 38714 (30890.4-50031.7) | 1407 (1122.7-1818.3) | -0.03 (-0.04--0.02) |
| United Republic of Tanzania | 97557.2 (76518.6-127537.6) | 1006.9 (789.8-1316.3) | 240684.8 (187379-312498.7) | 1031.5 (803-1339.3) | 0 (0-0) |
| Democratic People's Republic of Korea | 111094.9 (87269.3-142850.9) | 1331.9 (1046.3-1712.7) | 129574.1 (102803.4-166997.6) | 1288.2 (1022.1-1660.3) | -0.12 (-0.13--0.11) |
| United Arab Emirates | 12277.5 (9606.7-15758.6) | 1284.4 (1005-1648.6) | 48309.4 (36466.3-64690.7) | 1202.5 (907.7-1610.3) | -0.26 (-0.29--0.22) |
| Niger | 40830.4 (31901-52500.5) | 1467 (1146.1-1886.2) | 130844.7 (101888.9-170650.4) | 1467.3 (1142.6-1913.7) | 0 (0-0) |
| Afghanistan | 47543.2 (36395.7-63005.7) | 1509.5 (1155.6-2000.5) | 179471.8 (136534.1-233663.8) | 1468.6 (1117.2-1912) | 0 (0-0) |
| Viet Nam | 488905.7 (382344.3-628411.7) | 1714.4 (1340.7-2203.6) | 638791.6 (510001-819623) | 1663.9 (1328.5-2135) | -0.09 (-0.09--0.08) |
| Palestine | 11282.9 (8616.9-14715.1) | 1470.1 (1122.7-1917.3) | 32111.6 (25088.2-41218.3) | 1470.6 (1148.9-1887.6) | 0.02 (0.01-0.03) |
| Uganda | 65364.3 (51380.6-85360.9) | 1019 (801-1330.8) | 174888.4 (137693.9-228403.7) | 1016.9 (800.6-1328) | 0 (0-0) |
| Monaco | 161.5 (129.3-203.2) | 1764.8 (1413.1-2220.9) | 167.2 (133.8-210.9) | 1793.2 (1435.3-2262.1) | 0 (0-0) |
| Burkina Faso | 47189.8 (36865.2-60705.8) | 1480.2 (1156.4-1904.2) | 127429.2 (99808-163474.2) | 1472.4 (1153.2-1888.9) | 0 (0-0) |
| United Kingdom | 384152.4 (317530.7-464757) | 1838.1 (1519.3-2223.7) | 401347.4 (333978.2-487278.5) | 1845.1 (1535.4-2240.2) | 0.06 (0.04-0.07) |
| Nigeria | 528997 (432605.6-644864.9) | 1549.3 (1267-1888.7) | 1417513 (1150446-1733000.2) | 1576.1 (1279.1-1926.9) | 0 (0-0) |
| Barbados | 1392.7 (1093.1-1819.1) | 1276 (1001.5-1666.6) | 1261.9 (993.3-1644.6) | 1277.5 (1005.5-1664.9) | 0.01 (0-0.01) |
| Cameroon | 55842 (43704-71845.3) | 1468.3 (1149.1-1889) | 187653.4 (147201.6-241226.1) | 1455.5 (1141.7-1871) | 0 (0-0) |
| Iraq | 104513.4 (81153.7-136486.1) | 1454.6 (1129.5-1899.6) | 254079.9 (199944.8-326734.8) | 1457.2 (1146.7-1873.9) | 0.01 (0-0.02) |
| Sudan | 113822 (88594.3-148308.5) | 1493.6 (1162.6-1946.2) | 273447.6 (214221.5-351280) | 1479.2 (1158.9-1900.3) | 0 (0-0) |
| Paraguay | 19763.7 (15291.1-25544.1) | 1261 (975.6-1629.8) | 38660.7 (30199.5-50594.2) | 1263.6 (987.1-1653.6) | -0.01 (-0.01-0) |
| Guinea | 30241.5 (23747.7-39033.4) | 1472 (1155.9-1900) | 76119.8 (59633.8-97686.7) | 1473.2 (1154.1-1890.6) | 0 (0-0) |
| Angola | 51445.4 (40301.4-67156.6) | 1314.8 (1030-1716.4) | 161809.7 (126717.1-210895.6) | 1330 (1041.6-1733.5) | 0 (0-0) |
| Belize | 926.7 (716.8-1210.9) | 1266.6 (979.6-1655) | 2411.1 (1882.4-3101.7) | 1277.1 (997.1-1642.9) | 0.03 (0.02-0.03) |
| Jordan | 22149.6 (16832.8-28979.3) | 1441.1 (1095.2-1885.5) | 76704.2 (60417.9-98268.2) | 1428.9 (1125.5-1830.6) | -0.04 (-0.06--0.02) |
| Nauru | 61.9 (48.8-79.8) | 1530.1 (1207.9-1974.1) | 71.2 (56.2-91.8) | 1529.1 (1208.3-1972.1) | 0 (0-0) |
| Niue | 12.3 (9.7-16) | 1522.8 (1207.6-1985.8) | 8.7 (6.9-11.2) | 1524.4 (1209.9-1970) | 0 (0-0) |
| Gabon | 5055.1 (3947.4-6596.7) | 1314.3 (1026.3-1715.1) | 9965.1 (7815.7-13000.9) | 1329.5 (1042.7-1734.5) | 0 (0-0) |
| Guinea-Bissau | 5454.2 (4266.1-7006.2) | 1470.8 (1150.4-1889.3) | 12328 (9677.2-15898.1) | 1461 (1146.9-1884.1) | 0 (0-0) |
| South Sudan | 23090.1 (18177.5-30267.1) | 1000.2 (787.4-1311.1) | 36509.9 (28767.4-47830.6) | 1014.4 (799.3-1329) | 0 (0-0) |
| Mozambique | 48768.3 (38577.9-63669.5) | 1028.2 (813.4-1342.4) | 123119 (97002-160908.1) | 1024 (806.8-1338.3) | 0 (0-0) |
| Saint Lucia | 719.3 (556.7-935.8) | 1278.6 (989.6-1663.5) | 836.3 (658-1084.8) | 1265.2 (995.5-1641.3) | -0.04 (-0.05--0.04) |
| Eswatini | 4065.7 (3168.4-5258.4) | 1349.1 (1051.3-1744.8) | 6711.3 (5275.2-8710.9) | 1318.2 (1036.1-1710.9) | 0 (0-0) |
| Liberia | 13461.2 (10564.1-17361.9) | 1459.1 (1145.1-1881.9) | 32640 (25642.3-41787.8) | 1453.8 (1142.1-1861.2) | 0 (0-0) |
| Taiwan (Province of China) | 120706.9 (95637.5-153518.2) | 1308.3 (1036.6-1663.9) | 102219.4 (81606.3-132107) | 1354.8 (1081.6-1751) | 0.2 (0.15-0.25) |
| Bosnia and Herzegovina | 26602.7 (21282.2-34161.6) | 1400.8 (1120.6-1798.8) | 14152.5 (11279.4-18257.3) | 1406.4 (1120.9-1814.3) | -0.02 (-0.04-0) |
| Rwanda | 27697.7 (21907.2-36342.8) | 1010.7 (799.4-1326.1) | 57187.9 (45402.8-74719.8) | 1008.4 (800.6-1317.5) | 0 (0-0) |
| Republic of Korea | 275868.5 (217325.1-359981.4) | 1310.7 (1032.5-1710.3) | 198502.5 (158025-254815.8) | 1240.5 (987.6-1592.4) | -0.24 (-0.27--0.21) |
| Bangladesh | 635118.4 (493447.3-821264.5) | 1504.7 (1169-1945.7) | 1042190.6 (827356.2-1347768.3) | 1514.5 (1202.3-1958.6) | 0 (0-0) |
| Bahrain | 3519.3 (2738.3-4559.5) | 1372.9 (1068.2-1778.7) | 9256.8 (7233.4-11989.9) | 1315.7 (1028.1-1704.2) | -0.22 (-0.26--0.19) |
| Singapore | 15140 (12059.1-19013.4) | 1003.1 (799-1259.8) | 20848.1 (16616.6-27285.4) | 1083.7 (863.8-1418.4) | 0.31 (0.21-0.41) |
| Armenia | 20546.6 (16353.5-26207.8) | 1429.6 (1137.9-1823.5) | 15238.1 (12227.7-19418.7) | 1417.4 (1137.4-1806.3) | -0.05 (-0.08--0.02) |
| Belarus | 52246.9 (41853.2-67611) | 1324.6 (1061.1-1714.1) | 38430.3 (30774.8-50190.5) | 1310 (1049-1710.8) | -0.04 (-0.05--0.03) |
| Estonia | 7505 (6009.9-9756.7) | 1321.3 (1058.1-1717.8) | 5150.5 (4105.4-6727.9) | 1302.4 (1038.1-1701.3) | -0.07 (-0.08--0.06) |
| France | 396390 (318227.4-507765.6) | 1801.7 (1446.4-2308) | 357904.7 (286660.1-458464.5) | 1801.5 (1442.9-2307.7) | 0.02 (0.01-0.02) |
| Bulgaria | 42177.4 (33690.3-54529.3) | 1416.8 (1131.7-1831.7) | 26610.4 (21190.9-34200.2) | 1400.8 (1115.5-1800.4) | -0.05 (-0.06--0.05) |
| Croatia | 25559 (20383.2-33043.3) | 1408.6 (1123.3-1821.1) | 17604.7 (14013.4-22689.6) | 1410.4 (1122.7-1817.8) | -0.03 (-0.1-0.03) |
| Antigua and Barbuda | 330.5 (258.1-427.7) | 1281.7 (1000.8-1658.6) | 438.8 (344.9-570.9) | 1275.5 (1002.7-1659.7) | -0.03 (-0.06--0.01) |
| Guam | 947.5 (745.9-1225.4) | 1493.9 (1175.9-1931.9) | 838.1 (664.2-1076.9) | 1511.8 (1198.1-1942.6) | 0 (0-0) |
| Zimbabwe | 52920.2 (41338.3-68393.5) | 1334.8 (1042.7-1725.1) | 84216 (66274.4-109766.6) | 1328.8 (1045.7-1732) | 0 (0-0) |
| United States Virgin Islands | 513.7 (403-665.8) | 1296.8 (1017.4-1680.7) | 297.1 (234.5-386.8) | 1284.4 (1013.8-1672.5) | 0 (0-0) |
| Bahamas | 1505 (1172.8-1948.3) | 1275.2 (993.8-1650.9) | 1983 (1561.1-2584.1) | 1282.2 (1009.4-1670.8) | 0.02 (0.01-0.02) |
| Germany | 529215.4 (418619.3-663146) | 1781.4 (1409.1-2232.2) | 455697.1 (367886.7-570276.6) | 1801.3 (1454.2-2254.2) | 0.05 (-0.02-0.13) |
| Malaysia | 119378.3 (94976-154143.3) | 1608.5 (1279.7-2076.9) | 221935.4 (177308.7-285407.9) | 1596.4 (1275.4-2052.9) | -0.01 (-0.02-0) |
| Spain | 272195.9 (216141.9-351425) | 1835.5 (1457.5-2369.7) | 223696.6 (179561.9-286338.7) | 1801.7 (1446.2-2306.2) | -0.11 (-0.16--0.06) |
| Micronesia (Federated States of) | 616.3 (487.4-804.9) | 1538.1 (1216.5-2009) | 648.4 (510.7-840.8) | 1526.8 (1202.5-1980) | -0.04 (-0.04--0.03) |
| Greece | 68595.5 (54403.1-87955.3) | 1824.7 (1447.2-2339.7) | 50317.5 (40570.5-63656.1) | 1807.5 (1457.4-2286.6) | -0.05 (-0.08--0.02) |
| Saint Vincent and the Grenadines | 580.4 (448.1-757) | 1263.7 (975.6-1648) | 523 (412.6-678.7) | 1265.6 (998.5-1642.4) | 0.02 (0.01-0.02) |
| Suriname | 2047.3 (1584.1-2654.1) | 1257.2 (972.8-1629.8) | 2739.8 (2161.2-3563) | 1276.4 (1006.8-1659.9) | 0.05 (0.05-0.06) |
| Sweden | 54734.7 (45725.3-65463.2) | 1864.6 (1557.7-2230.1) | 59824.5 (49887.6-72171.8) | 1845.6 (1539-2226.5) | 0.01 (-0.02-0.04) |
| Syrian Arab Republic | 70449.9 (54336.6-91850.7) | 1468.5 (1132.6-1914.6) | 76720.7 (59365-100157.6) | 1508.4 (1167.2-1969.2) | 0.1 (0.06-0.13) |
| Guatemala | 37624.6 (29179.3-49111.9) | 1273.7 (987.8-1662.6) | 86887.4 (67414.5-113109.1) | 1276.5 (990.4-1661.7) | 0.01 (0.01-0.02) |
| Tunisia | 50804.8 (39595.1-65386.5) | 1477 (1151.1-1900.9) | 64380.2 (50239.1-82980.4) | 1479.4 (1154.5-1906.8) | 0.03 (0.02-0.03) |
| Honduras | 21921.9 (16977.6-28661.1) | 1269.8 (983.4-1660.1) | 56301.9 (43586.2-73367.3) | 1280.9 (991.6-1669.1) | 0.04 (0.04-0.05) |
| Mexico | 473329.8 (382481.9-581531.4) | 1327.3 (1072.5-1630.7) | 687131.7 (568489.1-847490.4) | 1333.9 (1103.6-1645.2) | 0.03 (0.02-0.03) |
| Switzerland | 44064.2 (35430-56427.5) | 1672.3 (1344.6-2141.5) | 46694.8 (37701.5-59556.5) | 1681.8 (1357.9-2145) | 0.06 (0.04-0.09) |
| Cabo Verde | 1923.8 (1501.8-2481.1) | 1471.2 (1148.5-1897.5) | 3571.7 (2845.9-4655) | 1425.1 (1135.5-1857.4) | 0 (0-0) |
| Zambia | 32613.8 (24867.8-42415.6) | 1074.9 (819.6-1397.9) | 86431.4 (66500-112963.8) | 1068 (821.7-1395.8) | 0 (0-0) |
| Chad | 30885 (24173.1-39680.2) | 1471.4 (1151.6-1890.4) | 92580.7 (72180.9-120199) | 1471.2 (1147-1910.1) | 0 (0-0) |
| Northern Mariana Islands | 354.5 (279.4-459) | 1513.8 (1193-1960) | 249.2 (199.2-321.6) | 1511.4 (1208-1950.5) | 0 (0-0) |
| Palau | 105.7 (83.5-136) | 1515.2 (1196.9-1949.7) | 86.1 (68.9-109.9) | 1461.9 (1169-1865.3) | 0 (0-0) |
| Sao Tome and Principe | 629.1 (491.4-815.4) | 1465.5 (1144.7-1899.6) | 1317 (1034.7-1693.5) | 1449.1 (1138.5-1863.3) | 0 (0-0) |
| Pakistan | 634640.5 (516378.9-762005.3) | 1555.3 (1265.5-1867.4) | 1543118.9 (1276999.4-1877449.2) | 1560.2 (1291.1-1898.2) | 0 (-0.01-0.01) |
| Egypt | 322941.8 (252907.7-419858) | 1473.1 (1153.6-1915.2) | 601237.6 (474887.6-763399.2) | 1424.3 (1125-1808.5) | -0.15 (-0.17--0.12) |
| North Macedonia | 11192.3 (8977.2-14438.3) | 1409.4 (1130.5-1818.2) | 10675.8 (8516.6-13775.5) | 1395.9 (1113.6-1801.3) | -0.04 (-0.05--0.04) |
| Senegal | 40573.2 (31702.4-52281.2) | 1470.3 (1148.9-1894.6) | 93669.7 (73447-120315.7) | 1452.7 (1139.1-1866) | 0 (0-0) |
| Iran (Islamic Republic of) | 343965 (281642.5-419808.8) | 1583.8 (1296.8-1933) | 550060.3 (454229.5-669812.8) | 1585 (1308.9-1930.1) | 0.05 (0.01-0.08) |
| Eritrea | 13120.4 (10381.2-17219.6) | 1012.5 (801.1-1328.8) | 27896.2 (22077.7-36340) | 996.3 (788.5-1297.9) | 0 (0-0) |
| Kyrgyzstan | 25875.3 (20398.5-33184.5) | 1434.6 (1131-1839.9) | 38779.2 (30869.3-49604.4) | 1424.9 (1134.3-1822.7) | -0.02 (-0.03--0.01) |
| Ethiopia | 174266.3 (141004.4-212739.2) | 953.9 (771.8-1164.4) | 444590.8 (362348.4-543989.6) | 959.1 (781.7-1173.5) | 0.04 (0.03-0.06) |
| Republic of Moldova | 23246 (18668.3-30203.6) | 1333.8 (1071.2-1733) | 16209.9 (12970.4-21200.7) | 1307 (1045.8-1709.4) | -0.08 (-0.08--0.07) |
