# Supplementary material for: Global, regional, and national burden and trends of migraine among youths and young adults aged 15–39 years from 1990 to 2021: findings from the global burden of disease study 2021
Source: J Headache Pain. 2024 Aug 12;25(1):131. doi: 10.1186/s10194-024-01832-0 (PMC11318134; doi:10.1186/s10194-024-01832-0)
Supplement: Supplementary file 28 — Supplementary Material 28: Table S6: The global disease burden of migraine DALYs for both sexes in 204 countries and territories. [file 10194_2024_1832_MOESM28_ESM.docx]

| **TableS6: The global disease burden of migraine DALYs for both sexes in 204 countries and territories.** | | | | | |
| --- | --- | --- | --- | --- | --- |
| **Location** | **1990** | | **2019** | | **EAPC_95%CI** |
|  | **Number** | **ASR** | **Number** | **ASR** |  |
| Maldives | 656.1 (51.2-1555) | 807.4 (63.1-1913.4) | 2068.8 (185.7-4718.4) | 795.2 (71.4-1813.6) | -0.06 (-0.08--0.04) |
| Myanmar | 140797.6 (11328.6-330360.5) | 820.1 (66-1924.3) | 186928.8 (14819.3-433668.6) | 831.5 (65.9-1929) | 0.06 (0.05-0.07) |
| Papua New Guinea | 11733.6 (1124.5-27523.3) | 708.9 (67.9-1662.8) | 30826.2 (2929.7-71207.1) | 720.3 (68.5-1663.9) | 0.04 (0.04-0.05) |
| Philippines | 219352 (18332.1-508227.6) | 846.3 (70.7-1960.8) | 402989.4 (33948.2-933828.1) | 852.9 (71.8-1976.3) | 0.03 (0.02-0.03) |
| Sri Lanka | 60869 (5201.9-144207) | 823.2 (70.3-1950.2) | 67172.5 (5576.4-158233.7) | 832.9 (69.1-1962.1) | 0.06 (0.05-0.07) |
| Samoa | 471.8 (43.5-1114.9) | 704.5 (64.9-1664.9) | 576.5 (53.6-1347.5) | 717.5 (66.7-1677.1) | 0.05 (0.04-0.06) |
| Romania | 59710.2 (9188.6-142833.1) | 687.3 (105.8-1644.2) | 37436.4 (5951.2-87938.9) | 694.6 (110.4-1631.7) | 0.09 (0.07-0.1) |
| Mongolia | 5948.7 (737.5-14499.7) | 673.2 (83.5-1640.8) | 8732.8 (1145.6-20650.1) | 691.9 (90.8-1636.1) | 0.09 (0.07-0.1) |
| Serbia | 24802 (3798.1-58868.9) | 690.6 (105.8-1639.3) | 20463.2 (3181.9-48014.3) | 690.3 (107.3-1619.8) | 0.03 (0.02-0.04) |
| Montenegro | 1722.5 (269-4073.7) | 686.2 (107.2-1622.8) | 1422 (225.4-3353.1) | 691.2 (109.6-1629.9) | 0.04 (0.04-0.05) |
| Belgium | 39928.5 (3433.9-89935) | 1073.2 (92.3-2417.2) | 40189.3 (3368.7-91035.6) | 1147.4 (96.2-2599.1) | 0.23 (0.17-0.3) |
| Solomon Islands | 911.8 (83.4-2130.1) | 710.9 (65-1660.7) | 1971.7 (186-4602.2) | 720.9 (68-1682.6) | 0.07 (0.06-0.08) |
| Central African Republic | 6459.2 (826.3-14729.8) | 620.3 (79.4-1414.5) | 13681.4 (1805.2-31273.1) | 626.7 (82.7-1432.4) | 0.05 (0.04-0.06) |
| Andorra | 233 (23.2-526.7) | 931.7 (92.8-2106) | 241.1 (25.8-540.5) | 945.4 (101.2-2119.8) | 0.05 (0.01-0.08) |
| Cyprus | 2896.8 (285.7-6581.3) | 941.9 (92.9-2139.9) | 4814.7 (510.7-10966.3) | 959.6 (101.8-2185.6) | 0.08 (0.07-0.09) |
| Cuba | 35694.8 (3654.6-81978.1) | 731.5 (74.9-1680) | 26136.7 (2813.3-59769.5) | 728.9 (78.5-1666.9) | 0 (-0.01-0.01) |
| Argentina | 72781.8 (10613.5-168827.1) | 595.8 (86.9-1382.1) | 108674.4 (15454.6-249241.5) | 620.2 (88.2-1422.4) | 0.19 (0.15-0.22) |
| Yemen | 37746.7 (5467.1-86555.7) | 821.1 (118.9-1882.9) | 113059.8 (17097-254927.6) | 821.7 (124.3-1852.7) | 0.04 (0.02-0.06) |
| Russian Federation | 448284.8 (93973.7-989020.6) | 770.6 (161.5-1700.2) | 363096.9 (77380.6-796428.5) | 781.3 (166.5-1713.7) | 0.12 (0.09-0.15) |
| Tonga | 264.1 (24.2-618.9) | 715.9 (65.6-1677.3) | 282.2 (26.9-664) | 725.5 (69.2-1707.1) | 0.06 (0.05-0.07) |
| Thailand | 256448.2 (18154.9-588873.9) | 988.9 (70-2270.8) | 194849 (15046.5-463790.2) | 919.1 (71-2187.7) | -0.27 (-0.35--0.18) |
| Dominica | 212.3 (21.4-487.5) | 726.8 (73.1-1669.1) | 188.3 (19.1-425.9) | 727.1 (73.9-1644.5) | 0.02 (0.01-0.03) |
| Slovakia | 14159.6 (2221.6-33413.5) | 691.7 (108.5-1632.3) | 12023.3 (1922.9-28131.2) | 702.6 (112.4-1643.8) | 0.09 (0.07-0.1) |
| Turkmenistan | 10411 (1355.7-24756) | 678.2 (88.3-1612.7) | 14034.3 (1848.1-33603.1) | 674.8 (88.9-1615.6) | -0.04 (-0.06--0.03) |
| Botswana | 3254 (409.9-7402.8) | 632.1 (79.6-1438.1) | 6782.5 (897.1-15247.8) | 636.6 (84.2-1431.2) | 0 (0-0) |
| Denmark | 15625.3 (1720.4-35257.4) | 819 (90.2-1847.9) | 15382 (1682.9-34864) | 843.5 (92.3-1911.8) | 0.09 (0.05-0.13) |
| Slovenia | 5328.4 (824.4-12698.4) | 695.3 (107.6-1657) | 3974.6 (633.2-9213.3) | 698 (111.2-1618.1) | 0.03 (0.02-0.04) |
| Kenya | 42528.2 (7221.3-99581.7) | 485.6 (82.5-1137.1) | 106498.5 (18090.4-246961.3) | 491.9 (83.6-1140.6) | 0.08 (0.06-0.09) |
| Tajikistan | 14342.4 (1888.8-34578.6) | 678.2 (89.3-1635.2) | 28563.5 (3714.4-68065.8) | 684.8 (89.1-1631.8) | 0.02 (0-0.04) |
| Brunei Darussalam | 689.9 (98.4-1617.9) | 559.6 (79.8-1312.3) | 1152 (176.5-2705.1) | 564.6 (86.5-1325.8) | -0.01 (-0.04-0.02) |
| Finland | 17221.1 (1763.5-38448.8) | 948.6 (97.1-2117.9) | 15697.8 (1566.4-35300.3) | 942.2 (94-2118.9) | -0.02 (-0.03--0.01) |
| Japan | 257800.5 (39899.1-602448.8) | 575.3 (89-1344.3) | 191694.1 (30166-439496.8) | 591.5 (93.1-1356) | 0.14 (0.11-0.16) |
| Cambodia | 31640.1 (2577.9-74730.1) | 821.8 (67-1940.9) | 60175.1 (4990.2-141085.1) | 830.8 (68.9-1947.8) | 0.05 (0.03-0.07) |
| Uzbekistan | 58109.9 (7343.1-140527.2) | 677 (85.5-1637.2) | 94775.4 (12693.1-223396.7) | 689.9 (92.4-1626.2) | 0.05 (0.04-0.07) |
| Kuwait | 6825.3 (995.8-15461.7) | 807.6 (117.8-1829.5) | 18122.3 (2726.6-40460.7) | 853.7 (128.4-1906.1) | 0.2 (0.15-0.25) |
| Madagascar | 20804.1 (3640.5-48998.8) | 459.3 (80.4-1081.7) | 54355 (9361.8-129406.5) | 464.1 (79.9-1105) | 0.05 (0.04-0.05) |
| Austria | 26827 (2777.1-61133.7) | 893.6 (92.5-2036.4) | 25258.2 (2808.3-57417.2) | 894.9 (99.5-2034.3) | 0.06 (0.03-0.09) |
| Vanuatu | 422.2 (37-980.3) | 720.8 (63.1-1673.4) | 901.2 (84.5-2098.9) | 723.1 (67.8-1684) | 0.02 (0.02-0.03) |
| Lesotho | 3466.4 (441.4-7944) | 642.4 (81.8-1472.3) | 5175.6 (689.4-11729) | 622.2 (82.9-1410) | -0.09 (-0.11--0.07) |
| Guyana | 2489.9 (253.2-5676.8) | 731.6 (74.4-1667.9) | 2260.1 (234.8-5172.8) | 727.3 (75.6-1664.7) | 0.01 (0-0.02) |
| Indonesia | 668357.7 (56026.6-1542714.4) | 856.4 (71.8-1976.7) | 985231.6 (82998.8-2268784.2) | 865.2 (72.9-1992.4) | 0.04 (0.03-0.05) |
| Chile | 34898.1 (4854.7-80998.6) | 609.3 (84.8-1414.2) | 44356.3 (6507.5-101887.8) | 626.9 (92-1440) | 0.12 (0.1-0.14) |
| Lao People's Democratic Republic | 12686.6 (1073.7-29803.9) | 821.1 (69.5-1929) | 26601.9 (2270-61957.1) | 829.3 (70.8-1931.4) | 0.03 (0.01-0.04) |
| Sierra Leone | 11896.3 (1371-27449.1) | 744.7 (85.8-1718.3) | 27660.4 (2975.9-64899.4) | 741.6 (79.8-1740.1) | -0.03 (-0.04--0.02) |
| Ukraine | 137398.8 (27403.7-306387.9) | 723.4 (144.3-1613) | 100942 (21739.8-221460.6) | 732.3 (157.7-1606.5) | 0.07 (0.04-0.1) |
| Bolivia (Plurinational State of) | 13692.4 (1925.6-32006.7) | 555.2 (78.1-1297.9) | 27084.4 (3641.1-62504.1) | 551.2 (74.1-1272) | -0.02 (-0.03--0.01) |
| Uruguay | 7073.6 (997.5-16296.7) | 622.8 (87.8-1434.8) | 7446.5 (1064.8-17245) | 622.5 (89-1441.6) | 0.02 (0.01-0.02) |
| Haiti | 17872.2 (1780-41034.3) | 734.2 (73.1-1685.7) | 40087.1 (4105.8-90420.3) | 730.3 (74.8-1647.2) | -0.01 (-0.02-0) |
| Ecuador | 24971.4 (3166.8-57059.9) | 605.2 (76.7-1382.8) | 45638.7 (5576.2-105273.6) | 624.8 (76.3-1441.2) | 0.17 (0.14-0.2) |
| Jamaica | 7292.9 (726.2-16582.8) | 742.1 (73.9-1687.4) | 8769.5 (891.4-19984.8) | 734.9 (74.7-1674.8) | -0.02 (-0.03--0.02) |
| Venezuela (Bolivarian Republic of) | 56077.8 (6082.7-127611.2) | 700.2 (75.9-1593.3) | 68999.8 (7519.5-158612.7) | 737 (80.3-1694.1) | 0.16 (0.13-0.18) |
| American Samoa | 146.4 (13.7-344.1) | 723.4 (67.8-1700.8) | 125.4 (11.7-294.6) | 717.6 (67-1685.9) | -0.03 (-0.04--0.02) |
| Iceland | 980.3 (96.2-2214.5) | 943.7 (92.6-2131.7) | 1127.4 (112.3-2590.5) | 942.1 (93.8-2164.7) | -0.02 (-0.03--0.01) |
| Puerto Rico | 10504 (1096.9-24057.8) | 742.6 (77.5-1700.7) | 7648.4 (801-17278.2) | 739.5 (77.4-1670.6) | -0.01 (-0.01-0) |
| Ireland | 12841.1 (1248.7-29061) | 936.1 (91-2118.6) | 14818.7 (1462.2-33577) | 947.1 (93.4-2145.9) | 0.07 (0.06-0.09) |
| Latvia | 6581.7 (1305.3-14994.3) | 689.9 (136.8-1571.8) | 3754.2 (765.1-8375.7) | 697 (142-1555.1) | 0.04 (0.01-0.06) |
| Albania | 9705.5 (1470.7-23194) | 683.3 (103.5-1632.8) | 6470.5 (1009.3-15358.2) | 682.5 (106.5-1619.9) | -0.04 (-0.06--0.02) |
| Lithuania | 8960.9 (1616.8-19949.2) | 643.1 (116-1431.8) | 5198.1 (933.3-11525.2) | 645.6 (115.9-1431.5) | -0.09 (-0.14--0.05) |
| Canada | 100446.4 (10075.9-227266.1) | 903.6 (90.6-2044.4) | 105154 (10353.9-233367.3) | 886.5 (87.3-1967.4) | -0.08 (-0.1--0.07) |
| Netherlands | 51817.6 (5028.1-117724.4) | 859.4 (83.4-1952.4) | 48310 (4524.5-110215) | 914.9 (85.7-2087.2) | 0.35 (0.26-0.45) |
| Poland | 105309.2 (16693.2-243059.7) | 729 (115.6-1682.7) | 89721 (14525.4-206154.2) | 741.6 (120.1-1704) | 0.1 (0.07-0.13) |
| Australia | 47287.9 (5891.5-110459.8) | 698.2 (87-1631) | 61197.4 (7755.3-139102.7) | 705.9 (89.5-1604.5) | 0.02 (0.01-0.03) |
| Italy | 219134.3 (19295.7-502169.4) | 1026.5 (90.4-2352.3) | 163385.2 (15035.9-374104.4) | 1034.4 (95.2-2368.5) | 0.18 (0.04-0.32) |
| Brazil | 587552.5 (45913.7-1344344.1) | 936.4 (73.2-2142.5) | 800868.5 (63202.6-1822980.4) | 939.4 (74.1-2138.4) | 0.08 (0.04-0.13) |
| Lebanon | 9645.8 (1447.8-21821.5) | 836.7 (125.6-1893) | 19403.2 (2974.4-42230.7) | 836.2 (128.2-1820) | 0 (-0.01-0.01) |
| Fiji | 2328.3 (228.4-5405.9) | 722.1 (70.8-1676.5) | 2588 (243.7-5949.6) | 725.5 (68.3-1667.8) | 0.04 (0.03-0.05) |
| Israel | 18091.1 (1816.7-40699.4) | 946.7 (95.1-2129.8) | 31371.6 (3103.8-71101.3) | 944.1 (93.4-2139.6) | 0.01 (0-0.01) |
| New Zealand | 10014.4 (1288.2-23109.3) | 724.9 (93.2-1672.8) | 13154.6 (1716.4-30038.5) | 730.2 (95.3-1667.5) | 0.01 (0-0.02) |
| Congo | 5898.1 (781.6-13393.8) | 622.6 (82.5-1413.8) | 14026.2 (1827.2-32142.9) | 633.1 (82.5-1450.7) | 0.08 (0.07-0.09) |
| Saint Kitts and Nevis | 126.6 (12.9-291.1) | 732.7 (74.8-1684.3) | 166.5 (17.7-375.3) | 732.6 (77.8-1651.2) | 0 (0-0.01) |
| Bermuda | 190.1 (20.1-431.2) | 739.1 (78.1-1676.9) | 129.8 (14.1-294.8) | 741.4 (80.3-1683.9) | 0.02 (0.01-0.02) |
| Togo | 10138.4 (1105.8-23801.6) | 739.6 (80.7-1736.2) | 25080.2 (2771.9-58125.6) | 745.4 (82.4-1727.6) | 0.04 (0.04-0.05) |
| Dominican Republic | 22821.4 (2291.5-52813.1) | 742.9 (74.6-1719.2) | 33213.7 (3389.9-76347.3) | 730.4 (74.5-1679) | -0.06 (-0.06--0.05) |
| Peru | 39841.4 (6528.3-94460.2) | 449.1 (73.6-1064.8) | 73558.7 (11293.8-172698.1) | 495 (76-1162.1) | 0.36 (0.26-0.46) |
| Kiribati | 219.7 (20.7-511.8) | 719.7 (67.9-1676.5) | 362.1 (34.5-858.3) | 728.2 (69.3-1726.2) | 0.02 (0.01-0.03) |
| United States of America | 943895.8 (97116.1-2123720.8) | 923.9 (95.1-2078.6) | 995964.7 (103440.1-2254286.4) | 894.8 (92.9-2025.4) | -0.04 (-0.13-0.05) |
| Norway | 13929.3 (1352-31601) | 870.7 (84.5-1975.3) | 16964.3 (1554.5-38044.4) | 955.9 (87.6-2143.6) | 0.39 (0.31-0.47) |
| Costa Rica | 9257.3 (982.4-20868.9) | 720.6 (76.5-1624.5) | 13811.6 (1434.9-30676) | 725.7 (75.4-1611.7) | 0.03 (0.02-0.04) |
| Trinidad and Tobago | 3672.3 (387.4-8475.8) | 732.5 (77.3-1690.6) | 3627.6 (403-8118.2) | 728.8 (81-1630.9) | -0.01 (-0.02--0.01) |
| Azerbaijan | 21869.2 (2878.8-52186.5) | 688.1 (90.6-1642.1) | 29385.2 (4053-69711.2) | 693.7 (95.7-1645.7) | -0.01 (-0.02-0.01) |
| Marshall Islands | 122.8 (11.4-289.5) | 715.9 (66.7-1687.6) | 170.6 (16.6-398.7) | 719.4 (70-1680.9) | 0.04 (0.03-0.05) |
| El Salvador | 15030.2 (1559.7-34337.5) | 719.4 (74.7-1643.5) | 18918.5 (2021.1-42285.8) | 729.9 (78-1631.4) | 0.06 (0.04-0.07) |
| Portugal | 35626.1 (3460.8-79728.9) | 941 (91.4-2105.8) | 27996.4 (2773.8-62498.3) | 948.5 (94-2117.4) | 0.06 (0.05-0.08) |
| Malawi | 17065.2 (2977.1-40557.5) | 456.7 (79.7-1085.4) | 37914.8 (6505.5-90935.3) | 463.3 (79.5-1111.2) | 0.1 (0.08-0.11) |
| Namibia | 3516.7 (444.8-8162.8) | 628.9 (79.5-1459.7) | 6621.7 (886.4-15072.9) | 633.6 (84.8-1442.3) | 0.05 (0.03-0.06) |
| China | 3054389.9 (371197.3-6983305.2) | 557.2 (67.7-1274) | 2850689.3 (332940-6332746) | 617.8 (72.2-1372.4) | 0.33 (0.29-0.36) |
| Gambia | 2791.4 (303.9-6576.9) | 740.4 (80.6-1744.4) | 7387.2 (814.8-17236.7) | 738.6 (81.5-1723.4) | 0.01 (0.01-0.01) |
| Luxembourg | 1377.7 (168.5-3121.2) | 933.5 (114.1-2115) | 2062.9 (255.3-4654.3) | 935.1 (115.7-2109.8) | -0.02 (-0.03-0) |
| Libya | 13611.7 (2002.4-31841.4) | 810.1 (119.2-1895.1) | 24998.1 (3747.2-56371.5) | 833.1 (124.9-1878.7) | 0.08 (0.07-0.09) |
| South Africa | 103459 (14352.8-231619.5) | 657.5 (91.2-1471.9) | 159910.3 (22588.7-354513) | 659.5 (93.2-1462.1) | 0 (-0.01-0.02) |
| Ghana | 42373.3 (4696.3-99730) | 738.2 (81.8-1737.4) | 106771.1 (11983.8-247444.7) | 746.6 (83.8-1730.2) | 0.04 (0.04-0.05) |
| Oman | 6395.4 (1017.2-14404.3) | 770.8 (122.6-1736.1) | 18099.8 (2914.1-39531.2) | 782.1 (125.9-1708.1) | 0.03 (-0.01-0.07) |
| Georgia | 14836.7 (1990.3-35313.8) | 696.9 (93.5-1658.7) | 7846.2 (1060-18632.6) | 691.5 (93.4-1642.1) | -0.03 (-0.04--0.01) |
| Qatar | 1776.2 (293.8-3925.2) | 751.2 (124.2-1660) | 12369.6 (2032.6-27359.3) | 748.5 (123-1655.6) | -0.17 (-0.25--0.1) |
| Timor-Leste | 2573.9 (215.4-5978.4) | 808.6 (67.7-1878.2) | 4659.6 (370.8-10946.7) | 815.6 (64.9-1916) | 0.03 (0.02-0.05) |
| Cook Islands | 55.6 (5.1-130.8) | 719.9 (66.4-1694.2) | 43.4 (4.1-100.9) | 736.2 (70-1712.3) | 0.07 (0.06-0.08) |
| Mauritius | 4097.5 (343.8-9588.2) | 824.2 (69.2-1928.8) | 3764 (316.8-8687.5) | 826.9 (69.6-1908.5) | 0.02 (0.01-0.02) |
| Kazakhstan | 46519 (6032.8-111179.5) | 685.2 (88.9-1637.6) | 48416.2 (6527-114968.8) | 694.7 (93.7-1649.6) | 0.05 (0.03-0.07) |
| Bhutan | 1912.2 (165.6-4432.1) | 709.6 (61.4-1644.8) | 2533.5 (231.2-5898.3) | 731 (66.7-1701.8) | 0.09 (0.08-0.1) |
| Burundi | 9654.1 (1692.1-22442.8) | 465.7 (81.6-1082.6) | 24497.2 (4281.1-57565.5) | 464.6 (81.2-1091.9) | -0.01 (-0.05-0.02) |
| India | 2522978.9 (240562.7-5763475.9) | 739.8 (70.5-1690) | 4561729.4 (438783.4-10306454.7) | 748.5 (72-1691.1) | 0.01 (-0.03-0.04) |
| Saudi Arabia | 51918.4 (8017.4-118352.4) | 781.3 (120.6-1781) | 147293.5 (23521.5-324193.9) | 795.2 (127-1750.3) | 0.04 (0.03-0.05) |
| Democratic Republic of the Congo | 88137.7 (11424-201608.9) | 615.3 (79.7-1407.4) | 224140.4 (27844-517143.7) | 621.1 (77.2-1433.1) | 0.04 (0.03-0.05) |
| San Marino | 89.5 (8.8-204.4) | 953.1 (93.3-2176.8) | 85.2 (8.7-191.3) | 950.4 (96.7-2134.3) | -0.01 (-0.05-0.03) |
| Morocco | 86333.7 (12979.5-196007.3) | 830.4 (124.8-1885.3) | 122684.3 (18671.5-271855.3) | 835.6 (127.2-1851.7) | 0.02 (0.02-0.03) |
| Malta | 1307.5 (130.1-2932.5) | 947.5 (94.3-2125.1) | 1271.4 (131.7-2906.4) | 949.8 (98.4-2171.3) | 0.01 (0-0.03) |
| Greenland | 225.9 (22.2-510.2) | 853.3 (84-1927.4) | 178 (18.1-399.5) | 872.6 (88.9-1958.5) | 0.03 (0.01-0.06) |
| Nicaragua | 10631.9 (1095.8-24003.3) | 720.3 (74.2-1626.2) | 20442.9 (2233.1-45245) | 718.3 (78.5-1589.7) | -0.01 (-0.01-0) |
| Panama | 7242.6 (793-16277.6) | 716 (78.4-1609.1) | 11783.4 (1268.6-26339.2) | 714 (76.9-1595.9) | 0 (0-0.01) |
| Algeria | 83329.4 (12676.9-190230) | 824.7 (125.5-1882.6) | 143837.6 (21461.8-315910.7) | 844.8 (126-1855.4) | 0.09 (0.08-0.09) |
| Comoros | 798.6 (135.9-1902.6) | 462.3 (78.7-1101.4) | 1454.9 (256.3-3411.9) | 469.7 (82.7-1101.4) | 0.06 (0.06-0.07) |
| Djibouti | 799.9 (141.7-1886.1) | 456.2 (80.8-1075.8) | 2532.9 (432-5911.9) | 468.4 (79.9-1093.3) | 0.11 (0.1-0.12) |
| Nepal | 56134.5 (5149.2-131837.6) | 768.4 (70.5-1804.7) | 104710.2 (9391.6-239425.4) | 781 (70-1785.7) | 0.06 (0.06-0.07) |
| Seychelles | 256.6 (20.6-607.3) | 822.1 (66-1945.3) | 314.8 (27.3-718.7) | 820 (71.2-1872.3) | -0.03 (-0.04--0.01) |
| Somalia | 13209.6 (2351.9-31175.2) | 455.6 (81.1-1075.3) | 37648.6 (6598.2-88895.9) | 454.1 (79.6-1072.2) | 0.05 (0.02-0.07) |
| Equatorial Guinea | 944.3 (121.5-2160.1) | 625.4 (80.5-1430.5) | 4248.3 (543.4-9677.2) | 610.7 (78.1-1391.2) | -0.08 (-0.09--0.07) |
| Colombia | 102904.6 (10744.1-236623.8) | 732.2 (76.4-1683.6) | 147796.3 (15218.2-341384.5) | 735.5 (75.7-1698.9) | 0.04 (0.03-0.05) |
| Tokelau | 4.2 (0.4-9.8) | 720.6 (68.3-1680.7) | 3.6 (0.3-8.3) | 723 (69.2-1673.7) | -0.01 (-0.02-0.01) |
| Mali | 22134.1 (2429-51862.6) | 741.3 (81.4-1737) | 65693.6 (7148.7-153938.2) | 737.5 (80.3-1728.3) | 0.01 (0-0.02) |
| Grenada | 243.6 (24.4-558.1) | 730.7 (73.2-1673.8) | 292.8 (30.2-674.5) | 724.3 (74.8-1668.9) | -0.02 (-0.03--0.02) |
| Czechia | 25583.6 (3983-60823.7) | 689.5 (107.3-1639.2) | 20611.3 (3260.9-48798.4) | 698 (110.4-1652.5) | 0.1 (0.08-0.11) |
| Mauritania | 5703.6 (629.3-13246.6) | 741.9 (81.9-1723) | 12707.4 (1431.4-29655.8) | 744.4 (83.9-1737.3) | 0.02 (0.01-0.02) |
| Tuvalu | 26.7 (2.5-61.7) | 738.6 (69.7-1709.5) | 35.6 (3.4-83.8) | 717.4 (68.5-1686.9) | -0.08 (-0.1--0.07) |
| Benin | 12661.9 (1426.2-29307.6) | 743.6 (83.8-1721.1) | 38896.7 (4330.4-91134.4) | 741.9 (82.6-1738.4) | -0.01 (-0.02--0.01) |
| Hungary | 25621.7 (3971.4-60365.5) | 693.2 (107.5-1633.3) | 19192.6 (2988.2-45203) | 697.5 (108.6-1642.8) | 0.08 (0.06-0.1) |
| United Republic of Tanzania | 43668.1 (7986.7-101288) | 450.7 (82.4-1045.4) | 111756.4 (18748-260921.1) | 479 (80.3-1118.2) | 0.34 (0.28-0.4) |
| Democratic People's Republic of Korea | 51039.9 (5576.3-115477.1) | 611.9 (66.9-1384.5) | 61044.7 (6503.4-137597.1) | 606.9 (64.7-1368) | -0.08 (-0.1--0.06) |
| United Arab Emirates | 7272.2 (1190.8-16217.6) | 760.8 (124.6-1696.6) | 30213 (5466.4-65831.6) | 752.1 (136.1-1638.7) | -0.06 (-0.1--0.02) |
| Niger | 20554.7 (2322.2-48193.9) | 738.5 (83.4-1731.5) | 65766.6 (7014.6-155037.9) | 737.5 (78.7-1738.6) | 0.01 (0-0.02) |
| Afghanistan | 25694 (3690.2-59332.5) | 815.8 (117.2-1883.8) | 98751.6 (14636.4-226622) | 808 (119.8-1854.4) | 0 (-0.03-0.02) |
| Viet Nam | 236201.4 (19770.4-554544.4) | 828.3 (69.3-1944.6) | 323690.6 (27238.5-746611.7) | 843.2 (71-1944.8) | 0.06 (0.04-0.07) |
| Palestine | 6285.3 (917.3-14544.4) | 818.9 (119.5-1895) | 17986.5 (2677.6-40537.8) | 823.7 (122.6-1856.4) | 0.03 (0.03-0.04) |
| Uganda | 29173 (5211.4-68745.3) | 454.8 (81.2-1071.7) | 79415.9 (13678.6-189903.7) | 461.8 (79.5-1104.2) | 0.06 (0.06-0.07) |
| Monaco | 88 (8.9-196.9) | 962.4 (97.8-2152.5) | 88.3 (9.3-197.7) | 947.1 (99.3-2120.6) | -0.03 (-0.04--0.03) |
| Burkina Faso | 23620.7 (2706.2-54983.9) | 740.9 (84.9-1724.7) | 64812.1 (7109.9-151719.9) | 748.9 (82.2-1753.1) | 0.04 (0.04-0.05) |
| United Kingdom | 194589.3 (20307.6-438825.5) | 931.1 (97.2-2099.7) | 204966.2 (21853.4-454897) | 942.3 (100.5-2091.3) | 0.02 (0.01-0.04) |
| Nigeria | 262451.3 (28908.9-594500.3) | 768.7 (84.7-1741.1) | 695387.5 (76983.1-1580414.5) | 773.2 (85.6-1757.2) | 0.03 (0.02-0.04) |
| Barbados | 806.6 (83.6-1870.5) | 739 (76.5-1713.7) | 728 (75.6-1656.6) | 737 (76.5-1677.1) | 0 (0-0.01) |
| Cameroon | 28068.4 (3130-65359.8) | 738 (82.3-1718.5) | 95505.7 (10493.5-222705.9) | 740.8 (81.4-1727.4) | 0.03 (0.02-0.03) |
| Iraq | 58180.1 (8606.8-131947.6) | 809.7 (119.8-1836.4) | 143102.3 (20819.9-321128.5) | 820.7 (119.4-1841.7) | 0.06 (0.05-0.07) |
| Sudan | 63372 (9183-144879.8) | 831.6 (120.5-1901.2) | 153786 (22519.1-351696.3) | 831.9 (121.8-1902.5) | 0.02 (0.01-0.03) |
| Paraguay | 14221.4 (1079.4-33121) | 907.4 (68.9-2113.3) | 27250.1 (2118.2-63487.3) | 890.7 (69.2-2075.1) | -0.06 (-0.07--0.04) |
| Guinea | 15345.8 (1729.9-35684.4) | 747 (84.2-1737) | 38653.6 (4293.5-89728.8) | 748.1 (83.1-1736.6) | 0.03 (0.02-0.03) |
| Angola | 24327.1 (3152.4-56138.2) | 621.8 (80.6-1434.8) | 76524.9 (9890.7-176981.5) | 629 (81.3-1454.7) | 0.07 (0.06-0.08) |
| Belize | 537.3 (53.9-1249.7) | 734.4 (73.7-1708) | 1390.1 (139.6-3169.1) | 736.3 (73.9-1678.6) | 0.02 (0.01-0.02) |
| Jordan | 12459.9 (1825.4-28812.3) | 810.7 (118.8-1874.6) | 43784.8 (6473.6-98925) | 815.6 (120.6-1842.8) | 0.02 (0-0.04) |
| Nauru | 29.3 (2.7-68.3) | 724 (67.2-1689.9) | 33.6 (3.2-78.1) | 721.7 (68-1677.3) | -0.01 (-0.01-0) |
| Niue | 5.8 (0.5-13.6) | 716.5 (66.4-1694.7) | 4.1 (0.4-9.6) | 721.7 (67.7-1698.8) | 0.03 (0.03-0.04) |
| Gabon | 2391.3 (305.9-5449.7) | 621.7 (79.5-1416.9) | 4754.1 (595.1-10851.1) | 634.3 (79.4-1447.7) | 0.07 (0.07-0.08) |
| Guinea-Bissau | 2738.1 (301.3-6349.2) | 738.4 (81.3-1712.1) | 6270.5 (704.8-14568.4) | 743.1 (83.5-1726.6) | 0.04 (0.03-0.05) |
| South Sudan | 10436 (1838.8-24819.1) | 452.1 (79.7-1075.1) | 16504.1 (2890.6-39278.7) | 458.6 (80.3-1091.3) | 0.08 (0.06-0.09) |
| Mozambique | 21981.3 (3703.2-51949.6) | 463.4 (78.1-1095.3) | 55028.7 (9487.1-128779.6) | 457.7 (78.9-1071.1) | -0.02 (-0.03--0.01) |
| Saint Lucia | 414.5 (42.7-962.7) | 736.8 (75.9-1711.1) | 480.2 (48.5-1079.4) | 726.5 (73.4-1633.2) | -0.04 (-0.05--0.03) |
| Eswatini | 1923.6 (242.4-4421.1) | 638.3 (80.4-1467) | 3197.6 (423.9-7427.3) | 628.1 (83.3-1458.8) | -0.05 (-0.07--0.02) |
| Liberia | 6669.3 (761.8-15577.9) | 722.9 (82.6-1688.5) | 16318.2 (1816.4-37856.9) | 726.8 (80.9-1686.1) | 0 (-0.01-0.02) |
| Taiwan (Province of China) | 59921.3 (6126.6-136379.6) | 649.4 (66.4-1478.1) | 50798.4 (5397-114296.7) | 673.3 (71.5-1514.9) | 0.16 (0.13-0.18) |
| Bosnia and Herzegovina | 13015.7 (2055.9-30880) | 685.4 (108.3-1626) | 6989.3 (1115.3-16388.4) | 694.6 (110.8-1628.6) | 0.03 (0.01-0.04) |
| Rwanda | 12698.3 (2226.6-29921.3) | 463.3 (81.2-1091.8) | 26500.1 (4608-60991.5) | 467.3 (81.2-1075.4) | 0.05 (0.02-0.09) |
| Republic of Korea | 137921.9 (18133.7-329040) | 655.3 (86.2-1563.3) | 102587.1 (14045.9-245332.8) | 641.1 (87.8-1533.2) | -0.24 (-0.29--0.18) |
| Bangladesh | 303624.1 (28080.3-694357.7) | 719.3 (66.5-1645) | 506182.8 (47273.3-1151074.1) | 735.6 (68.7-1672.7) | 0.09 (0.08-0.09) |
| Bahrain | 2035 (319.6-4528.9) | 793.9 (124.7-1766.8) | 5462.5 (851.2-12114.9) | 776.4 (121-1721.9) | -0.13 (-0.15--0.1) |
| Singapore | 7042 (1272.9-16200.8) | 466.6 (84.3-1073.4) | 10512.3 (1796.8-23705.4) | 546.5 (93.4-1232.3) | 0.55 (0.41-0.7) |
| Armenia | 9947.1 (1293.1-23648.2) | 692.1 (90-1645.4) | 7538.9 (1027-17835.3) | 701.3 (95.5-1659) | 0.05 (0.02-0.07) |
| Belarus | 27354.5 (5486.4-61621.8) | 693.5 (139.1-1562.2) | 20574.3 (4231.4-45954.9) | 701.3 (144.2-1566.5) | 0.05 (0.02-0.08) |
| Estonia | 3917.1 (776.1-8855) | 689.7 (136.6-1559) | 2740.8 (568.1-6077.9) | 693.1 (143.7-1536.9) | 0.02 (0-0.04) |
| France | 210203.8 (23378.8-469998.6) | 955.4 (106.3-2136.3) | 187597.6 (21270.6-412973.7) | 944.3 (107.1-2078.7) | -0.04 (-0.07--0.02) |
| Bulgaria | 20599.9 (3204.7-48778.9) | 692 (107.7-1638.6) | 13247.5 (2105.3-30682.4) | 697.4 (110.8-1615.2) | 0.06 (0.05-0.07) |
| Croatia | 12605.9 (2043.9-29961.8) | 694.7 (112.6-1651.2) | 8707 (1378.9-20310.1) | 697.6 (110.5-1627.2) | -0.04 (-0.14-0.05) |
| Antigua and Barbuda | 191.2 (19.7-434.3) | 741.3 (76.3-1684) | 253.1 (27-577.2) | 735.8 (78.6-1678) | -0.03 (-0.05--0.01) |
| Guam | 457.4 (43.1-1073.9) | 721.2 (67.9-1693) | 401.3 (38.7-927.6) | 723.8 (69.8-1673.2) | -0.01 (-0.03-0) |
| Zimbabwe | 24847.2 (3175.1-56647.8) | 626.7 (80.1-1428.8) | 40260.4 (5277.9-92063.5) | 635.3 (83.3-1452.6) | 0.08 (0.06-0.09) |
| United States Virgin Islands | 296.4 (30.2-674.3) | 748.3 (76.2-1702.3) | 171 (17.7-381.4) | 739.3 (76.5-1649.2) | -0.03 (-0.04--0.01) |
| Bahamas | 870.8 (88.8-1986.4) | 737.9 (75.2-1683.2) | 1140.8 (118.1-2583.7) | 737.6 (76.4-1670.5) | 0.01 (0-0.01) |
| Germany | 296959.1 (31172.3-677351.9) | 999.6 (104.9-2280) | 257686.7 (26596.7-590341) | 1018.6 (105.1-2333.5) | -0.01 (-0.09-0.07) |
| Malaysia | 55202.4 (5056-127763.8) | 743.8 (68.1-1721.4) | 105962.5 (9314.3-246183.2) | 762.2 (67-1770.8) | 0.1 (0.08-0.12) |
| Spain | 144962.3 (14933.9-330851.4) | 977.5 (100.7-2231) | 123276.8 (14019.3-274381.2) | 992.9 (112.9-2209.9) | 0.1 (0.08-0.11) |
| Micronesia (Federated States of) | 286.8 (27.3-680.7) | 715.9 (68.1-1699) | 304.4 (28.2-718.9) | 716.7 (66.5-1692.8) | 0.01 (0-0.01) |
| Greece | 36680.1 (3500.9-82801.7) | 975.7 (93.1-2202.6) | 27222.5 (2623.3-62245.1) | 977.9 (94.2-2236) | 0.04 (0.02-0.05) |
| Saint Vincent and the Grenadines | 335.9 (34-765.1) | 731.2 (74.1-1665.8) | 301.3 (32.1-683.4) | 729.2 (77.7-1653.9) | 0.01 (0-0.01) |
| Suriname | 1182.4 (117.1-2685.1) | 726.1 (71.9-1648.9) | 1566.4 (169.1-3545.9) | 729.7 (78.8-1651.9) | 0.03 (0.03-0.04) |
| Sweden | 27937.4 (2633.2-63589.7) | 951.7 (89.7-2166.3) | 30885.5 (2885.9-69136.6) | 952.8 (89-2132.9) | 0 (-0.01-0.01) |
| Syrian Arab Republic | 39401.7 (5787.8-90450.7) | 821.3 (120.6-1885.4) | 42829.7 (6190.2-97831.5) | 842.1 (121.7-1923.5) | 0.12 (0.09-0.15) |
| Guatemala | 21092 (2195.3-48138.2) | 714 (74.3-1629.6) | 48967 (5107.9-109610.4) | 719.4 (75-1610.3) | 0.04 (0.03-0.04) |
| Tunisia | 28603.3 (4175.8-64299.3) | 831.5 (121.4-1869.3) | 36800 (5593.4-81181) | 845.6 (128.5-1865.5) | 0.08 (0.07-0.09) |
| Honduras | 12459.3 (1295.1-28243.3) | 721.7 (75-1635.9) | 31858.5 (3308.4-71558.9) | 724.8 (75.3-1628) | 0.03 (0.03-0.04) |
| Turkey | 195875.9 (30790.1-441425.2) | 818.9 (128.7-1845.5) | 263561.7 (44024.9-590009.6) | 827.3 (138.2-1851.9) | 0.06 (0.02-0.09) |
| Mexico | 258208.7 (28694.9-591691.5) | 724 (80.5-1659.2) | 376560.9 (41920.1-849450.6) | 731 (81.4-1649) | 0.04 (0.03-0.05) |
| Switzerland | 22293.1 (2370.8-50872.1) | 846.1 (90-1930.7) | 24033.9 (2642.4-52200) | 865.6 (95.2-1880) | 0.07 (0.06-0.07) |
| Cabo Verde | 975.6 (106.9-2294.5) | 746.1 (81.8-1754.7) | 1865.5 (217-4284) | 744.3 (86.6-1709.4) | -0.01 (-0.02-0.01) |
| Zambia | 15322.4 (2746.3-36301.7) | 505 (90.5-1196.4) | 41741.5 (7649.5-97712.1) | 515.8 (94.5-1207.4) | 0.05 (0.02-0.07) |
| Chad | 15500.1 (1717.4-36322.1) | 738.4 (81.8-1730.4) | 46376 (5108.8-108587.7) | 737 (81.2-1725.6) | 0 (0-0.01) |
| Northern Mariana Islands | 172.7 (16.6-405.6) | 737.6 (71-1731.7) | 119.6 (11.2-278.4) | 725.1 (67.9-1688.3) | -0.15 (-0.22--0.08) |
| Palau | 50.3 (4.7-117.4) | 721.6 (68.1-1683.1) | 41.7 (4.1-96.1) | 707.2 (69.2-1631.1) | -0.08 (-0.11--0.06) |
| Sao Tome and Principe | 317.1 (35.3-750.4) | 738.6 (82.1-1748.1) | 675.2 (77-1573.5) | 742.9 (84.7-1731.2) | 0.03 (0.03-0.04) |
| Pakistan | 293995.3 (25731.4-682612.7) | 720.5 (63.1-1672.9) | 711412.9 (53295.3-1655294.3) | 719.3 (53.9-1673.6) | -0.04 (-0.07--0.02) |
| Egypt | 183478.7 (26594.1-413843.2) | 836.9 (121.3-1887.8) | 369719.5 (51797.1-823128) | 875.9 (122.7-1950) | 0.21 (0.17-0.24) |
| North Macedonia | 5460.4 (842.4-12995.8) | 687.6 (106.1-1636.5) | 5309.3 (873.3-12415.9) | 694.2 (114.2-1623.5) | 0.04 (0.04-0.05) |
| Senegal | 20342.7 (2223.6-46919.7) | 737.2 (80.6-1700.3) | 47533.7 (5332.7-110948.6) | 737.2 (82.7-1720.7) | 0.01 (0.01-0.02) |
| Iran (Islamic Republic of) | 189011.6 (28648.6-421312.1) | 870.3 (131.9-1939.9) | 314044.8 (48840.4-685775.7) | 904.9 (140.7-1976.1) | 0.13 (0.06-0.19) |
| Eritrea | 5884.6 (1023.4-13886.9) | 454.1 (79-1071.6) | 13002.4 (2317.9-30230.8) | 464.4 (82.8-1079.7) | 0.1 (0.09-0.11) |
| Kyrgyzstan | 12287.6 (1639.1-29767.9) | 681.3 (90.9-1650.5) | 18711 (2504.4-44589) | 687.5 (92-1638.4) | 0.03 (0.01-0.05) |
| Ethiopia | 76331.2 (12597.9-177644.5) | 417.8 (69-972.3) | 191924.4 (29707-456601.6) | 414 (64.1-985) | 0 (-0.01-0.01) |
| Republic of Moldova | 12168.8 (2449.7-27423.8) | 698.2 (140.6-1573.5) | 8742.3 (1825.4-19447.6) | 704.9 (147.2-1568) | 0.04 (0-0.08) |
